# Association of Antimicrobial Resistance in *Campylobacter* spp. in Broilers and Turkeys with Antimicrobial Use

**DOI:** 10.3390/antibiotics10060673

**Published:** 2021-06-04

**Authors:** Bernd-Alois Tenhagen, Matthias Flor, Katja Alt, Marie-Theres Knüver, Christiane Buhler, Annemarie Käsbohrer, Kerstin Stingl

**Affiliations:** 1 Unit Epidemiology, Zoonoses and Antimicrobial Resistance, Department Biological Safety, German Federal Institute for Risk Assessment, Max-Dohrn-Str. 8–10, 10589 Berlin, Germany; Matthias.Flor@bfr.bund.de (M.F.); Katja.Alt@bfr.bund.de (K.A.); Annemarie.kaesbohrer@bfr.bund.de (A.K.); 2 Unit Food Microbiology, Pathogen-Host Interactions, Department Biological Safety, German Federal Institute for Risk Assessment, Max-Dohrn-Str. 8–10, 10589 Berlin, Germany; Marie-Theres.knuever@bfr.bund.de (M.-T.K.); Christiane.Buhler@bfr.bund.de (C.B.); Kerstin.Stingl@bfr.bund.de (K.S.)

**Keywords:** Campylobacter, antimicrobial resistance, antimicrobial use, poultry

## Abstract

We investigated trends in antimicrobial resistance (AMR) in *Campylobacter* *jejuni* and *Campylobacter* *coli* in poultry between 2010 and 2016 in Germany and their association with antimicrobial use. *Campylobacter* had been isolated from the caeca of broilers and turkeys at slaughter by regional laboratories according to current ISO methods in the framework of a national monitoring program. Isolates were submitted to the National Reference Laboratory for *Campylobacter* and tested for AMR using broth microdilution methods. Minimum inhibitory concentrations were evaluated according to epidemiological cut-off values. Antimicrobial use (AMU) data from 2014 to 2016 were taken from a government report. AMR was higher in *C. coli* than in *C. jejuni* and higher in turkeys than in broilers. AMR was highest to tetracycline and the tested (fluoro)quinolones while it was rare to gentamicin in both bacterial species, infrequent to erythromycin in *C. jejuni*, and moderate in *C. coli*. AMR to tetracycline and erythromycin decreased over time while it increased to (fluoro)quinolones. An association of AMU and AMR was observed for resistance to tetracycline and erythromycin, while it was not observed for the aminoglycosides. Resistance to nalidixic acid and ciprofloxacin increased despite a decrease of fluoroquinolone use between 2014 and 2016, indicating that other factors have a strong influence on resistance to (fluoro)quinolones in *Campylobacter*.

## 1. Introduction

Campylobacteriosis is the most frequent foodborne enteric disease in humans in Germany and the European Union (EU) [1]. Most *Campylobacter* infections are self-limiting but severe and systemic infections may require antimicrobial treatment. A recent study observed that in around one third of the cases, campylobacteriosis was treated with antimicrobials in Germany [2]. Therefore, from a public health perspective, limiting antimicrobial resistance (AMR) in *Campylobacter* spp. is of utmost importance. As most *Campylobacter* infections in humans are foodborne, AMR in *Campylobacter* from animals and food has a substantial impact on AMR in *Campylobacter* causing infections in humans [3]. This is especially valid for *Campylobacter* from poultry, as several studies have attributed poultry a major role as a source of human infections [2,4].

In recent years, AMR in *Campylobacter* from poultry in Europe was monitored based on Commission Implementing Decision 2013/652/EU [5]. In that framework, AMR investigation in *C. jejuni* from broilers and turkeys was mandatory every two years in Germany. To this end, caecal samples from broilers and turkeys were collected at slaughter and tested for thermophilic *Campylobacter* spp. Sampling plans had to be designed to collect a minimum of 170 isolates of *C. jejuni* from each of these poultry populations [5]. In a number of countries, an increase of resistance of *C. jejuni* from broilers to tetracycline and ciprofloxacin was observed. This was less frequent in isolates from turkeys; however, monitoring of AMR is not mandatory in all countries. Likewise, *C. jejuni* isolates from humans showed increasing resistance to ciprofloxacin and tetracyclin in a number of countries [6]. In Germany, active monitoring of AMR in *Campylobacter* spp. from poultry had already been carried out since 2010, albeit at other intervals. The EU-harmonized protocol is supplemented by additional testing of *C. coli* collected within the same sampling framework.

Antimicrobial use (AMU) is known to be one of the main drivers for AMR in bacteria. As the prevalence of *Campylobacter* spp. in the German broiler population is typically high and *Campylobacter* spp. is spread among flocks and their environments, any AMU in poultry can be expected to exert selective pressure, favoring the colonization of resistant *Campylobacter* spp. in these poultry populations. Vice versa, a reduction of AMU in poultry may lead to a subsequent reduction of AMR in *Campylobacter* from these populations. Such an association of use and AMR has been shown for *E. coli* in several studies and animal species [7,8,9].

For *Campylobacter,* however, the situation seems to be more complex. A joint report by the European Food Safety Authority (EFSA), the European Center for Disease Control and Prevention (ECDC), and the European Medicines Agency (EMA) on the association of AMU and AMR in animals and humans has pointed out that on a national scale, use of fluoroquinolones in poultry and resistance to the fluoroquinolone ciprofloxacin in *C. jejuni* from poultry were positively associated [3]. However, analyses comparing resistance prevalence in isolates from conventional and organic turkey meat found significant differences for *C. jejuni*, but not for *C. coli* [10]. Likewise, a recent study demonstrated differences in AMR of *Campylobacter* spp. originating from organic and conventional pig production for France, but not for Sweden [11]. In addition, a study from the UK found that the resistance level of *Campylobacter* spp. to fluoroquinolones was influenced by AMU on the poultry farm, but that it was also present on many farms that never used fluoroquinolones [8].

It was the objective of this study to investigate whether changes in the use of antimicrobials in broilers and turkeys in the course of the “German antimicrobial minimization strategy” were associated with changes in resistance of *C. jejuni* and *C. coli* from these poultry species. Our hypothesis was that use of antimicrobials would be positively associated with resistance, i.e., increases and decreases in use would be associated with increases and decreases in resistance, respectively. For the years 2010 to 2013, no species-specific AMU data were available, but the overall decrease in antimicrobial sales to veterinarians indicated decreasing use [12]. For the period from the second half of 2014 to 2016, we could use species and antimicrobial class-specific use data to test our hypothesis.

## 2. Results

### 2.1. Development of AMU in Broilers and Turkeys

Among the considered substances, AMU in broilers was highest for aminoglycosides, followed by fluoroquinolones (Table 1). Compared to the first observation period (i.e., second half of 2014), AMU was lower for all recorded substances in 2016 than in 2014. Total AMU in broilers decreased from a treatment frequency (TF) of 26.0 in the second half of 2014 to a TF of 17.4 in 2016 (−33.0%). TF is the number of days an average animal is under treatment with an antibiotic in a six month period (for details see material and methods). The biggest decrease among the specifically included substance classes was observed for macrolides (−62%), and tetracyclines (−58%). The smallest decrease was observed for aminoglycosides (−27%) and fluoroquinolones (−15%).

Among the considered substance groups, the most frequently used drug class in turkeys was the fluoroquinolones, followed by tetracyclines and macrolides. Aminoglycosides were less frequently used in turkeys than the other substances (Table 1).

In turkeys, total use decreased from a TF of 33.0 in 2014 to 23.4 in 2016 (−29%). Among the frequently used substance classes with relevance to *Campylobacter,* the reduction was biggest for macrolides (−38%), followed by tetracyclines (−28%) and fluoroquinolones (−26%). In contrast, use of aminoglycosides increased slightly in the observation period (+4%). However, in contrast to the situation in broilers, use of aminoglycosides was low in turkeys during the whole observation period (Table 1).

### 2.2. Development of AMR

A total of 1792 isolates were tested for their resistance to the six antimicrobials. Of those, 659 were from broilers (191 *C. coli*, 468 *C. jejuni*) and 1133 from turkeys (587 *C. coli*, 546 *C. jejuni*). Results from resistance testing are shown in Table 2. Resistance in both animal and bacterial species was highest to the (fluoro)quinolones ciprofloxacin and nalidixic acid as well as to tetracycline. For gentamicin, there were only two resistant isolates, one *C. jejuni* from broilers in 2016 and one *C. coli* from turkeys in 2014. Therefore, gentamicin was not included in the logistic regression models.

Full susceptibility was more frequent in *C. jejuni* than in *C. coli*, and it was more likely to be observed in isolates from broilers than from turkeys in both models (Table 3 and Table 4). The proportion of fully susceptible isolates did not change significantly over the years in broilers and turkeys (Table 3).

Resistance to each of the antimicrobial substances was more likely to be observed in isolates of *C. coli* than in *C. jejuni*, with odds ratios ranging between 4.2 for nalidixic acid and 26.2 for erythromycin. Resistance to fluoroquinolones increased over the years, while resistance to erythromycin and tetracycline decreased. Resistance to streptomycin did not change significantly in the full model.

Differences between the two animal species were observed for the proportion of fully susceptible isolates and for all substances but aminoglycosides. Resistance was higher in turkeys than in broilers. For both aminoglycosides, the difference was not significant (Table 3).

Stratified analysis considering the two animal species separately confirmed the unchanged proportion of fully susceptible strains for both animal species over time. It was likewise confirmed for both bacterial species when they were considered separately (Tables S1 and S2).

Resistance to streptomycin did not show a significant trend over the years in either animal or bacterial species.

Resistance to nalidixic acid increased significantly in *C. jejuni* in both animal species between 2010/2011 and 2016, but not in *C. coli*. The increase in resistance to ciprofloxacin was not significant in both bacterial and animal species over this time period when they were considered separately. Resistance levels to (fluoro-)quinolones of *C. jejuni* ranged between 42.5% and 77.6% and in *C. coli* between 79.5% and 95.3% in the individual monitoring years (Table 2).

Resistance to tetracycline decreased in isolates of both *Campylobacter* spp. from turkeys, but showed no significant trend in isolates from broilers (Tables S1 and S2).

Resistance to erythromycin decreased substantially in *C. coli* from 2014 to 2016, while resistance in *C. jejuni* was very low during the whole observation time (15/1014 isolates) (Table 2). Likewise, when isolates from both animal species were considered separately, changes in resistance of *C. jejuni* to erythromycin were not significant, but those in *C. coli* were in both animal species.

### 2.3. Association of AMR and AMU

The association of AMU of specific antimicrobial classes with resistance to substances from these classes could be studied on the basis of data from the years 2014 and 2016 (Table 2 and Table 4). There was no significant association of AMU (measured as total AMU) with the proportion of fully susceptible isolates. Resistance to streptomycin was not significantly associated with TF for aminoglycosides in any of the bacteria/animal species combinations.

However, resistance to nalidixic acid in *C. jejuni* was negatively associated with use of fluoroquinolones measured as TF. The same applied for resistance in *C. jejuni* to ciprofloxacin in turkeys, but not in broilers (Table S3). No such association was seen for *C. coli* (Table S4).

Resistance of *C. coli* to tetracycline, on the other hand, was positively associated with use of tetracyclines in turkeys but not in broilers, and no such association was observed for *C. jejuni* (Table S4). Finally, resistance of *C. coli* of both animal species to erythromycin was positively associated with the use of macrolides. Resistance of *C. jejuni* to erythromycin was so rare that no significant associations could be observed with logistic regression.

## 3. Discussion

The objective of this study was to analyze trends in AMR in *Campylobacter* isolates from broilers and turkeys and to study potential associations with AMU in both animal species.

Antimicrobial sales to veterinarians have been decreasing in Germany since 2011 [12,15]. This is in line with decreased sales of antimicrobials for use in animals in a number of other countries [16]. As previously explained in a government report, it could be shown that AMU has decreased in broilers and turkeys between 2014 and 2016 [17]. These detailed data can be considered the best available data on AMU in farm animals in Germany. However, as they were only available for analysis for the interval between 2014 and 2017, and resistance data on isolates from poultry according to CID 2013/652/EU are only collected in even years, the analysis had to be restricted to data from the years 2014 to 2016. We chose to assume that the data of the first half of 2014 were similar to the second half, which is a potential source of bias. It cannot be excluded that AMU was even higher in the first half of the year, i.e., before the implementation of the new legislation. Therefore, the percent change in AMU between the two years may be not accurate. However, only for the use of aminoglycosides in turkeys, this might have affected the direction of change from a slight increase to a slight decrease. Given the minor changes in aminoglycoside use in turkeys, we considered this bias tolerable.

The decrease was observed for most of the considered antimicrobial classes (Table 1). However, it was observed at different levels, and for aminoglycosides there was even a slight increase in TF in turkeys between 2014 and 2016. Moreover, the observed decrease was not always linear. From the data, it is clear that the patterns of AMU also differed between broilers and turkeys, with aminoglycosides being the most frequently used of the investigated substance classes in broilers and fluoroquinolones being most frequently used in turkeys. While the total level of use changed between 2014 and 2016, the share of the different antimicrobial groups considered here did not change substantially. This share differs from data in other countries such as the Netherlands, where use of fluoroquinolones has substantially dropped relative to other classes [18]. It has been previously pointed out that preferences for antimicrobials prescribed for broilers differ substantially between countries [19].

The level of AMU was higher in turkeys than in broilers in the considered time interval, which is in line with reports from other countries [18,20]. That applies for the total use, but also for most substance classes that are relevant to *Campylobacter*, although AMU in turkeys was most frequent for drugs that do not have a direct selection effect on *Campylobacter* or where resistance was not studied in the monitoring (i.e., penicillins and polymyxins, data not shown).

In particular, TF with quinolones, macrolides, or tetracyclines decreased in turkeys during the indicated time period to similar levels reported for broilers in 2014 at the start of the AMU observation period. An exception was the use of aminoglycosides that was far more frequent in broilers. This was mainly due to a high use of the combination of lincomycin with the aminoglycoside spectinomycin [13]. Differences in AMU patterns between the two animal species indicate that it is not advisable to consider them together as “poultry”, which is frequently done in the absence of stratified data [21].

Antimicrobial resistance was higher in *C. coli* than in *C. jejuni*, with the exception of resistance to gentamicin, which was rarely seen in both bacterial species originating from both animal species. This finding is in line with previous studies [10], with data collected in the European monitoring systems from food producing animals and humans [6] and with international data [22]. However, some studies also found higher resistance to tetracycline for isolates of *C. jejuni* from broilers as compared to *C. coli* [23].

Resistance was also higher in isolates from turkeys than in isolates from broilers, with the exception of resistance to the aminoglycosides streptomycin and gentamicin, which did not differ between the isolates from the two animal species. The differences between broilers and turkeys are in line with the pattern of higher levels of use in turkeys for most relevant substances. Given the differences in use of aminoglycosides, a higher resistance in broilers to gentamicin and streptomycin could be expected. Overall, differences in resistance between broilers and turkeys are not pronounced in Europe [6].

In both bacterial and animal species, resistance was highest to fluoroquinolones and tetracycline, which is in line with data from other European countries for the time period [24]. In turkeys, this is consistent with fluoroquinolones and tetracyclines being the most frequently used antimicrobials among the antimicrobials considered in this study (Table 1). In broilers, however, use of tetracycline was low and use of aminoglycosides was substantially higher than that of fluoroquinolones (Table 1).

The AMR characteristics in the two bacterial species isolated from the same animal species were different during the whole observation period. Resistance to nalidixic acid increased in *C. jejuni* from turkeys and broilers, but not in *C. coli*. However, it has to be noted that resistance of *C. coli* to fluoroquinolones was already above 87% in both animal species in the first year of observation (2010 for turkeys and 2011 for broilers), making a further increase unlikely to be statistically significant. The proportion of *C. coli* isolates from turkeys that was resistant to nalidixic acid was ≥ 87% at any time between 2011 and 2016. In contrast, *C. jejuni* exhibited resistance rates to nalidixic acid <75% throughout the observation period. Resistance to ciprofloxacin did not increase significantly in either animal or bacterial species when considered separately (Table 2). Resistance rates to fluoroquinolones in *C. jejuni* from both species were between 58% and 71% in 2010 and 2011. A numerical increase in resistance to ciprofloxacin and nalidixic acid was observed in *C. jejuni* between 2014 and 2016 despite decreasing use of fluoroquinolones in that period. This is, on first sight, counterintuitive. A possible reason is that the level of use in 2015 and 2016 was, despite the decreasing overall trend, still high enough to support resistance selection in the population. However, so far, the use level of fluoroquinolones that would not continue selecting resistant *C. jejuni* remains to be determined. Data from the Netherlands indicate that resistance of *C. jejuni* from broilers to ciprofloxacin may even increase when the amount of use of fluoroquinolones in the population is very low [24,25]. Increases in resistance of *Campylobacter* spp. to fluoroquinolones have also been observed by others that likewise did not report a parallel increase in fluoroquinolone use [26]. It has been demonstrated that resistance to fluoroquinolones is associated with other selective advantages of distinct *C. jejuni* strains for colonization of broilers [27], leading to an increase in resistance despite reduced use. Furthermore, clonal spreading of (fluoro)quinolone-resistant *C. jejuni* was observed in European countries [28,29]. However, these associations are embedded in complex relationships that require further study [30]. On the other hand, on-farm use of fluoroquinolones was shown to be the major risk factor for ciprofloxacin resistant *Campylobacter* spp. on broiler farms [8]. Increases in resistance to fluoroquinolones in *Campylobacter* spp. are of concern, as resistance in *Campylobacter* from animals has been shown to be associated with resistance of *Campylobacter* from human infections [3]. Moreover, fluoroquinolones are among WHOs “Highest Priority Critically Important Antimicrobials” (HPCIA) [31].

Resistance to the aminoglycoside streptomycin remained unchanged in both *Campylobacter* spp. from both animal species. Streptomycin resistance was observed in both bacterial and animal species in all years. However, it did not differ between the animal species despite substantial differences in aminoglycoside use in broilers and turkeys (Table 1). The proportion of resistant isolates is in line with reports from the US for poultry meat [32] and within the range of reported resistance in *Campylobacter* in other EU member states [6] and international data [22]. Aminoglycoside use did only decrease temporarily in broilers, and it even increased slightly in turkeys, which is in line with unchanged resistance rates.

Resistance to gentamicin, the other aminoglycoside included in the testing, was close to zero in both animal and bacterial species at all times, with only one isolate of *C. jejuni* from broilers and one isolate of *C. coli* from turkeys being resistant. Low resistance in *Campylobacter* spp. to gentamicin is consistent with reports from other EU member states [6] and Canada [23,26]. Resistance in humans in Europe tends to be higher but is still in the range of 0–1.5% for both *Campylobacter* spp. [6].

The decrease of resistance to erythromycin in *C. coli* from turkeys and broilers over the whole observation period is welcome from a public health perspective, as macrolides are considered HPCIA by WHO [31]. In *C. jejuni*, resistance to erythromycin was rare in broilers and turkeys with only 15 of 1014 resistant isolates (1.5%), which is in agreement with data on resistance of *C. jejuni* from humans and poultry in Europe [6] and *Campylobacter* from broilers in other countries [23]. Resistance to erythromycin in *C. coli* was positively associated with TF for macrolides, i.e., a reduction in macrolide use was associated with a decrease in AMR in *C. coli*. Resistance to macrolides is mainly conferred by a point mutation in the 23S rRNA gene [33]. It was observed that *C. jejuni* strains lose fitness upon acquisition of the point-mutation-based resistance determinant but that this phenomenon does not apply for *C. coli* [34], eventually explaining the observed difference in macrolide resistance prevalence in both species observed in this study. Recently, circulation of an *erm*B resistance cassette was observed in China and Spain, but *erm*B in *Campylobacter* spp. is still rare in Europe [35,36]. Since this resistance mechanism does not seem to interfere with fitness of *C. jejuni*, further reduction of AMU of macrolides in poultry is highly recommended in order to avoid the risk of selection and spread of novel *erm*B carrying clonal lineages.

In isolates from turkeys, a substantial decrease in AMR was observed to tetracycline in both *C. jejuni* and *C. coli* over the whole observation period (2010 to 2016). This is in contrast to data from other countries such as Canada, where increasing resistance to tetracycline was observed in *Campylobacter* from turkeys [26], although there was substantial variation between years. In broilers, resistance of the two bacterial species to tetracycline did not change significantly over time. A reduction of the use of tetracyclines was observed in both animal species between 2014 and 2016, but an associated decrease in AMR was only seen in *C. coli* from turkeys, despite the fact that use tended to be higher in turkeys than in broilers and that the reduction in use was more pronounced in broilers (−62%) than in turkeys (−38%).

The differences in trend between the bacterial species indicate that it is not AMU alone that drives differences in resistance, as both bacterial species are derived from the same overall population and therefore exposed to similar levels of antimicrobial treatment over time. These differences between the bacterial species have also been observed when comparing AMR in *Campylobacter* from organic and conventional turkey meat [10] and in European trend reports [6,24]. As pointed out above, for some antimicrobials, this can be attributed to differences in fitness costs associated with resistance. However, further research is needed to fully understand these differences.

## 4. Materials and Methods

### 4.1. Data on AMR of Campylobacter spp. in Broilers and Turkeys

Antimicrobial resistance data on *Campylobacter* were taken from investigations carried out in the framework of a national monitoring program that is conducted under Directive 2003/99/EC and in compliance with Commission Implementing Decision (CID) 2013/652/EU. The monitoring has been in place since 2009 and is implemented following a specific national legal framework [37]. Before 2014, sampling and testing were carried out in a very similar way to that later prescribed by the CID. Aggregated data are routinely reported in national reports [38,39,40,41,42,43]. Caeca from broilers were included in the monitoring in 2011, 2013, 2014, and 2016. Caeca from turkeys were included in 2010, 2012, 2014, and 2016. Caeca were collected at slaughter. Samples were collected across Germany according to the regional slaughterhouse throughput of the respective animal species. Regional slaughterhouse throughput was obtained from the Federal Office of Statistics (www.destatis.de). One pooled sample, composed of the content of 10 caeca from birds from the same randomly chosen slaughter batch, was tested for *Campylobacter*, and a maximum of one isolate per *Campylobacter* species was submitted for further testing. Samples were tested in the regional laboratories of the federal states according to ISO 10272-1:2017, procedure B, and 1 g of the caecal material was used as test portion. In brief, the samples were 1:10 (*w/v*) diluted in Preston broth and incubated for 24 ± 2 h at 41.5 ± 1 °C under microaerobic atmosphere. After this enrichment step, a loop of 10 µL was spread on modified charcoal cefoperazone deoxycholate agar (mCCDA), and incubation was performed for another 44 ± 4 h under the same conditions. At least one typical or suspect *Campylobacter* colony was confirmed using either phenotypic tests as described in ISO 10272-1:2017 or MALDI-TOF or PCR methods, which were in-house validated in the respective regional laboratories. According to ISO 10272-1:2017, if the first colony was negative, up to four more suspect colonies were checked unless the sample was considered negative for thermophilic *Campylobacter* spp. Per sample, one or two representative isolates of *Campylobacter* spp. were speciated by real-time PCR [44], and one or two (in case both *C. jejuni* and *C. coli* were isolated from the same sample) isolates per sample were tested for AMR according to the prescriptions given in CID 2013/652/EU [5]. For this purpose, strains were subcultured on Columbia blood agar for 24 ± 2 h at 41.5 ± 1 °C under microaerobic atmosphere (5% O_2_, 10% CO_2_, rest N_2_). Broth microdilution susceptibility testing was performed according to M45-A [45] and VET06 [46] with the in-house validated modification of use of fetal calf serum instead of lysed horse blood in the culture medium for improved readability of *Campylobacter* growth. Cation-supplemented Mueller–Hinton broth (MSC Diagnostics, Swalmen, The Netherlands) supplemented with 5% fetal calf serum (PAN Biotech, Aidenbach, Germany) was inoculated with 2–8 × 10^5^ colony-forming units/mL using bacteria grown on Columbia blood agar. Minimum inhibitory concentrations (MICs) were determined for six antimicrobials (Table 2) using the European standardized microtiter plate formats EUCAMP (2010–2013), and EUCAMP2 (2014–2016) (TREK Diagnostic Systems, Altrincham, United Kingdom). Incubation was performed for 44 ± 4 h at 37 °C under microaerobic atmosphere. MICs (mg/L) were semi-automatically analyzed using the Sensititre Vizion system (Thermo Fisher Scientific, Waltham, MA, USA) and the SWIN-Software (Thermo Fisher Scientific, Waltham, MA, USA). MIC were evaluated according to the epidemiological cutoff values (ECOFF) published by the European Committee for Antimicrobial Susceptibility Testing (EUCAST; https://mic.eucast.org/Eucast2 accessed 1 July 2020) and laid down in the CID 2013/652/EU.

### 4.2. Data on AMU in Broilers and Turkeys

Antimicrobial sales data have been collected in Germany since 2011, and data showed a decrease in overall sales from the very beginning [12]. However, the sales data do not allow for an estimation of the use of antimicrobials in specific animal species [3,21].

Specific data on AMU in broilers and turkeys in Germany have been available since July 2014 in the framework of the national data collection on AMU in animals for meat production according to Section 58 a–c of the German Medicinal Products Act [47]. In this section of the German Medicinal Products Act, the national minimization strategy for AMU in animals is defined. This strategy is based on obligatory data reporting and a benchmarking process where AMU in farms is compared to the median and the 3rd quartile of AMU in all farms of the same field of production [17]. The key indicator calculated is a TF per animal per half-year. To this end, farmers are required to report each treatment of their animals with antimicrobials, and data are collected in a central database. Twice a year, i.e., for each half-year period, the database calculates the TF for each farm using the following formula:

(1) $$\mathrm{TF}=\frac{\sum_{i}n\left(i\right)\times d\left(i\right)\times s\left(i\right)}{\bar{N}}$$

where *n*(*i*), *d*(*i*), and *s*(*i*) are the number of animals treated, the treatment duration in days, and the number of active antimicrobial substances, respectively, in the *i*th treatment that was administered on the farm in the half-year period, and where $\bar{N}$ is the average number of animals housed on the farm during that period. In essence, the formula divides the total number of animal days under treatment in the half-year period by the average number of animals housed on the farm during the respective period. To this end, every antimicrobial substance is being counted on its own, i.e., if animals receive two antimicrobials on a given day, this is considered as two treatment days.

While not part of the official minimization strategy, treatment frequencies can also be calculated for specific classes of antimicrobials:

(2) $${\mathrm{TF}}_{c}=\frac{\sum_{i\;}n\left(i\right)\times d\left(i\right)\times s_{c}\left(i\right)}{\bar{N}}$$

where $s_{c}\left(i\right)$ is the number of active substances belonging to class *c* in the *i*th treatment. The sum of all class-specific treatment frequencies retrieves the total TF given by formula (1). While the minimization strategy with its benchmarking process targets farm level AMU, for the present study, we used national level treatment frequencies, i.e., the sums in formulas (1) and (2) are taken over all treatments on all farms in the respective field of production, and the denominator is the sum of all average numbers of animals housed on all farms.

These use data were taken from a supplement to the national report on the evaluation of the legal provisions that was published by the German government in 2019 [13]. Use data had been extracted from the national database and pseudonymized in spring 2018 in the framework of the evaluation process. Data were submitted to extensive plausibility checks to exclude erroneous entries to the best extent possible. Details of these plausibility checks have been reported in a government report [13]. They considered, among others, discrepancies between numbers of animal days under treatment and amount of drug used and implausible animal numbers, indicating erroneous entries. Identified erroneous entries were excluded from the analyses. For our study, we used the total as well as the antimicrobial class-specific national level treatment frequencies provided in the government report [13].

### 4.3. Statistical Analyses

AMR data: For the analysis, MICs from the included isolates were categorized into susceptible and resistant, as prescribed by CID 2013/652/EU, using the epidemiological cut-off values provided by EUCAST and included in the CID. Proportions of resistant isolates are presented as crude percentages with 95% confidence intervals [14]. Two sets of logistic regression analyses were run per antimicrobial using the statistical analysis software SPSS (IBM Deutschland GmbH, 71139 Ehningen Germany). In both analyses the dependent variable was resistant (1) vs. susceptible (0) to the drug in question. In addition to the individual drugs, an outcome variable “full susceptibility” was defined as an isolate susceptible to all tested antimicrobials. It was coded as “fully susceptible (1)” and “not fully susceptible (0)”, i.e., resistant to at least one of the substances tested. This full susceptibility (to a predefined set of substances) parameter has recently been recommended as a proper indicator of AMR in animals [48].

One set of analyses included data from the whole time period, i.e., 2010 to 2016 in order to identify the overall trend in resistance in *Campylobacter* from poultry. Besides the numerical variable year, animal species (broilers = 1, turkeys = 0) and bacterial species (*C. coli* = 1, *C. jejuni* = 0) were included as independent variables.

In a second set of regressions, the outcome variables were identical. However, here the variable “year” was replaced by a variable “use” using the numerical value of the TF for the antimicrobial class that the test substance belonged to in the respective year, i.e., aminoglycosides for streptomycin, macrolides for erythromycin, fluoroquinolones for ciprofloxacin and nalidixic acid, and tetracyclines for tetracycline. For the analysis of full susceptibility, the total TF over all antimicrobials including those that were not considered in the substance specific analysis for the respective year was the use variable.

As specific use data have only been available since July 2014, the association with AMU was only studied using resistance data from 2014 and 2016. Use data in the first half of 2014 were assumed to be similar to the second half of that year. For 2016, the mean between the two TF values for the 6 month periods was calculated and used in the analysis. The change in use of substance classes over time was calculated as the difference of the mean of TF in 2016 and the TF in the second half of 2014 (2014/2) divided by the TF in 2014/2, expressed as a percentage.

Further sub-analyses were run in addition to the two full models to better characterize the observed differences (Supplementary Tables S1–S4). To this end, separate regressions were run for *C. jejuni* and *C. coli* as differences in AMR between the two bacterial species are well established [6,10]. Likewise, separate regressions were run for the two animal species, as from the use data it became clear that use data differ between the species and likewise AMR differed between animal species in the full model. Finally, separate regressions were run for all combinations of animal and bacterial species. For all analyses, *p*-values < 0.05 were considered significant.

## 5. Conclusions

The association of AMU and AMR differed between the two *Campylobacter* spp. and the six antimicrobials considered. Overall, AMU decreased, but this was not associated with a decrease in AMR for all combinations of antimicrobials and bacterial species. Resistance to tetracycline and erythromycin tended to decrease when use decreased. For fluoroquinolones, an opposite trend was observed, which is consistent with the resistance mechanism possibly conferring advantages in distinct *Campylobacter* lineages. This underlines the importance of having detailed use data on the national level. It warrants on the one hand further investigations over longer time periods and on the other hand molecular investigations for a more in-depth understanding of the underlying mechanisms.

## Figures and Tables

**Table 1:** Antimicrobial use by antimicrobial class in broilers and turkeys in Germany from the second half of 2014 to the second half of 2016, measured as treatment frequency (TF). Data from [13].

Species/6 Month Period	Aminoglycosides	Fluoroquinolones	Macrolides	Tetracyclines	Total
Broilers					
2014/2 (July–December)	5.83	1.76	1.05	0.41	25.97
2015/1 (January–June)	4.40	1.88	0.97	0.18	20.42
2015/2 (July–December)	3.28	1.80	0.63	0.15	16.15
2016/1 (January–June)	3.43	1.61	0.49	0.24	16.57
2016/2 (July–December)	5.13	1.38	0.30	0.11	18.25
2016 combined ^1^	4.28	1.50	0.39	0.17	17.41
% change 2014–2016 ^2^	−26.6	−14.7	−62.4	−57.5	−33.0
Turkeys					
2014/2 (July–December)	0.59	3.47	2.08	2.27	33.01
2015/1 (January–June)	0.72	3.48	1.68	1.85	29.21
2015/2 (July–December)	0.50	2.76	1.57	1.98	25.73
2016/1 (January–June)	0.57	2.92	1.27	1.85	24.40
2016/2 (July–December)	0.65	2.19	1.29	1.39	22.37
2016 combined ^1^	0.61	2.55	0.50	1.28	23.39
% change 2014–2016 ^2^	+3.6	−26.5	−38.1	−38.3	−29.2

^1^ mean of 2016/1 and 2016/2; ^2^ % change from 2014/2 to 2016 combined.

**Table 2:** Proportion of resistance (% and 95 % confidence interval [14]) in *C. jejuni* and *C. coli* from broilers at slaughter in Germany to six antimicrobials.

Species, Origin Year No. of Isolates	Nalidixic Acid	Ciprofloxacin	Gentamicin	Streptomycin	Tetracycline	Erythromycin	Fully Susceptible
*C. coli*, Broiler							
2011, N = 25	88.0 (69.2–96.7)	92.0 (73.9–98.9)	0 (0–23.1)	12.0 (3.5–31.0)	80.0 (60.3–91.4)	32.0 (17.2–51.8)	0 (0–16.1)
2013, N = 16	81.3 (56.2–94.2)	81.3 (56.2–94.2)	0 (0–4.1)	6.3 (0–30.6)	68.8 (44.1–85.9)	0 (0–23.1)	12.5 (2.5–37.5)
2014, N = 112	79.5 (71.0–86.0)	82.1 (73.9–88.2)	0 (0–11.2)	12.5 (7.5–20.1)	82.1 (73.9–88.2)	23.2 (16.4–31.9)	6.3 (2.9–12.6)
2016, N = 38	84.2 (69.2–92.9)	92.1 (78.5–98.0)	0 (0–2.4)	18.4 (9.0–33.8)	86.8 (72.1–94.6)	5.3 (0.6–18.4)	2.6 (0–14.9)
*C. coli*, Turkey							
2010, N = 76	92.1 (83.5–96.6)	93.4 (85.2–97.5)	0 (0–5.7)	13.2 (7.2–22.8)	92.1 (83.4–96.6)	64.5 (53.2–74.3)	2.6 (0.2–9.8)
2012, N = 79	87.3 (78.1–93.2)	92.4 (84.1–96.8)	0.4 (0–2.4)	16.5 (9.8–26.4)	93.7 (85.6–97.5)	35.4 (25.8–46.5)	3.8 (0.9–11.1)
2014, N = 263	87.8 (83.3–91.3)	91.6 (87.6–94.5)	0 (0–2.7)	14.1 (10.4–18.8)	88.2 (83.7–91.6)	35.0 (29.5–40.9)	2.7 (1.2–5.5)
2016, N = 169	88.8 (83.0–92.8)	95.3 (90.8–97.7)	0.2 (0–1.1)	13.6 (9.2–19.7)	78.7 (71.9–84.2)	14.2 (9.7–20.4)	2.4 (0.7–6.2)
*C. jejuni*, Broiler							
2011, N = 60	58.3 (45.7–69.9)	65.0 (52.3–75.9)	0 (0–10.4)	5.0 (1.2–14.3)	50.0 (37.7–62.3)	3.3 (0.3–12.0)	18.3 (10.5–30.2)
2013, N = 40	42.5 (28.5–57.8)	47.5 (32.9–62.5)	0 (0–2.2)	2.5 (0–14.0)	32.5 (20.0–48.1)	0 (0–10.4)	42.5 (28.5–57.8)
2014, N = 202	55.0 (48.1–61.7)	65.8 (59.1–72.0)	0.6 (0–3.7)	0 (0–2.2)	50.5 (43.7–57.3)	3.5 (1.6–7.1)	25.2 (19.8–31.7)
2016, N = 166	68.1 (60.6–74.7)	71.7 (64.4–78.0)	0.2 (0–1.3)	3.6 (1.5–7.8)	50.6 (43.1–58.1)	0 (0–2.7)	27.7 (21.5–35.0)
*C. jejuni*, Turkey							
2010, N = 56	60.7 (47.6–72.4)	71.4 (58.4–81.7)	0 (0–5.8)	1.8 (0–10.3)	64.3 (51.2–75.6)	3.6 (0.3–12.8)	19.6 (11.2–32.1)
2012, N = 75	52.0 (40.9–62.9)	68.0 (56.8–77.5)	0 (0–2.1)	9.3 (4.3–18.3)	68.0 (56.8–77.5)	0 (0–5.8)	20.0 (12.5–30.6)
2014, N = 214	57.0 (50.3–63.5)	63.1 (56.4–69.3)	0 (0–2.3)	3.3 (1.5–6.7)	56.1 (49.4–62.6)	1.9 (0.6–4.9)	26.2 (20.7–32.5)
2016, N = 201	73.1 (66.6–78.8)	77.6 (71.3–82.8)	0 (0–0.8)	3.5 (1.6–7.1)	52.2 (45.4–59.0)	0 (0–2.3)	18.4 (13.7–24.4)

**Table 3:** Association of full susceptibility and resistance to five different antimicrobials of *Campylobacter* spp. with bacterial species, animal species, and year. Data from 2010–2016.

Antimicrobial	Covariate	Coefficient of Regression	Standard Error	Wald	df ^1^	*p*-Value	Odds Ratio	95% Confidence Interval of Odds Ratio
Full Suscep-tibility	Year	0.006	0.039	0.025	1	0.875	1.006	0.93–1.09
	*C. coli* vs. *C. jejuni*	−2.149	0.215	100.100	1	<0.001	0.117	0.08–1.18
	Broilers vs. turkeys	0.312	0.140	4.984	1	0.026	1.366	1.04–1.80
	Constant	−13.680	78.706	0.030	1	0.862	0.000	
STR	Year	−0.01	0.05	0.01	1	0.915	0.99	0.9–1.1
	*C. coli* vs. *C. jejuni*	1.54	0.21	53.20	1	<0.001	4.68	3.09–7.09
	Broilers vs. turkeys	−0.26	0.21	1.57	1	0.211	0.77	0.51–1.16
	Constant	7.29	98.89	0.01	1	0.941	1.47 × 10^3^	
NAL	Year	0.08	0.03	7.67	1	0.006	1.09	1.03–1.16
	*C. coli* vs. *C. jejuni*	1.42	0.13	125.54	1	<0.001	4.16	3.24–5.33
	Broilers vs. turkeys	−0.27	0.11	5.47	1	0.019	0.77	0.61–0.96
	Constant	−169.74	61.49	7.62	1	0.006	0.00	
CIP	Year	0.07	0.03	4.99	1	0.025	1.08	1.01–1.15
	*C. coli* vs. *C. jejuni*	1.53	0.15	109.56	1	<0.001	4.60	3.46–6.12
	Broilers vs. turkeys	−0.32	0.12	6.90	1	0.009	0.73	0.57–0.92
	Constant	−147.48	66.41	4.93	1	0.026	0.00	
TET	Year	−0.08	0.03	7.14	1	0.008	0.92	0.87–0.98
	*C. coli* vs. *C. jejuni*	1.55	0.12	163.00	1	<0.001	4.73	3.73–6.01
	Broilers vs. turkeys	−0.33	0.11	8.97	1	0.003	0.72	0.58–0.89
	Constant	165.45	61.81	7.16	1	0.007	7.1 × 10^71^	
ERY	Year	−0.33	0.04	60.89	1	<0.001	0.72	0.66–0.78
	*C. coli* vs. *C. jejuni*	3.27	0.28	140.01	1	<0.001	26.23	15.27–45.05
	Broilers vs. turkeys	−0.53	0.19	7.62	1	0.006	0.59	0.41–0.86
	Constant	659.84	85.04	60.20	1	<0.001	3.6 × 10^286^	

Coding of variables: Resistant (1) vs. susceptible (0) for NAL (nalidixic acid), CIP (ciprofloxacin), ERY (erythromycin), TET (tetracycline), STR (streptomycin); Fully susceptible (1) vs. resistant to ≥1 antimicrobial (0); *C. coli* (1) vs. *C. jejuni* (0); Broilers (1) vs. turkeys (0). **^1^** df: degrees of freedom.

**Table 4:** Association of full susceptibility and resistance to five different antimicrobials of *Campylobacter* spp. with bacterial species, animal species, and antimicrobial use. Data from 2014 and 2016.

Antimicrobial	Covariate	Coefficient of Regression	Standard Error	Wald	df ^1^	*p*-Value	Odds Ratio	95% Confidence Interval of Odds Ratio
Full Suscep-tibility	*C. coli* vs. *C. jejuni*	−2.222	0.251	78.484	1	<0.001	0.108	0.066–0.177
	Broilers vs. turkeys	0.408	0.193	4.469	1	0.035	1.504	1.030–2.194
	TF_total	0.022	0.018	1.541	1	0.214	1.022	0.987–1.058
	Constant	−1.889	0.514	13.523	1	<0.001	0.151	
NAL	*C. coli* vs. *C. jejuni*	1.31	0.15	80.85	1	<0.001	3.71	2.78–4.93
	Broilers vs. turkeys	−1.18	0.30	15.87	1	<0.001	0.31	0.17–0.55
	TF_FQ	−0.64	0.18	12.10	1	<0.001	0.53	0.37–0.76
	Constant	2.62	0.58	20.64	1	<0.001	13.80	
CIP	*C. coli* vs. *C. jejuni*	1.50	0.17	79.86	1	<0.001	4.50	3.24–6.26
	Broilers vs. turkeys	−1.33	0.33	16.16	1	<0.001	0.26	0.14–0.51
	TF_FQ	−0.76	0.20	13.80	1	<0.001	0.47	0.31–0.70
	Constant	3.27	0.64	25.72	1	<0.001	26.23	
ERY	*C. coli* vs. *C. jejuni*	3.01	0.32	87.42	1	<0.001	20.20	10.76–37.94
	Broilers vs. turkeys	1.24	0.36	11.95	1	<0.001	3.45	1.71–6.95
	TF_Macro	1.70	0.30	32.27	1	<0.001	5.49	3.05–9.88
	Constant	−7.16	0.65	121.49	1	<0.001	0.00	
TET	*C. coli* vs. *C. jejuni*	1.52	0.14	123.75	1	<0.001	4.59	3.51–6.00
	Broilers vs. turkeys	0.08	0.16	0.22	1	0.64	1.08	0.79–1.48
	TF_Tet	0.49	0.24	4.05	1	0.04	1.62	1.01–2.61
	Constant	−0.19	0.20	0.94	1	0.33	0.82	
STR	*C. coli* vs. *C. jejuni*	1.85	0.27	48.42	1	<0.001	6.36	3.78–10.72
	Broilers vs. turkeys	2.17	1.23	3.14	1	0.08	8.79	0.79–97.20
	TF_Amino	−0.51	0.27	3.67	1	0.06	0.60	0.36–1.01
	Constant	−3.31	0.28	138.64	1	<0.001	0.04	

Coding of variables: Resistant (1) vs. susceptible (0) for NAL (nalidixic acid), CIP (ciprofloxacin), ERY (erythromycin), TET (tetracycline), STR (streptomycin); Fully susceptible (1) vs. resistant to ≥1 antimicrobial (0); *C. coli* (1) vs. *C. jejuni* (0); Broilers (1) vs. turkeys (0); TF_total: Treatment frequency (TF) considering all substances, TF_FQ: TF with fluoroquinolones, TF_Macro: TF with macrolides, TF_TET: TF with tetracyclines, TF_Amino: TF with aminoglycosides.^1^ df: degrees of freedom.

## Data Availability

Data on AMR are available in the supplementary tables. Data on use are available at: https://www.bmel.de/DE/themen/tiere/tierarzneimittel/kurzfassung16-amg-novelle.html.

## Supplementary Materials

The following are available online at https://www.mdpi.com/article/10.3390/antibiotics10060673/s1. Table S1. Association of year with full susceptibility and resistance to antimicrobials in *C. jejuni* from broilers and turkeys. Table S2. Association of year with full susceptibility and resistance to antimicrobials in *C. coli* from broilers and turkeys. Table S3. Association of therapy frequency with full susceptibility and resistance to antimicrobials in *C. jejuni* from broilers and turkeys. Table S4. Association of therapy frequency with full susceptibility and resistance to antimicrobials in *C. coli* from broilers and turkeys. Table S5: Minimum inhibitory concentrations of isolates included in the analysis.
