# Supplementary material for: Association of Antimicrobial Resistance in Campylobacter spp. in Broilers and Turkeys with Antimicrobial Use
Source: Antibiotics (Basel). 2021 Jun 4;10(6):673. doi: 10.3390/antibiotics10060673 (PMC8227418; doi:10.3390/antibiotics10060673)
Supplement: Supplementary file 1 [file antibiotics-10-00673-s001.zip › antibiotics-1202678 Table S1-S4.pdf]

Supplementary Table S1. Association of year with full susceptibility and resistance to antimicrobials in *C. jejuni* from broilers and turkeys.

	Caecal Samples Broilers			Caecal Samples Turkeys		
	**OR**	**95%CI**	***p***	**OR**	**95%CI**	***p***
Full susceptibility	1.053	0.93–1.2	0.434	0.982	0.88–1.09	0.741
Streptomycin	0.985	0.67–1.45	0.938	0.938	0.76–1.16	0.560
Nalidixic acid	1.137	1.01–1.28	0.028	1.141	1.04–1.25	0.004
Ciprofloxacin	1.106	0.98–1.24	0.093	1.074	0.98–1.18	0.139
Tetracyclin	1.034	0.92–1.16	0.559	0.897	0.82–0.98	0.019
Erythromycin	0.728	0.5–1.06	0.097	0.721	0.49–1.05	0.089

Coding of variables: resistant (1) vs. susceptible (0) for NAL (nalidixic acid), CIP (ciprofloxacin), ERY (erythromycin), TET (tetracycline), STR (streptomycin); fully susceptible (1) vs. resistant to ≥1 antimicrobial (0).

Supplementary Table S2. Association of year with full susceptibility and resistance to antimicrobials in *C. coli* from broilers and turkeys.

	Caecal Samples Broilers			Caecal Samples Turkeys		
	**OR**	**95%CI**	**p**	**OR**	**95%CI**	**p**
Full susceptibility	1.042	0.66–1.64	0.859	0.957	0.74–1.23	0.731
Streptomycin	1.150	0.85–1.56	0.373	0.991	0.88–1.12	0.880
Nalidixic acid	0.954	0.74–1.24	0.720	0.963	0.84–1.1	0.583
Ciprofloxacin	1.016	0.77–1.34	0.909	1.052	0.9–1.23	0.538
Tetracyclin	1.128	0.88–1.45	0.345	0.778	0.67–0.9	0.001
Erythromycin	0.759	0.59–0.97	0.029	0.709	0.65–0.78	0.000

Coding of variables: resistant (1) vs. susceptible (0) for NAL (nalidixic acid), CIP (ciprofloxacin), ERY (erythromycin), TET (tetracycline), STR (streptomycin); fully susceptible (1) vs. resistant to ≥1 antimicrobial (0).

Supplementary Table S3. Association of therapy frequency with full susceptibility and resistance to antimicrobials in *C. jejuni* from broilers and turkeys.

		Caecal Samples Broilers			Caecal Samples Turkeys		
		**OR**	**95%CI**	***p***	**OR**	**95%CI**	***p***
Full susceptibility	TF_total	0.985	0.93–1.04	0.594	1.048	1-1.1	0.059
Streptomycin	TH_Amino	No valid result			No valid result		
Nalidixic acid	TH_FQ	0.117	0.02–0.61	0.011	0.458	0.29-0.72	0.001
Ciprofloxacin	TH_FQ	0.350	0.06–1.94	0.230	0.464	0.29-0.74	0.001
Tetracyclin	TH_Tet	0.982	0.18–5.44	0.984	1.278	0.69-2.36	0.433
Erythromycin	TH_Makro	No valid result			No valid result		

Coding of variables: Resistant (1) vs. susceptible (0) for NAL (nalidixic acid), CIP (ciprofloxacin), ERY (erythromycin), TET (tetracycline), STR (streptomycin); Fully susceptible (1) vs. resistant to ≥1 antimicrobial (0); TF_total: Treatment frequency (TF) considering all substances, TF_FQ: TF with fluoroquinolones, TF_Macro: TF with macrolides, TF_TET: TF with tetracyclines, TF_Amino: TF with aminoglycosides

Supplementary Table S4. Association of therapy frequency with full susceptibility and resistance to antimicrobials in *C. coli* from broilers and turkeys.

		**Caecal samples broilers**			**Caecal samples turkeys**		
		**OR**	**95%CI**	**p**	**OR**	**95%CI**	**p**
Full susceptibility	TF_total	1.111	0.87–1.42	0.406	1.013	0.89–1.15	0.850
Streptomycin	TH_Amino	0.744	0.39–1.41	0.366	No valid result		
Nalidixic acid	TH_FQ	0.291	0.01–12.87	0.523	0.907	0.47–1.75	0.771
Ciprofloxacin	TH_FQ	0.028	0–3.75	0.152	0.516	0.21–1.28	0.153
Tetracyclin	TH_Tet	0.222	0–18.22	0.503	3.066	1.33–7.06	0.008
Erythromycin	TH_Makro	13.025	1.36–124.5	0.026	4.365	2.33–8.16	0.000

Coding of variables: Resistant (1) vs. susceptible (0) for NAL (nalidixic acid), CIP (ciprofloxacin), ERY (erythromycin), TET (tetracycline), STR (streptomycin); Fully susceptible (1) vs. resistant to ≥1 antimicrobial (0); TF_total: Treatment frequency (TF) considering all substances, TF_FQ: TF with fluoroquinolones, TF_Macro: TF with macrolides, TF_TET: TF with tetracyclines, TF_Amino: TF with aminoglycosides
