# Supplementary material for: Association of Antimicrobial Resistance in Campylobacter spp. in Broilers and Turkeys with Antimicrobial Use
Source: Antibiotics (Basel). 2021 Jun 4;10(6):673. doi: 10.3390/antibiotics10060673 (PMC8227418; doi:10.3390/antibiotics10060673)
Supplement: Supplementary file 1 [file antibiotics-10-00673-s001.zip › antibiotics-1202678 Table S5 AMR_Campy_poultry.pdf]

Table S5: Minimum inhibitory concentrations of 6 antimicrobials for isolats of C. jejuni and C. coli from poultry

Year	Sample No.	Matrix_final	Antimicrobial panel	GEN	STR	NAL	CIP	TET	ERY	Species
2010	10C00291	Turkey_caecum_content	EUCAMP	0,25	<=1	>64	>4	<=0,25	<=0,5	C. jejuni
2010	10C00295	Turkey_caecum_content	EUCAMP	0,25	<=1	32	>4	>16	1	C. jejuni
2010	10C00299	Turkey_caecum_content	EUCAMP	0,25	<=1	64	>4	>16	1	C. jejuni
2010	10C00303	Turkey_caecum_content	EUCAMP	0,5	2	64	>4	>16	1	C. coli
2010	10C00305	Turkey_caecum_content	EUCAMP	0,25	<=1	64	>4	<=0,25	2	C. jejuni
2010	10C00307	Turkey_caecum_content	EUCAMP	0,25	<=1	<=2	>4	>16	<=0,5	C. jejuni
2010	10C00309	Turkey_caecum_content	EUCAMP	0,5	2	>64	>4	<=0,25	>32	C. coli
2010	10C00310	Turkey_caecum_content	EUCAMP	0,25	2	32	>4	>16	>32	C. jejuni
2010	10C00312	Turkey_caecum_content	EUCAMP	<=0,125	<=1	32	>4	>16	<=0,5	C. jejuni
2010	10C00313	Turkey_caecum_content	EUCAMP	0,5	1	64	>4	>16	>32	C. coli
2010	10C00315	Turkey_caecum_content	EUCAMP	0,5	2	64	>4	>16	>32	C. coli
2010	10C00317	Turkey_caecum_content	EUCAMP	0,5	2	16	>4	4	1	C. jejuni
2010	10C00318	Turkey_caecum_content	EUCAMP	0,5	4	>64	>4	>16	<=0,5	C. coli
2010	10C00319	Turkey_caecum_content	EUCAMP	0,5	2	64	>4	>16	<=0,5	C. coli
2010	10C00321	Turkey_caecum_content	EUCAMP	0,5	>16	64	>4	>16	>32	C. coli
2010	10C00323	Turkey_caecum_content	EUCAMP	1	4	64	>4	>16	1	C. coli
2010	10C00325	Turkey_caecum_content	EUCAMP	0,5	2	64	>4	>16	<=0,5	C. coli
2010	10C00327	Turkey_caecum_content	EUCAMP	0,25	<=1	<=2	0,125	<=0,25	<=0,5	C. jejuni
2010	10C00334	Turkey_caecum_content	EUCAMP	0,5	1	64	>4	>16	>32	C. coli
2010	10C00335	Turkey_caecum_content	EUCAMP	0,25	<=1	>64	>4	>16	2	C. jejuni
2010	10C00337	Turkey_caecum_content	EUCAMP	0,5	<=1	4	0,25	>16	<=0,5	C. jejuni
2010	10C00339	Turkey_caecum_content	EUCAMP	0,25	<=1	<=2	<=0,06	<=0,25	<=0,5	C. jejuni
2010	10C00341	Turkey_caecum_content	EUCAMP	0,5	2	>64	>4	>16	1	C. coli
2010	10C00343	Turkey_caecum_content	EUCAMP	0,25	<=1	<=2	<=0,06	<=0,25	<=0,5	C. jejuni
2010	10C00345	Turkey_caecum_content	EUCAMP	0,5	4	4	0,125	>16	>32	C. coli
2010	10C00452	Turkey_caecum_content	EUCAMP	0,25	<=1	4	0,25	<=0,25	<=0,5	C. jejuni
2010	10C00571	Turkey_caecum_content	EUCAMP	1	2	16	1	<=0,25	2	C. jejuni
2010	10C00573	Turkey_caecum_content	EUCAMP	0,25	1	32	>4	>16	>32	C. coli
2010	10C00575	Turkey_caecum_content	EUCAMP	0,25	1	32	>4	>16	>32	C. coli
2010	10C00577	Turkey_caecum_content	EUCAMP	0,25	1	64	>4	>16	>32	C. coli
2010	10C00579	Turkey_caecum_content	EUCAMP	0,25	<=1	32	>4	<=0,25	<=0,5	C. jejuni
2010	10C00581	Turkey_caecum_content	EUCAMP	1	4	>64	>4	>16	>32	C. coli

Table S5: Minimum inhibitory concentrations of 6 antimicrobials for isolats of C. jejuni and C. coli from poultry

Year	Sample No.	Matrix_final	Antimicrobial panel	GEN	STR	NAL	CIP	TET	ERY	Species
2010	10C00583	Turkey_caecum_content	EUCAMP	0,5	1	64	>4	>16	>32	C. coli
2010	10C00585	Turkey_caecum_content	EUCAMP	1	>16	>64	>4	>16	>32	C. coli
2010	10C00587	Turkey_caecum_content	EUCAMP	0,25	1	32	>4	>16	>32	C. coli
2010	10C00589	Turkey_caecum_content	EUCAMP	0,5	2	64	>4	>16	>32	C. coli
2010	10C00591	Turkey_caecum_content	EUCAMP	0,5	2	64	>4	>16	>32	C. coli
2010	10C00593	Turkey_caecum_content	EUCAMP	0,5	4	64	>4	>16	<=0,5	C. jejuni
2010	10C00595	Turkey_caecum_content	EUCAMP	0,5	2	64	>4	>16	>32	C. coli
2010	10C00597	Turkey_caecum_content	EUCAMP	0,25	<=1	64	>4	>16	2	C. jejuni
2010	10C00599	Turkey_caecum_content	EUCAMP	0,5	2	64	>4	>16	<=0,5	C. jejuni
2010	10C00601	Turkey_caecum_content	EUCAMP	1	4	8	0,25	<=0,25	2	C. jejuni
2010	10C00603	Turkey_caecum_content	EUCAMP	0,5	4	>64	>4	>16	1	C. jejuni
2010	10C00605	Turkey_caecum_content	EUCAMP	0,5	2	4	0,125	<=0,25	1	C. jejuni
2010	10C00608-1	Turkey_caecum_content	EUCAMP	0,5	2	4	0,25	<=0,25	1	C. jejuni
2010	10C00610	Turkey_caecum_content	EUCAMP	0,5	2	64	>4	>16	>32	C. coli
2010	10C00611	Turkey_caecum_content	EUCAMP	1	2	64	>4	>16	>32	C. coli
2010	10C00613	Turkey_caecum_content	EUCAMP	0,5	2	8	0,5	<=0,25	<=0,5	C. coli
2010	10C00615	Turkey_caecum_content	EUCAMP	0,5	2	64	>4	>16	1	C. coli
2010	10C00617	Turkey_caecum_content	EUCAMP	0,5	2	64	>4	>16	>32	C. coli
2010	10C00619	Turkey_caecum_content	EUCAMP	0,5	2	64	>4	>16	>32	C. coli
2010	10C00623	Turkey_caecum_content	EUCAMP	1	2	64	>4	<=0,25	<=0,5	C. coli
2010	10C00625	Turkey_caecum_content	EUCAMP	0,5	<=1	4	0,25	<=0,25	1	C. jejuni
2010	10C00627	Turkey_caecum_content	EUCAMP	0,5	2	64	>4	>16	>32	C. coli
2010	10C00629	Turkey_caecum_content	EUCAMP	0,5	4	>64	>4	>16	2	C. coli
2010	10C00630	Turkey_caecum_content	EUCAMP	0,5	4	64	>4	>16	1	C. coli
2010	10C00632	Turkey_caecum_content	EUCAMP	0,5	2	4	0,125	<=0,25	<=0,5	C. jejuni
2010	10C00634	Turkey_caecum_content	EUCAMP	0,5	2	64	>4	>16	>32	C. coli
2010	10C00636	Turkey_caecum_content	EUCAMP	0,5	2	64	>4	>16	>32	C. coli
2010	10C00640-1	Turkey_caecum_content	EUCAMP	0,5	<=1	4	0,125	<=0,25	>32	C. jejuni
2010	10C00642	Turkey_caecum_content	EUCAMP	0,5	<=1	>64	>4	>16	<=0,5	C. jejuni
2010	10C00644	Turkey_caecum_content	EUCAMP	0,5	2	64	>4	>16	>32	C. coli
2010	10C00646-1	Turkey_caecum_content	EUCAMP	0,25	<=1	4	0,125	>16	<=0,5	C. jejuni
2010	11C00008	Turkey_caecum_content	EUCAMP	0,5	2	64	>4	>16	>32	C. coli

Table S5: Minimum inhibitory concentrations of 6 antimicrobials for isolats of C. jejuni and C. coli from poultry

Year	Sample No.	Matrix_final	Antimicrobial panel	GEN	STR	NAL	CIP	TET	ERY	Species
2010	11C00010	Turkey_caecum_content	EUCAMP	0,5	2	32	>4	>16	>32	C. coli
2010	11C00012	Turkey_caecum_content	EUCAMP	0,5	>16	64	>4	>16	2	C. jejuni
2010	11C00014	Turkey_caecum_content	EUCAMP	0,25	<=1	32	>4	>16	<=0,5	C. jejuni
2010	11C00016	Turkey_caecum_content	EUCAMP	0,5	>16	>64	>4	>16	1	C. coli
2010	11C00018	Turkey_caecum_content	EUCAMP	0,5	8	>64	>4	>16	1	C. coli
2010	11C00020	Turkey_caecum_content	EUCAMP	<=0,125	<=1	4	0,25	4	<=0,5	C. jejuni
2010	11C00022-1	Turkey_caecum_content	EUCAMP	0,5	2	64	>4	<=0,25	1	C. jejuni
2010	11C00022-2	Turkey_caecum_content	EUCAMP	2	>16	>64	>4	>16	<=0,5	C. coli
2010	11C00024	Turkey_caecum_content	EUCAMP	0,25	<=1	64	>4	>16	<=0,5	C. jejuni
2010	11C00026	Turkey_caecum_content	EUCAMP	0,5	<=1	32	>4	>16	<=0,5	C. jejuni
2010	11C00028	Turkey_caecum_content	EUCAMP	0,5	1	64	>4	>16	1	C. coli
2010	11C00030	Turkey_caecum_content	EUCAMP	0,5	>16	64	>4	>16	>32	C. coli
2010	11C00032-1	Turkey_caecum_content	EUCAMP	<=0,125	<=1	64	>4	16	<=0,5	C. jejuni
2010	11C00032-2	Turkey_caecum_content	EUCAMP	<=0,125	>16	>64	>4	>16	1	C. coli
2010	11C00034	Turkey_caecum_content	EUCAMP	0,25	<=1	64	>4	>16	<=0,5	C. jejuni
2010	11C00036	Turkey_caecum_content	EUCAMP	0,5	2	32	>4	>16	>32	C. coli
2010	11C00038	Turkey_caecum_content	EUCAMP	0,5	2	64	>4	>16	1	C. coli
2010	11C00040	Turkey_caecum_content	EUCAMP	0,5	2	32	>4	>16	>32	C. coli
2010	11C00043	Turkey_caecum_content	EUCAMP	0,5	1	4	0,125	<=0,25	<=0,5	C. coli
2010	11C00045	Turkey_caecum_content	EUCAMP	0,5	2	>64	>4	>16	1	C. coli
2010	11C00048	Turkey_caecum_content	EUCAMP	0,25	<=1	32	>4	>16	<=0,5	C. jejuni
2010	11C00050	Turkey_caecum_content	EUCAMP	0,5	2	64	>4	>16	>32	C. coli
2010	11C00054	Turkey_caecum_content	EUCAMP	0,5	<=1	32	>4	>16	<=0,5	C. jejuni
2010	11C00057	Turkey_caecum_content	EUCAMP	0,5	2	64	>4	>16	>32	C. coli
2010	11C00059	Turkey_caecum_content	EUCAMP	0,5	1	8	0,25	>16	>32	C. coli
2010	11C00061	Turkey_caecum_content	EUCAMP	<=0,125	<=1	64	>4	<=0,25	<=0,5	C. jejuni
2010	11C00063	Turkey_caecum_content	EUCAMP	0,5	1	32	>4	>16	>32	C. coli
2010	11C00064	Turkey_caecum_content	EUCAMP	0,25	1	16	>4	>16	>32	C. coli
2010	11C00070	Turkey_caecum_content	EUCAMP	0,5	1	64	>4	>16	1	C. coli
2010	11C00072	Turkey_caecum_content	EUCAMP	0,5	1	64	>4	>16	>32	C. coli
2010	11C00074	Turkey_caecum_content	EUCAMP	0,25	<=1	64	>4	>16	1	C. jejuni
2010	11C00076	Turkey_caecum_content	EUCAMP	<=0,125	<=1	16	>4	>16	<=0,5	C. jejuni

Table S5: Minimum inhibitory concentrations of 6 antimicrobials for isolats of C. jejuni and C. coli from poultry

Year	Sample No.	Matrix_final	Antimicrobial panel	GEN	STR	NAL	CIP	TET	ERY	Species
2010	11C00077	Turkey_caecum_content	EUCAMP	0,25	<=1	64	4	>16	1	C. jejuni
2010	11C00079	Turkey_caecum_content	EUCAMP	0,5	1	64	>4	>16	>32	C. coli
2010	11C00081	Turkey_caecum_content	EUCAMP	0,25	<=1	64	>4	>16	<=0,5	C. jejuni
2010	11C00083	Turkey_caecum_content	EUCAMP	0,5	2	64	>4	>16	>32	C. coli
2010	11C00086	Turkey_caecum_content	EUCAMP	0,5	4	>64	>4	>16	>32	C. coli
2010	11C00088	Turkey_caecum_content	EUCAMP	0,25	<=1	32	>4	>16	<=0,5	C. jejuni
2010	11C00090	Turkey_caecum_content	EUCAMP	0,5	1	64	>4	>16	>32	C. coli
2010	11C00091	Turkey_caecum_content	EUCAMP	1	1	64	>4	<=0,25	<=0,5	C. coli
2010	11C00092	Turkey_caecum_content	EUCAMP	0,5	<=1	<=2	0,125	>16	<=0,5	C. jejuni
2010	11C00094	Turkey_caecum_content	EUCAMP	0,5	1	64	>4	>16	<=0,5	C. coli
2010	11C00096	Turkey_caecum_content	EUCAMP	0,25	<=1	64	>4	<=0,25	<=0,5	C. jejuni
2010	11C00098	Turkey_caecum_content	EUCAMP	0,25	<=1	>64	>4	0,5	2	C. jejuni
2010	11C00100-1	Turkey_caecum_content	EUCAMP	0,5	2	64	>4	>16	>32	C. coli
2010	11C00100-2	Turkey_caecum_content	EUCAMP	0,25	2	4	0,125	<=0,25	<=0,5	C. jejuni
2010	11C00101	Turkey_caecum_content	EUCAMP	1	2	>64	>4	<=0,25	2	C. coli
2010	11C00103	Turkey_caecum_content	EUCAMP	1	4	64	>4	>16	>32	C. coli
2010	11C00105	Turkey_caecum_content	EUCAMP	0,5	<=1	32	>4	>16	<=0,5	C. jejuni
2010	11C00107	Turkey_caecum_content	EUCAMP	0,5	2	64	>4	>16	>32	C. coli
2010	11C00109	Turkey_caecum_content	EUCAMP	0,5	2	64	>4	>16	>32	C. coli
2010	11C00110	Turkey_caecum_content	EUCAMP	<=0,125	<=1	32	>4	>16	<=0,5	C. jejuni
2010	11C00112	Turkey_caecum_content	EUCAMP	0,5	2	32	>4	>16	>32	C. coli
2010	11C00114	Turkey_caecum_content	EUCAMP	1	2	>64	>4	>16	2	C. coli
2010	11C00116	Turkey_caecum_content	EUCAMP	<=0,125	<=1	64	>4	>16	<=0,5	C. jejuni
2010	11C00118	Turkey_caecum_content	EUCAMP	0,25	2	64	>4	>16	>32	C. coli
2010	11C00120	Turkey_caecum_content	EUCAMP	2	16	>64	>4	>16	2	C. coli
2010	11C00122	Turkey_caecum_content	EUCAMP	0,5	2	64	>4	>16	>32	C. coli
2010	11C00124-1	Turkey_caecum_content	EUCAMP	0,5	4	64	>4	>16	>32	C. coli
2010	11C00124-2	Turkey_caecum_content	EUCAMP	0,25	<=1	<=2	>4	>16	<=0,5	C. jejuni
2010	11C00126	Turkey_caecum_content	EUCAMP	0,25	<=1	64	>4	>16	<=0,5	C. jejuni
2010	11C00131	Turkey_caecum_content	EUCAMP	0,5	2	64	>4	>16	>32	C. coli
2010	11C00133	Turkey_caecum_content	EUCAMP	0,5	2	4	0,25	<=0,25	<=0,5	C. jejuni
2010	11C00135	Turkey_caecum_content	EUCAMP	0,25	<=1	>64	>4	>16	1	C. jejuni

Table S5: Minimum inhibitory concentrations of 6 antimicrobials for isolats of C. jejuni and C. coli from poultry

Year	Sample No.	Matrix_final	Antimicrobial panel	GEN	STR	NAL	CIP	TET	ERY	Species
2010	11C00137	Turkey_caecum_content	EUCAMP	1	2	>64	>4	>16	>32	C. coli
2010	11C00139	Turkey_caecum_content	EUCAMP	<=0,125	<=1	8	1	>16	<=0,5	C. jejuni
2010	11C00141	Turkey_caecum_content	EUCAMP	1	8	>64	>4	>16	2	C. coli
2010	11C00228	Turkey_caecum_content	EUCAMP	1	>16	4	0,125	>16	2	C. coli
2011	11C00375	Broiler_caecum_content	EUCAMP	0,25	>16	4	<=0,06	<=0,25	<=0,5	C. jejuni
2011	11C00377	Broiler_caecum_content	EUCAMP	0,25	1	32	>4	>16	<=0,5	C. jejuni
2011	11C00381	Broiler_caecum_content	EUCAMP	0,5	2	8	0,5	<=0,25	2	C. jejuni
2011	11C00383	Broiler_caecum_content	EUCAMP	0,25	1	64	>4	16	>32	C. jejuni
2011	11C00386	Broiler_caecum_content	EUCAMP	0,5	1	4	0,25	<=0,25	1	C. jejuni
2011	11C00438	Broiler_caecum_content	EUCAMP	1	2	4	0,125	<=0,25	1	C. jejuni
2011	11C00439	Broiler_caecum_content	EUCAMP	0,5	2	4	<=0,06	>16	<=0,5	C. jejuni
2011	11C00460	Broiler_caecum_content	EUCAMP	0,5	2	64	>4	<=0,25	<=0,5	C. coli
2011	11C00470	Broiler_caecum_content	EUCAMP	0,5	2	64	>4	>16	<=0,5	C. coli
2011	11C00517	Broiler_caecum_content	EUCAMP	0,5	1	64	>4	1	1	C. jejuni
2011	11C00518	Broiler_caecum_content	EUCAMP	0,25	1	>64	>4	1	2	C. jejuni
2011	11C00544	Broiler_caecum_content	EUCAMP	0,5	2	4	0,125	<=0,25	1	C. jejuni
2011	11C00569	Broiler_caecum_content	EUCAMP	0,5	>16	64	>4	>16	>32	C. coli
2011	11C00596	Broiler_caecum_content	EUCAMP	1	>16	>64	>4	>16	2	C. coli
2011	11C00597	Broiler_caecum_content	EUCAMP	0,25	>16	32	>4	>16	<=0,5	C. jejuni
2011	11C00604	Broiler_caecum_content	EUCAMP	0,25	1	4	0,125	>16	<=0,5	C. jejuni
2011	11C00658	Broiler_caecum_content	EUCAMP	0,5	1	64	>4	>16	1	C. jejuni
2011	11C00766	Broiler_caecum_content	EUCAMP	<=0,125	8	<=2	<=0,06	<=0,25	<=0,5	C. jejuni
2011	11C00768	Broiler_caecum_content	EUCAMP	0,25	1	32	>4	>16	<=0,5	C. jejuni
2011	11C00772	Broiler_caecum_content	EUCAMP	0,5	2	16	1	0,5	4	C. jejuni
2011	11C00774	Broiler_caecum_content	EUCAMP	0,25	1	32	>4	16	>32	C. jejuni
2011	11C00776	Broiler_caecum_content	EUCAMP	0,25	1	4	0,25	<=0,25	1	C. jejuni
2011	11C00777	Broiler_caecum_content	EUCAMP	0,25	1	64	>4	>16	<=0,5	C. jejuni
2011	11C00779	Broiler_caecum_content	EUCAMP	0,5	1	4	0,125	>16	<=0,5	C. jejuni
2011	11C00780	Broiler_caecum_content	EUCAMP	0,25	1	32	>4	<=0,25	<=0,5	C. jejuni
2011	11C00790	Broiler_caecum_content	EUCAMP	<=0,125	1	8	>4	<=0,25	<=0,5	C. coli
2011	11C00792	Broiler_caecum_content	EUCAMP	0,5	1	4	0,25	<=0,25	1	C. jejuni
2011	11C00793	Broiler_caecum_content	EUCAMP	0,5	1	8	0,5	<=0,25	>32	C. coli

Table S5: Minimum inhibitory concentrations of 6 antimicrobials for isolats of C. jejuni and C. coli from poultry

Year	Sample No.	Matrix_final	Antimicrobial panel	GEN	STR	NAL	CIP	TET	ERY	Species
2011	11C00794	Broiler_caecum_content	EUCAMP	0,5	2	32	>4	>16	>32	C. coli
2011	11C00796	Broiler_caecum_content	EUCAMP	0,5	2	64	>4	<=0,25	<=0,5	C. jejuni
2011	11C00799	Broiler_caecum_content	EUCAMP	0,25	1	64	>4	>16	<=0,5	C. jejuni
2011	11C00802	Broiler_caecum_content	EUCAMP	0,5	2	4	0,5	<=0,25	<=0,5	C. jejuni
2011	11C00803	Broiler_caecum_content	EUCAMP	0,5	1	32	>4	16	<=0,5	C. coli
2011	11C00805	Broiler_caecum_content	EUCAMP	0,25	1	32	>4	>16	<=0,5	C. jejuni
2011	11C00807	Broiler_caecum_content	EUCAMP	0,25	1	<=2	>4	>16	<=0,5	C. jejuni
2011	11C00809	Broiler_caecum_content	EUCAMP	0,5	2	>64	>4	>16	<=0,5	C. jejuni
2011	11C00811	Broiler_caecum_content	EUCAMP	0,25	1	32	>4	<=0,25	<=0,5	C. jejuni
2011	11C00812	Broiler_caecum_content	EUCAMP	0,25	1	64	>4	>16	1	C. jejuni
2011	11C00814	Broiler_caecum_content	EUCAMP	0,5	2	>64	>4	>16	<=0,5	C. coli
2011	11C00816	Broiler_caecum_content	EUCAMP	0,5	>16	64	>4	>16	>32	C. coli
2011	11C00818	Broiler_caecum_content	EUCAMP	<=0,125	1	64	>4	<=0,25	1	C. jejuni
2011	11C00820	Broiler_caecum_content	EUCAMP	<=0,125	1	64	>4	>16	4	C. jejuni
2011	11C00822	Broiler_caecum_content	EUCAMP	0,5	1	64	>4	<=0,25	<=0,5	C. coli
2011	11C00824	Broiler_caecum_content	EUCAMP	0,5	1	64	>4	<=0,25	<=0,5	C. coli
2011	11C00825	Broiler_caecum_content	EUCAMP	0,5	4	64	>4	>16	>32	C. coli
2011	11C00825	Broiler_caecum_content	EUCAMP	0,5	1	<=2	0,125	>16	<=0,5	C. jejuni
2011	11C00827	Broiler_caecum_content	EUCAMP	0,5	2	64	>4	>16	2	C. coli
2011	11C00828	Broiler_caecum_content	EUCAMP	0,5	1	64	>4	>16	2	C. coli
2011	11C00830	Broiler_caecum_content	EUCAMP	0,5	2	64	>4	>16	2	C. coli
2011	11C00832	Broiler_caecum_content	EUCAMP	0,5	1	64	>4	>16	1	C. coli
2011	11C00834	Broiler_caecum_content	EUCAMP	0,25	1	4	0,125	>16	<=0,5	C. jejuni
2011	11C00835	Broiler_caecum_content	EUCAMP	0,25	1	16	>4	>16	<=0,5	C. jejuni
2011	11C00837	Broiler_caecum_content	EUCAMP	0,5	2	64	>4	>16	>32	C. coli
2011	11C00839	Broiler_caecum_content	EUCAMP	0,5	1	4	0,125	>16	1	C. jejuni
2011	11C00841	Broiler_caecum_content	EUCAMP	0,25	1	64	>4	<=0,25	2	C. jejuni
2011	11C01012	Broiler_caecum_content	EUCAMP	1	1	64	>4	<=0,25	1	C. jejuni
2011	11C01013	Broiler_caecum_content	EUCAMP	0,5	1	>64	>4	<=0,25	<=0,5	C. jejuni
2011	12C00025	Broiler_caecum_content	EUCAMP	0,5	2	>64	>4	<=0,25	2	C. jejuni
2011	12C00030	Broiler_caecum_content	EUCAMP	0,5	1	64	>4	>16	2	C. coli
2011	12C00032	Broiler_caecum_content	EUCAMP	0,25	1	32	4	16	<=0,5	C. jejuni

Table S5: Minimum inhibitory concentrations of 6 antimicrobials for isolats of C. jejuni and C. coli from poultry

Year	Sample No.	Matrix_final	Antimicrobial panel	GEN	STR	NAL	CIP	TET	ERY	Species
2011	12C00033	Broiler_caecum_content	EUCAMP	0,5	2	>64	>4	>16	2	C. coli
2011	12C00035	Broiler_caecum_content	EUCAMP	<=0,125	1	32	4	<=0,25	<=0,5	C. jejuni
2011	12C00037	Broiler_caecum_content	EUCAMP	0,25	1	32	>4	>16	2	C. jejuni
2011	12C00038	Broiler_caecum_content	EUCAMP	<=0,125	1	4	0,25	<=0,25	<=0,5	C. jejuni
2011	12C00041	Broiler_caecum_content	EUCAMP	0,5	2	<=2	0,125	<=0,25	<=0,5	C. jejuni
2011	12C00043	Broiler_caecum_content	EUCAMP	0,25	1	16	4	<=0,25	<=0,5	C. jejuni
2011	12C00044	Broiler_caecum_content	EUCAMP	0,25	1	32	>4	>16	<=0,5	C. jejuni
2011	12C00046	Broiler_caecum_content	EUCAMP	<=0,125	1	64	>4	<=0,25	2	C. jejuni
2011	12C00048	Broiler_caecum_content	EUCAMP	0,5	1	64	>4	>16	<=0,5	C. jejuni
2011	12C00050	Broiler_caecum_content	EUCAMP	0,5	2	>64	>4	>16	2	C. coli
2011	12C00053	Broiler_caecum_content	EUCAMP	0,5	2	64	>4	>16	>32	C. coli
2011	12C00055	Broiler_caecum_content	EUCAMP	<=0,125	1	8	0,5	<=0,25	1	C. jejuni
2011	12C00057	Broiler_caecum_content	EUCAMP	0,5	2	64	>4	>16	>32	C. coli
2011	12C00059	Broiler_caecum_content	EUCAMP	0,25	1	>64	>4	>16	2	C. jejuni
2011	12C00061	Broiler_caecum_content	EUCAMP	0,25	1	32	>4	>16	<=0,5	C. jejuni
2011	12C00063	Broiler_caecum_content	EUCAMP	0,25	1	<=2	<=0,06	<=0,25	<=0,5	C. jejuni
2011	12C00066	Broiler_caecum_content	EUCAMP	0,25	1	>64	>4	>16	2	C. jejuni
2011	12C00067	Broiler_caecum_content	EUCAMP	0,5	2	64	>4	>16	2	C. coli
2011	12C00069	Broiler_caecum_content	EUCAMP	0,5	2	>64	>4	<=0,25	<=0,5	C. jejuni
2011	12C00070	Broiler_caecum_content	EUCAMP	<=0,125	1	32	>4	<=0,25	<=0,5	C. jejuni
2011	12C00072	Broiler_caecum_content	EUCAMP	0,25	1	64	>4	>16	1	C. jejuni
2011	12C00075	Broiler_caecum_content	EUCAMP	<=0,125	1	8	0,25	>16	<=0,5	C. jejuni
2011	12C00077	Broiler_caecum_content	EUCAMP	<=0,125	1	4	<=0,06	4	<=0,5	C. jejuni
2011	12C00078	Broiler_caecum_content	EUCAMP	0,25	1	32	4	<=0,25	<=0,5	C. jejuni
2011	12C00308	Broiler_caecum_content	EUCAMP	0,5	2	8	0,25	>16	<=0,5	C. coli
2012	12C00210	Turkey_caecum_content	EUCAMP	0,5	2	8	0,5	<=0,25	1	C. coli
2012	12C00212	Turkey_caecum_content	EUCAMP	0,5	4	>64	>4	>16	2	C. coli
2012	12C00245	Turkey_caecum_content	EUCAMP	0,5	4	64	>4	>16	<=0,5	C. jejuni
2012	12C00247	Turkey_caecum_content	EUCAMP	0,5	2	64	>4	>16	1	C. coli
2012	12C00248	Turkey_caecum_content	EUCAMP	<=0,125	<=1	4	<=0,06	<=0,25	<=0,5	C. jejuni
2012	12C00253	Turkey_caecum_content	EUCAMP	0,25	1	>64	>4	>16	1	C. coli
2012	12C00254	Turkey_caecum_content	EUCAMP	0,5	<=1	>64	>4	>16	<=0,5	C. jejuni

Table S5: Minimum inhibitory concentrations of 6 antimicrobials for isolats of C. jejuni and C. coli from poultry

Year	Sample No.	Matrix_final	Antimicrobial panel	GEN	STR	NAL	CIP	TET	ERY	Species
2012	12C00255	Turkey_caecum_content	EUCAMP	0,25	1	64	>4	>16	>32	C. coli
2012	12C00256	Turkey_caecum_content	EUCAMP	<=0,125	<=1	16	>4	16	<=0,5	C. jejuni
2012	12C00258	Turkey_caecum_content	EUCAMP	0,25	1	64	>4	>16	1	C. coli
2012	12C00259	Turkey_caecum_content	EUCAMP	0,5	2	32	>4	>16	>32	C. coli
2012	12C00265	Turkey_caecum_content	EUCAMP	<=0,125	4	32	>4	>16	<=0,5	C. coli
2012	12C00269	Turkey_caecum_content	EUCAMP	0,5	2	64	>4	>16	2	C. jejuni
2012	12C00271	Turkey_caecum_content	EUCAMP	0,5	1	>64	>4	>16	<=0,5	C. coli
2012	12C00273	Turkey_caecum_content	EUCAMP	0,5	1	32	>4	>16	>32	C. coli
2012	12C00284	Turkey_caecum_content	EUCAMP	0,5	2	>64	>4	<=0,25	<=0,5	C. jejuni
2012	12C00288	Turkey_caecum_content	EUCAMP	<=0,125	<=1	4	0,120	16	<=0,5	C. jejuni
2012	12C00293	Turkey_caecum_content	EUCAMP	<=0,125	<=1	4	<=0,06	<=0,25	<=0,5	C. jejuni
2012	12C00302	Turkey_caecum_content	EUCAMP	0,25	1	32	>4	>16	>32	C. coli
2012	12C00320	Turkey_caecum_content	EUCAMP	0,25	<=1	64	>4	<=0,25	<=0,5	C. jejuni
2012	12C00322	Turkey_caecum_content	EUCAMP	0,5	<=1	64	>4	>16	<=0,5	C. jejuni
2012	12C00326	Turkey_caecum_content	EUCAMP	<=0,125	16	64	>4	>16	<=0,5	C. jejuni
2012	12C00328	Turkey_caecum_content	EUCAMP	1	>16	64	>4	>16	1	C. jejuni
2012	12C00358	Turkey_caecum_content	EUCAMP	0,5	2	64	>4	>16	2	C. coli
2012	12C00359	Turkey_caecum_content	EUCAMP	0,5	1	64	>4	>16	>32	C. coli
2012	12C00360	Turkey_caecum_content	EUCAMP	0,5	4	8	0,25	<=0,25	1	C. coli
2012	12C00361	Turkey_caecum_content	EUCAMP	1	2	32	>4	>16	<=0,5	C. coli
2012	12C00376	Turkey_caecum_content	EUCAMP	0,25	<=1	<=2	0,120	<=0,25	<=0,5	C. jejuni
2012	12C00379	Turkey_caecum_content	EUCAMP	0,25	<=1	8	0,120	<=0,25	<=0,5	C. jejuni
2012	12C00382	Turkey_caecum_content	EUCAMP	1	8	>64	>4	>16	2	C. coli
2012	12C00410	Turkey_caecum_content	EUCAMP	0,5	2	64	>4	>16	<=0,5	C. jejuni
2012	12C00413	Turkey_caecum_content	EUCAMP	<=0,125	2	16	4	>16	<=0,5	C. coli
2012	12C00414	Turkey_caecum_content	EUCAMP	0,5	2	64	>4	<=0,25	<=0,5	C. coli
2012	12C00432	Turkey_caecum_content	EUCAMP	0,5	2	64	>4	>16	<=0,5	C. coli
2012	12C00434	Turkey_caecum_content	EUCAMP	0,5	<=1	64	>4	>16	<=0,5	C. jejuni
2012	12C00436	Turkey_caecum_content	EUCAMP	0,25	<=1	4	0,25	<=0,25	<=0,5	C. jejuni
2012	12C00485	Turkey_caecum_content	EUCAMP	0,5	2	64	>4	>16	2	C. coli
2012	12C00486	Turkey_caecum_content	EUCAMP	0,25	2	64	>4	>16	>32	C. coli
2012	12C00592	Turkey_caecum_content	EUCAMP	0,25	<=1	<=2	>4	>16	<=0,5	C. jejuni

Table S5: Minimum inhibitory concentrations of 6 antimicrobials for isolats of C. jejuni and C. coli from poultry

Year	Sample No.	Matrix_final	Antimicrobial panel	GEN	STR	NAL	CIP	TET	ERY	Species
2012	12C00599	Turkey_caecum_content	EUCAMP	0,5	2	64	>4	>16	4	C. coli
2012	12C00600	Turkey_caecum_content	EUCAMP	0,5	2	4	0,120	<=0,25	1	C. jejuni
2012	12C00601	Turkey_caecum_content	EUCAMP	0,5	2	64	>4	>16	2	C. jejuni
2012	12C00602	Turkey_caecum_content	EUCAMP	0,25	2	32	>4	>16	1	C. jejuni
2012	12C00640	Turkey_caecum_content	EUCAMP	<=0,125	1	32	4	8	<=0,5	C. coli
2012	12C00658	Turkey_caecum_content	EUCAMP	0,5	>16	64	>4	>16	>32	C. coli
2012	12C00661	Turkey_caecum_content	EUCAMP	1	>16	>64	>4	>16	2	C. coli
2012	12C00661	Turkey_caecum_content	EUCAMP	1	>16	64	>4	>16	2	C. jejuni
2012	12C00662	Turkey_caecum_content	EUCAMP	<=0,125	<=1	32	>4	>16	<=0,5	C. jejuni
2012	12C00723	Turkey_caecum_content	EUCAMP	1	4	64	>4	>16	2	C. coli
2012	12C00731	Turkey_caecum_content	EUCAMP	0,5	1	32	>4	>16	>32	C. coli
2012	12C00732	Turkey_caecum_content	EUCAMP	0,5	<=1	32	>4	>16	1	C. jejuni
2012	12C00769	Turkey_caecum_content	EUCAMP	<=0,125	>16	<=2	>4	>16	<=0,5	C. jejuni
2012	12C00771	Turkey_caecum_content	EUCAMP	0,5	4	8	0,120	>16	2	C. coli
2012	12C00772	Turkey_caecum_content	EUCAMP	0,25	2	16	>4	16	1	C. coli
2012	12C00775	Turkey_caecum_content	EUCAMP	0,25	>16	64	>4	>16	4	C. coli
2012	12C00781	Turkey_caecum_content	EUCAMP	0,5	>16	64	>4	>16	8	C. coli
2012	12C00783	Turkey_caecum_content	EUCAMP	0,5	2	4	0,25	<=0,25	1	C. jejuni
2012	12C00785	Turkey_caecum_content	EUCAMP	0,25	>16	16	>4	>16	>32	C. coli
2012	12C00791	Turkey_caecum_content	EUCAMP	0,5	2	64	>4	>16	>32	C. coli
2012	12C00794	Turkey_caecum_content	EUCAMP	0,5	4	>64	>4	>16	>32	C. coli
2012	12C00796	Turkey_caecum_content	EUCAMP	0,5	2	64	>4	>16	>32	C. coli
2012	12C00797	Turkey_caecum_content	EUCAMP	0,25	1	64	>4	>16	>32	C. coli
2012	12C00798	Turkey_caecum_content	EUCAMP	0,25	<=1	32	4	16	1	C. jejuni
2012	12C00800	Turkey_caecum_content	EUCAMP	0,5	2	>64	>4	>16	2	C. coli
2012	12C00801	Turkey_caecum_content	EUCAMP	0,5	2	<=2	0,120	<=0,25	1	C. jejuni
2012	12C00804	Turkey_caecum_content	EUCAMP	0,5	2	64	>4	>16	>32	C. coli
2012	12C00806	Turkey_caecum_content	EUCAMP	0,5	<=1	4	0,120	<=0,25	1	C. jejuni
2012	12C00808	Turkey_caecum_content	EUCAMP	0,5	2	>64	>4	>16	>32	C. coli
2012	12C00809	Turkey_caecum_content	EUCAMP	0,5	2	>64	>4	>16	2	C. coli
2012	12C00810	Turkey_caecum_content	EUCAMP	0,5	1	64	>4	>16	<=0,5	C. coli
2012	12C00811	Turkey_caecum_content	EUCAMP	0,5	1	64	>4	>16	2	C. coli

Table S5: Minimum inhibitory concentrations of 6 antimicrobials for isolats of C. jejuni and C. coli from poultry

Year	Sample No.	Matrix_final	Antimicrobial panel	GEN	STR	NAL	CIP	TET	ERY	Species
2012	12C00812	Turkey_caecum_content	EUCAMP	0,25	>16	64	>4	>16	>32	C. coli
2012	12C00813	Turkey_caecum_content	EUCAMP	0,25	2	>64	>4	>16	2	C. coli
2012	12C00814	Turkey_caecum_content	EUCAMP	0,5	<=1	32	>4	>16	<=0,5	C. jejuni
2012	12C00816	Turkey_caecum_content	EUCAMP	0,5	2	64	>4	>16	2	C. jejuni
2012	12C00818	Turkey_caecum_content	EUCAMP	0,5	<=1	64	>4	>16	<=0,5	C. jejuni
2012	12C00820	Turkey_caecum_content	EUCAMP	0,5	1	64	>4	>16	>32	C. coli
2012	12C00821	Turkey_caecum_content	EUCAMP	0,5	4	8	0,25	>16	<=0,5	C. jejuni
2012	12C00827	Turkey_caecum_content	EUCAMP	0,5	2	64	>4	0,5	2	C. jejuni
2012	12C00834	Turkey_caecum_content	EUCAMP	0,5	2	64	>4	>16	2	C. coli
2012	12C00836	Turkey_caecum_content	EUCAMP	0,25	<=1	4	0,25	<=0,25	<=0,5	C. jejuni
2012	12C00838	Turkey_caecum_content	EUCAMP	<=0,125	>16	<=2	>4	>16	<=0,5	C. jejuni
2012	12C00840	Turkey_caecum_content	EUCAMP	0,5	4	64	>4	>16	>32	C. coli
2012	12C00841	Turkey_caecum_content	EUCAMP	0,5	2	64	>4	>16	>32	C. coli
2012	12C00847	Turkey_caecum_content	EUCAMP	1	2	64	>4	>16	2	C. coli
2012	12C00848	Turkey_caecum_content	EUCAMP	0,5	2	64	>4	>16	1	C. jejuni
2012	12C00850	Turkey_caecum_content	EUCAMP	0,5	2	64	>4	>16	>32	C. coli
2012	12C00851	Turkey_caecum_content	EUCAMP	0,5	2	<=2	>4	<=0,25	<=0,5	C. jejuni
2012	12C00853	Turkey_caecum_content	EUCAMP	0,5	2	64	>4	>16	4	C. coli
2012	12C00856	Turkey_caecum_content	EUCAMP	0,5	<=1	64	>4	>16	2	C. jejuni
2012	12C00857	Turkey_caecum_content	EUCAMP	0,25	4	>64	>4	>16	2	C. coli
2012	12C00859	Turkey_caecum_content	EUCAMP	0,5	2	4	0,25	<=0,25	2	C. jejuni
2012	12C00861	Turkey_caecum_content	EUCAMP	0,25	<=1	8	0,25	<=0,25	2	C. jejuni
2012	12C00868	Turkey_caecum_content	EUCAMP	0,5	2	8	0,5	>16	4	C. coli
2012	12C00870	Turkey_caecum_content	EUCAMP	0,5	<=1	32	>4	<=0,25	<=0,5	C. jejuni
2012	12C00871	Turkey_caecum_content	EUCAMP	<=0,125	1	16	>4	>16	>32	C. coli
2012	12C00873	Turkey_caecum_content	EUCAMP	0,25	<=1	<=2	0,120	>16	<=0,5	C. jejuni
2012	12C00874	Turkey_caecum_content	EUCAMP	0,25	<=1	16	>4	>16	<=0,5	C. jejuni
2012	12C00875	Turkey_caecum_content	EUCAMP	0,5	1	>64	>4	>16	1	C. coli
2012	12C00891	Turkey_caecum_content	EUCAMP	<=0,125	<=1	16	>4	>16	<=0,5	C. jejuni
2012	12C00892	Turkey_caecum_content	EUCAMP	0,5	<=1	8	0,5	>16	<=0,5	C. jejuni
2012	12C00893	Turkey_caecum_content	EUCAMP	0,5	<=1	64	>4	>16	<=0,5	C. jejuni
2012	12C00902	Turkey_caecum_content	EUCAMP	0,25	<=1	4	0,120	0,5	<=0,5	C. jejuni

Table S5: Minimum inhibitory concentrations of 6 antimicrobials for isolats of C. jejuni and C. coli from poultry

Year	Sample No.	Matrix_final	Antimicrobial panel	GEN	STR	NAL	CIP	TET	ERY	Species
2012	12C00918	Turkey_caecum_content	EUCAMP	0,25	<=1	32	>4	>16	<=0,5	C. jejuni
2012	12C00919	Turkey_caecum_content	EUCAMP	0,5	>16	32	>4	>16	<=0,5	C. jejuni
2012	12C00920	Turkey_caecum_content	EUCAMP	0,25	>16	32	>4	>16	<=0,5	C. jejuni
2012	12C00946	Turkey_caecum_content	EUCAMP	0,5	>16	32	>4	>16	1	C. coli
2012	12C00947	Turkey_caecum_content	EUCAMP	0,5	2	>64	>4	>16	1	C. jejuni
2012	12C00966	Turkey_caecum_content	EUCAMP	0,5	4	64	>4	>16	4	C. coli
2012	12C00967	Turkey_caecum_content	EUCAMP	0,25	2	64	>4	>16	2	C. coli
2012	12C00968	Turkey_caecum_content	EUCAMP	0,5	<=1	<=2	>4	<=0,25	<=0,5	C. jejuni
2012	12-CA01022	Turkey_caecum_content	EUCAMP	0,5	2	64	>4	0,5	1	C. coli
2012	12-CA01052	Turkey_caecum_content	EUCAMP	<=0,125	<=1	32	>4	>16	<=0,5	C. jejuni
2012	12-CA01053	Turkey_caecum_content	EUCAMP	0,25	<=1	32	>4	>16	<=0,5	C. jejuni
2012	12-CA01066	Turkey_caecum_content	EUCAMP	<=0,125	<=1	64	>4	>16	1	C. jejuni
2012	12-CA01067	Turkey_caecum_content	EUCAMP	0,5	2	4	0,120	>16	1	C. jejuni
2012	12-CA01072	Turkey_caecum_content	EUCAMP	0,25	>16	32	>4	>16	>32	C. coli
2012	12-CA01078	Turkey_caecum_content	EUCAMP	0,5	2	64	>4	>16	2	C. coli
2012	12-CA01079	Turkey_caecum_content	EUCAMP	0,25	1	8	0,5	>16	<=0,5	C. coli
2012	12-CA01081	Turkey_caecum_content	EUCAMP	0,25	<=1	4	0,120	<=0,25	<=0,5	C. jejuni
2012	12-CA01120	Turkey_caecum_content	EUCAMP	0,25	<=1	32	>4	<=0,25	1	C. jejuni
2012	12-CA01122	Turkey_caecum_content	EUCAMP	0,5	2	64	>4	<=0,25	<=0,5	C. jejuni
2012	12-CA01126	Turkey_caecum_content	EUCAMP	0,5	<=1	<=2	>4	>16	<=0,5	C. jejuni
2012	12-CA01142	Turkey_caecum_content	EUCAMP	0,5	>16	64	>4	>16	>32	C. coli
2012	12-CA01143	Turkey_caecum_content	EUCAMP	0,25	<=1	4	0,120	>16	<=0,5	C. jejuni
2012	12-CA01145	Turkey_caecum_content	EUCAMP	0,25	<=1	32	>4	>16	1	C. jejuni
2012	12-CA01146	Turkey_caecum_content	EUCAMP	<=0,125	<=1	16	>4	>16	1	C. jejuni
2012	13-CA00049	Turkey_caecum_content	EUCAMP	0,5	2	64	>4	>16	2	C. jejuni
2012	13-CA00051	Turkey_caecum_content	EUCAMP	0,25	1	4	0,120	<=0,25	1	C. coli
2012	13-CA00053	Turkey_caecum_content	EUCAMP	0,5	2	>64	>4	>16	>32	C. coli
2012	13-CA00056	Turkey_caecum_content	EUCAMP	0,5	<=1	>64	>4	>16	<=0,5	C. jejuni
2012	13-CA00059	Turkey_caecum_content	EUCAMP	0,5	1	32	>4	>16	1	C. coli
2012	13-CA00067	Turkey_caecum_content	EUCAMP	0,5	<=1	64	>4	>16	<=0,5	C. jejuni
2012	13-CA00072	Turkey_caecum_content	EUCAMP	0,5	>16	64	>4	16	>32	C. coli
2012	13-CA00072	Turkey_caecum_content	EUCAMP	1	2	8	1	>16	4	C. jejuni

Table S5: Minimum inhibitory concentrations of 6 antimicrobials for isolats of C. jejuni and C. coli from poultry

Year	Sample No.	Matrix_final	Antimicrobial panel	GEN	STR	NAL	CIP	TET	ERY	Species
2012	13-CA00078	Turkey_caecum_content	EUCAMP	0,5	<=1	<=2	<=0,06	>16	1	C. jejuni
2012	13-CA00080	Turkey_caecum_content	EUCAMP	0,25	>16	64	>4	>16	>32	C. coli
2012	13-CA00082	Turkey_caecum_content	EUCAMP	0,5	2	4	0,120	>16	1	C. jejuni
2012	13-CA00084	Turkey_caecum_content	EUCAMP	0,5	1	64	>4	>16	1	C. coli
2012	13-CA00086	Turkey_caecum_content	EUCAMP	0,5	1	64	>4	>16	2	C. coli
2012	13-CA00088	Turkey_caecum_content	EUCAMP	0,25	<=1	64	>4	>16	<=0,5	C. jejuni
2012	13-CA00090	Turkey_caecum_content	EUCAMP	0,5	1	>64	>4	>16	>32	C. coli
2012	13-CA00092	Turkey_caecum_content	EUCAMP	0,5	2	>64	>4	>16	2	C. coli
2012	13-CA00094	Turkey_caecum_content	EUCAMP	0,5	1	>64	>4	>16	2	C. coli
2012	13-CA00095	Turkey_caecum_content	EUCAMP	1	2	>64	>4	>16	2	C. coli
2012	13-CA00097	Turkey_caecum_content	EUCAMP	0,25	2	32	>4	>16	2	C. coli
2012	13-CA00098	Turkey_caecum_content	EUCAMP	0,5	<=1	4	0,25	<=0,25	1	C. jejuni
2012	13-CA00100	Turkey_caecum_content	EUCAMP	0,5	1	64	>4	>16	1	C. coli
2012	13-CA00102	Turkey_caecum_content	EUCAMP	0,5	4	64	>4	>16	1	C. coli
2012	13-CA00103	Turkey_caecum_content	EUCAMP	1	2	4	0,120	8	<=0,5	C. jejuni
2012	13-CA00104	Turkey_caecum_content	EUCAMP	<=0,125	<=1	16	2	8	<=0,5	C. jejuni
2012	13-CA00107	Turkey_caecum_content	EUCAMP	0,25	>16	32	>4	>16	>32	C. coli
2012	13-CA00108	Turkey_caecum_content	EUCAMP	0,5	2	>64	>4	1	2	C. jejuni
2012	13-CA00151	Turkey_caecum_content	EUCAMP	0,5	2	64	>4	>16	<=0,5	C. coli
2013	13-CA00168	Broiler_caecum_content	EUCAMP	0,5	2	>64	>4	>16	2	C. coli
2013	13-CA00246	Broiler_caecum_content	EUCAMP	0,5	1	64	>4	<=0,25	<=0,5	C. jejuni
2013	13-CA00288	Broiler_caecum_content	EUCAMP	1	4	4	0,120	<=0,25	<=0,5	C. jejuni
2013	13-CA00289	Broiler_caecum_content	EUCAMP	0,5	2	64	>4	16	<=0,5	C. coli
2013	13-CA00297	Broiler_caecum_content	EUCAMP	0,5	2	4	0,120	<=0,25	1	C. jejuni
2013	13-CA00300	Broiler_caecum_content	EUCAMP	0,25	1	64	>4	<=0,25	2	C. jejuni
2013	13-CA00303	Broiler_caecum_content	EUCAMP	0,5	2	4	0,120	<=0,25	2	C. jejuni
2013	13-CA00351	Broiler_caecum_content	EUCAMP	0,25	1	4	0,120	<=0,25	<=0,5	C. jejuni
2013	13-CA00454	Broiler_caecum_content	EUCAMP	0,25	1	32	>4	16	<=0,5	C. jejuni
2013	13-CA00477	Broiler_caecum_content	EUCAMP	0,5	2	>64	>4	>16	1	C. jejuni
2013	13-CA00483	Broiler_caecum_content	EUCAMP	0,25	1	4	0,25	<=0,25	<=0,5	C. jejuni
2013	13-CA00531	Broiler_caecum_content	EUCAMP	0,5	2	64	>4	>16	2	C. coli
2013	13-CA00532	Broiler_caecum_content	EUCAMP	0,25	1	32	>4	16	4	C. coli

Table S5: Minimum inhibitory concentrations of 6 antimicrobials for isolats of C. jejuni and C. coli from poultry

Year	Sample No.	Matrix_final	Antimicrobial panel	GEN	STR	NAL	CIP	TET	ERY	Species
2013	13-CA00535	Broiler_caecum_content	EUCAMP	1	4	8	0,25	<=0,25	2	C. jejuni
2013	13-CA00537	Broiler_caecum_content	EUCAMP	0,25	1	32	>4	>16	<=0,5	C. jejuni
2013	13-CA00574	Broiler_caecum_content	EUCAMP	0,5	2	4	0,120	<=0,25	1	C. jejuni
2013	13-CA00578	Broiler_caecum_content	EUCAMP	0,25	1	>64	>4	>16	2	C. jejuni
2013	13-CA00581	Broiler_caecum_content	EUCAMP	0,5	2	>64	>4	>16	2	C. coli
2013	13-CA00582	Broiler_caecum_content	EUCAMP	0,5	2	8	0,5	>16	2	C. coli
2013	13-CA00626	Broiler_caecum_content	EUCAMP	0,5	1	64	>4	>16	<=0,5	C. jejuni
2013	13-CA00643	Broiler_caecum_content	EUCAMP	<=0,125	1	4	0,120	<=0,25	<=0,5	C. jejuni
2013	13-CA00718	Broiler_caecum_content	EUCAMP	0,25	1	64	>4	>16	1	C. coli
2013	13-CA00719	Broiler_caecum_content	EUCAMP	0,25	1	64	>4	>16	2	C. coli
2013	13-CA00721	Broiler_caecum_content	EUCAMP	0,5	1	4	0,25	<=0,25	1	C. coli
2013	13-CA00722	Broiler_caecum_content	EUCAMP	0,25	1	4	0,120	<=0,25	<=0,5	C. jejuni
2013	13-CA00723	Broiler_caecum_content	EUCAMP	0,5	1	>64	>4	<=0,25	<=0,5	C. jejuni
2013	13-CA00734	Broiler_caecum_content	EUCAMP	<=0,125	1	64	>4	>16	<=0,5	C. jejuni
2013	13-CA00744	Broiler_caecum_content	EUCAMP	0,5	2	<=2	0,120	<=0,25	1	C. jejuni
2013	13-CA00745	Broiler_caecum_content	EUCAMP	0,5	2	64	>4	>16	<=0,5	C. coli
2013	13-CA00756	Broiler_caecum_content	EUCAMP	<=0,125	1	8	<=0,06	<=0,25	<=0,5	C. jejuni
2013	13-CA00757	Broiler_caecum_content	EUCAMP	0,5	1	64	>4	<=0,25	2	C. coli
2013	13-CA00759	Broiler_caecum_content	EUCAMP	0,5	1	>64	>4	<=0,25	1	C. jejuni
2013	13-CA00760	Broiler_caecum_content	EUCAMP	0,25	1	32	>4	>16	<=0,5	C. jejuni
2013	13-CA00773	Broiler_caecum_content	EUCAMP	0,5	2	4	0,120	<=0,25	2	C. jejuni
2013	13-CA00775	Broiler_caecum_content	EUCAMP	0,5	1	4	0,120	<=0,25	<=0,5	C. jejuni
2013	13-CA00782	Broiler_caecum_content	EUCAMP	<=0,125	1	32	>4	<=0,25	<=0,5	C. jejuni
2013	13-CA00800	Broiler_caecum_content	EUCAMP	0,5	2	>64	>4	<=0,25	1	C. jejuni
2013	13-CA00805	Broiler_caecum_content	EUCAMP	<=0,125	1	4	0,120	<=0,25	<=0,5	C. jejuni
2013	13-CA00807	Broiler_caecum_content	EUCAMP	0,5	>16	64	>4	>16	2	C. coli
2013	13-CA00851	Broiler_caecum_content	EUCAMP	0,25	1	64	>4	>16	2	C. jejuni
2013	13-CA00910	Broiler_caecum_content	EUCAMP	0,5	1	>64	>4	>16	<=0,5	C. jejuni
2013	13-CA00912	Broiler_caecum_content	EUCAMP	<=0,125	>16	4	0,5	<=0,25	<=0,5	C. jejuni
2013	13-CA00916	Broiler_caecum_content	EUCAMP	0,25	1	<=2	0,120	<=0,25	1	C. jejuni
2013	13-CA01047	Broiler_caecum_content	EUCAMP	0,5	2	<=2	>4	<=0,25	1	C. jejuni
2013	13-CA01049	Broiler_caecum_content	EUCAMP	0,5	1	<=2	>4	<=0,25	<=0,5	C. jejuni

Table S5: Minimum inhibitory concentrations of 6 antimicrobials for isolats of C. jejuni and C. coli from poultry

Year	Sample No.	Matrix_final	Antimicrobial panel	GEN	STR	NAL	CIP	TET	ERY	Species
2013	13-CA01092	Broiler_caecum_content	EUCAMP	0,5	2	64	>4	<=0,25	<=0,5	C. coli
2013	13-CA01188	Broiler_caecum_content	EUCAMP	<=0,125	1	32	>4	16	1	C. jejuni
2013	13-CA01284	Broiler_caecum_content	EUCAMP	0,5	2	<=2	0,120	>16	1	C. jejuni
2013	13-CA01285	Broiler_caecum_content	EUCAMP	0,5	2	64	>4	<=0,25	2	C. coli
2013	13-CA01286	Broiler_caecum_content	EUCAMP	0,5	2	<=2	0,120	>16	1	C. jejuni
2013	13-CA01287	Broiler_caecum_content	EUCAMP	0,5	2	64	>4	>16	2	C. coli
2013	13-CA01288	Broiler_caecum_content	EUCAMP	0,5	2	4	0,120	<=0,25	<=0,5	C. jejuni
2013	13-CA01290	Broiler_caecum_content	EUCAMP	0,5	2	4	0,120	<=0,25	1	C. jejuni
2013	13-CA01294	Broiler_caecum_content	EUCAMP	0,5	2	8	0,5	<=0,25	<=0,5	C. coli
2013	13-CA01295	Broiler_caecum_content	EUCAMP	0,5	1	4	0,120	>16	<=0,5	C. jejuni
2013	14-CA00200	Broiler_caecum_content	EUCAMP	0,25	1	32	>4	<=0,25	<=0,5	C. jejuni
2014	14-CA00105	Broiler_caecum_content	EUCAMP2	0,5	2	8	0,25	<=0,5	2	C. coli
2014	14-CA00152	Broiler_caecum_content	EUCAMP2	<=0,125	<=0,25	16	8	<=0,5	<=1	C. jejuni
2014	14-CA00154	Broiler_caecum_content	EUCAMP2	0,5	1	4	0,25	<=0,5	2	C. coli
2014	14-CA00238	Broiler_caecum_content	EUCAMP2	0,5	2	8	0,25	>64	<=1	C. coli
2014	14-CA00245	Broiler_caecum_content	EUCAMP2	0,5	1	32	16	64	<=1	C. jejuni
2014	14-CA00246	Broiler_caecum_content	EUCAMP2	0,5	1	64	8	<=0,5	<=1	C. coli
2014	14-CA00247	Turkey_caecum_content	EUCAMP2	0,25	2	64	8	<=0,5	<=1	C. coli
2014	14-CA00248	Turkey_caecum_content	EUCAMP2	0,25	2	64	>16	>64	2	C. coli
2014	14-CA00248	Turkey_caecum_content	EUCAMP2	0,25	0,5	8	0,5	>64	<=1	C. jejuni
2014	14-CA00249	Turkey_caecum_content	EUCAMP2	0,5	2	4	0,25	<=0,5	<=1	C. coli
2014	14-CA00250	Broiler_caecum_content	EUCAMP2	0,5	2	64	8	<=0,5	<=1	C. coli
2014	14-CA00259	Turkey_caecum_content	EUCAMP2	<=0,125	<=0,25	64	16	64	2	C. jejuni
2014	14-CA00271	Turkey_caecum_content	EUCAMP2	0,5	1	64	16	<=0,5	<=1	C. jejuni
2014	14-CA00272	Turkey_caecum_content	EUCAMP2	0,5	1	8	1	32	<=1	C. jejuni
2014	14-CA00273	Turkey_caecum_content	EUCAMP2	0,5	1	8	0,5	<=0,5	<=1	C. jejuni
2014	14-CA00283	Broiler_caecum_content	EUCAMP2	<=0,125	<=0,25	4	<=0,125	<=0,5	<=1	C. jejuni
2014	14-CA00284	Turkey_caecum_content	EUCAMP2	0,25	>16	16	8	32	<=1	C. coli
2014	14-CA00285	Turkey_caecum_content	EUCAMP2	0,25	0,5	64	>16	<=0,5	<=1	C. jejuni
2014	14-CA00286	Turkey_caecum_content	EUCAMP2	0,5	2	64	>16	>64	2	C. coli
2014	14-CA00287	Broiler_caecum_content	EUCAMP2	0,5	0,5	2	<=0,125	<=0,5	<=1	C. jejuni
2014	14-CA00297	Turkey_caecum_content	EUCAMP2	0,5	2	64	>16	>64	<=1	C. coli

Table S5: Minimum inhibitory concentrations of 6 antimicrobials for isolats of C. jejuni and C. coli from poultry

Year	Sample No.	Matrix_final	Antimicrobial panel	GEN	STR	NAL	CIP	TET	ERY	Species
2014	14-CA00302	Turkey_caecum_content	EUCAMP2	0,5	1	2	<=0,125	<=0,5	<=1	C. jejuni
2014	14-CA00303	Turkey_caecum_content	EUCAMP2	0,25	0,5	8	0,5	64	<=1	C. jejuni
2014	14-CA00304	Turkey_caecum_content	EUCAMP2	<=0,125	<=0,25	32	16	64	<=1	C. jejuni
2014	14-CA00305	Turkey_caecum_content	EUCAMP2	0,5	1	64	16	64	<=1	C. coli
2014	14-CA00325	Broiler_caecum_content	EUCAMP2	1	2	>64	16	<=0,5	<=1	C. jejuni
2014	14-CA00326	Broiler_caecum_content	EUCAMP2	<=0,125	<=0,25	4	<=0,125	<=0,5	<=1	C. jejuni
2014	14-CA00333	Broiler_caecum_content	EUCAMP2	0,5	2	64	16	<=0,5	<=1	C. coli
2014	14-CA00334	Broiler_caecum_content	EUCAMP2	0,5	1	64	16	64	<=1	C. jejuni
2014	14-CA00335	Broiler_caecum_content	EUCAMP2	1	4	64	16	64	<=1	C. coli
2014	14-CA00336	Broiler_caecum_content	EUCAMP2	<=0,125	<=0,25	16	8	<=0,5	<=1	C. jejuni
2014	14-CA00337	Broiler_caecum_content	EUCAMP2	0,25	<=0,25	16	8	<=0,5	<=1	C. jejuni
2014	14-CA00338	Broiler_caecum_content	EUCAMP2	0,5	1	64	>16	32	<=1	C. coli
2014	14-CA00344	Broiler_caecum_content	EUCAMP2	0,5	2	>64	16	32	<=1	C. coli
2014	14-CA00352	Turkey_caecum_content	EUCAMP2	1	1	64	>16	>64	>128	C. coli
2014	14-CA00353	Turkey_caecum_content	EUCAMP2	<=0,125	<=0,25	32	16	64	<=1	C. jejuni
2014	14-CA00354	Turkey_caecum_content	EUCAMP2	0,25	1	4	<=0,125	<=0,5	<=1	C. jejuni
2014	14-CA00355	Turkey_caecum_content	EUCAMP2	0,25	0,5	32	>16	>64	<=1	C. jejuni
2014	14-CA00356	Turkey_caecum_content	EUCAMP2	0,25	1	>64	>16	>64	2	C. jejuni
2014	14-CA00357	Turkey_caecum_content	EUCAMP2	0,5	>16	>64	>16	>64	>128	C. coli
2014	14-CA00358	Broiler_caecum_content	EUCAMP2	0,5	1	4	0,25	<=0,5	<=1	C. coli
2014	14-CA00359	Broiler_caecum_content	EUCAMP2	0,5	1	64	8	64	<=1	C. jejuni
2014	14-CA00360	Turkey_caecum_content	EUCAMP2	0,5	2	64	>16	>64	2	C. coli
2014	14-CA00361	Turkey_caecum_content	EUCAMP2	0,5	0,5	4	0,25	<=0,5	<=1	C. coli
2014	14-CA00362	Turkey_caecum_content	EUCAMP2	0,5	1	>64	16	>64	<=1	C. jejuni
2014	14-CA00363	Broiler_caecum_content	EUCAMP2	0,5	2	>64	>16	64	<=1	C. jejuni
2014	14-CA00364	Broiler_caecum_content	EUCAMP2	0,5	1	64	>16	64	>128	C. coli
2014	14-CA00365	Broiler_caecum_content	EUCAMP2	0,25	0,5	64	16	32	<=1	C. jejuni
2014	14-CA00366	Turkey_caecum_content	EUCAMP2	0,5	2	64	16	<=0,5	<=1	C. coli
2014	14-CA00367	Turkey_caecum_content	EUCAMP2	0,5	2	64	8	32	<=1	C. coli
2014	14-CA00368	Turkey_caecum_content	EUCAMP2	0,5	2	64	>16	64	>128	C. coli
2014	14-CA00369	Turkey_caecum_content	EUCAMP2	0,5	1	64	16	>64	<=1	C. coli
2014	14-CA00370	Turkey_caecum_content	EUCAMP2	0,5	1	>64	>16	>64	>128	C. coli

Table S5: Minimum inhibitory concentrations of 6 antimicrobials for isolats of C. jejuni and C. coli from poultry

Year	Sample No.	Matrix_final	Antimicrobial panel	GEN	STR	NAL	CIP	TET	ERY	Species
2014	14-CA00371	Turkey_caecum_content	EUCAMP2	0,5	1	64	16	64	<=1	C. jejuni
2014	14-CA00372	Turkey_caecum_content	EUCAMP2	0,5	2	64	>16	64	>128	C. coli
2014	14-CA00373	Turkey_caecum_content	EUCAMP2	0,5	2	64	>16	>64	>128	C. coli
2014	14-CA00374	Turkey_caecum_content	EUCAMP2	0,5	2	8	0,5	<=0,5	2	C. jejuni
2014	14-CA00375	Turkey_caecum_content	EUCAMP2	0,25	1	4	<=0,125	<=0,5	<=1	C. jejuni
2014	14-CA00376	Turkey_caecum_content	EUCAMP2	0,5	2	>64	>16	64	2	C. coli
2014	14-CA00377	Turkey_caecum_content	EUCAMP2	0,25	>16	32	16	32	<=1	C. coli
2014	14-CA00378	Turkey_caecum_content	EUCAMP2	<=0,125	0,5	4	<=0,125	<=0,5	<=1	C. coli
2014	14-CA00379	Turkey_caecum_content	EUCAMP2	0,5	2	64	16	<=0,5	<=1	C. jejuni
2014	14-CA00380	Turkey_caecum_content	EUCAMP2	0,5	2	>64	>16	>64	>128	C. coli
2014	14-CA00381	Broiler_caecum_content	EUCAMP2	0,5	1	>64	>16	>64	>128	C. jejuni
2014	14-CA00382	Turkey_caecum_content	EUCAMP2	<=0,125	<=0,25	64	16	16	<=1	C. jejuni
2014	14-CA00383	Turkey_caecum_content	EUCAMP2	1	2	>64	16	<=0,5	<=1	C. jejuni
2014	14-CA00384	Turkey_caecum_content	EUCAMP2	0,5	1	4	<=0,125	<=0,5	<=1	C. jejuni
2014	14-CA00385	Turkey_caecum_content	EUCAMP2	0,5	2	4	<=0,125	>64	<=1	C. jejuni
2014	14-CA00386	Turkey_caecum_content	EUCAMP2	0,5	1	8	1	>64	2	C. jejuni
2014	14-CA00387	Turkey_caecum_content	EUCAMP2	0,5	2	4	0,25	<=0,5	<=1	C. jejuni
2014	14-CA00388	Turkey_caecum_content	EUCAMP2	1	2	8	0,25	64	<=1	C. coli
2014	14-CA00389	Turkey_caecum_content	EUCAMP2	0,5	1	>64	>16	>64	2	C. jejuni
2014	14-CA00390	Turkey_caecum_content	EUCAMP2	1	1	4	<=0,125	64	<=1	C. jejuni
2014	14-CA00391	Turkey_caecum_content	EUCAMP2	0,5	1	64	>16	>64	2	C. jejuni
2014	14-CA00392	Turkey_caecum_content	EUCAMP2	0,25	2	64	>16	>64	<=1	C. coli
2014	14-CA00392	Turkey_caecum_content	EUCAMP2	0,25	0,5	32	16	64	<=1	C. jejuni
2014	14-CA00393	Turkey_caecum_content	EUCAMP2	1	2	>64	16	<=0,5	<=1	C. jejuni
2014	14-CA00394	Turkey_caecum_content	EUCAMP2	<=0,125	0,5	16	4	8	<=1	C. jejuni
2014	14-CA00395	Turkey_caecum_content	EUCAMP2	0,5	2	64	>16	64	>128	C. coli
2014	14-CA00396	Turkey_caecum_content	EUCAMP2	0,5	>16	>64	>16	>64	>128	C. coli
2014	14-CA00397	Turkey_caecum_content	EUCAMP2	0,25	1	4	<=0,125	<=0,5	<=1	C. jejuni
2014	14-CA00398	Turkey_caecum_content	EUCAMP2	0,5	2	64	16	64	<=1	C. coli
2014	14-CA00399	Turkey_caecum_content	EUCAMP2	<=0,125	0,5	64	>16	>64	<=1	C. jejuni
2014	14-CA00400	Turkey_caecum_content	EUCAMP2	0,5	2	>64	>16	64	>128	C. coli
2014	14-CA00401	Turkey_caecum_content	EUCAMP2	0,5	2	64	16	>64	<=1	C. coli

Table S5: Minimum inhibitory concentrations of 6 antimicrobials for isolats of C. jejuni and C. coli from poultry

Year	Sample No.	Matrix_final	Antimicrobial panel	GEN	STR	NAL	CIP	TET	ERY	Species
2014	14-CA00402	Turkey_caecum_content	EUCAMP2	0,5	2	64	>16	>64	2	C. coli
2014	14-CA00403	Turkey_caecum_content	EUCAMP2	0,25	1	64	16	16	<=1	C. jejuni
2014	14-CA00404	Broiler_caecum_content	EUCAMP2	0,25	0,5	4	0,25	<=0,5	<=1	C. jejuni
2014	14-CA00405	Turkey_caecum_content	EUCAMP2	0,5	2	64	>16	64	>128	C. coli
2014	14-CA00406	Turkey_caecum_content	EUCAMP2	0,5	1	64	>16	32	<=1	C. jejuni
2014	14-CA00407	Turkey_caecum_content	EUCAMP2	0,25	0,5	64	>16	<=0,5	<=1	C. jejuni
2014	14-CA00408	Turkey_caecum_content	EUCAMP2	0,5	1	64	8	64	128	C. coli
2014	14-CA00409	Turkey_caecum_content	EUCAMP2	1	4	>64	>16	>64	<=1	C. coli
2014	14-CA00410	Turkey_caecum_content	EUCAMP2	0,5	1	4	<=0,125	<=0,5	<=1	C. jejuni
2014	14-CA00411	Turkey_caecum_content	EUCAMP2	0,5	>16	4	>16	>64	<=1	C. jejuni
2014	14-CA00412	Turkey_caecum_content	EUCAMP2	2	>16	16	0,25	64	2	C. coli
2014	14-CA00413	Turkey_caecum_content	EUCAMP2	0,5	1	8	0,5	16	<=1	C. jejuni
2014	14-CA00416	Broiler_caecum_content	EUCAMP2	0,5	1	>64	>16	>64	2	C. jejuni
2014	14-CA00418	Broiler_caecum_content	EUCAMP2	0,25	0,5	16	8	32	<=1	C. jejuni
2014	14-CA00427	Broiler_caecum_content	EUCAMP2	0,5	1	64	16	64	<=1	C. coli
2014	14-CA00429	Turkey_caecum_content	EUCAMP2	0,5	>16	>64	>16	64	<=1	C. coli
2014	14-CA00430	Broiler_caecum_content	EUCAMP2	0,25	0,5	64	>16	>64	>128	C. jejuni
2014	14-CA00431	Broiler_caecum_content	EUCAMP2	0,25	0,5	64	>16	>64	>128	C. jejuni
2014	14-CA00432	Turkey_caecum_content	EUCAMP2	0,5	0,5	64	16	<=0,5	<=1	C. jejuni
2014	14-CA00438	Broiler_caecum_content	EUCAMP2	0,25	1	32	16	<=0,5	<=1	C. jejuni
2014	14-CA00439	Broiler_caecum_content	EUCAMP2	0,5	1	>64	16	32	<=1	C. coli
2014	14-CA00440	Turkey_caecum_content	EUCAMP2	0,5	1	>64	>16	>64	<=1	C. jejuni
2014	14-CA00441	Turkey_caecum_content	EUCAMP2	<=0,125	<=0,25	32	4	16	<=1	C. jejuni
2014	14-CA00442	Turkey_caecum_content	EUCAMP2	0,5	0,5	32	8	32	<=1	C. jejuni
2014	14-CA00444	Broiler_caecum_content	EUCAMP2	0,25	0,5	16	4	<=0,5	<=1	C. jejuni
2014	14-CA00452	Turkey_caecum_content	EUCAMP2	0,5	1	>64	>16	>64	<=1	C. jejuni
2014	14-CA00453	Broiler_caecum_content	EUCAMP2	0,25	0,5	32	8	<=0,5	<=1	C. jejuni
2014	14-CA00456	Turkey_caecum_content	EUCAMP2	0,25	1	64	>16	>64	<=1	C. jejuni
2014	14-CA00457	Turkey_caecum_content	EUCAMP2	0,5	1	>64	>16	>64	<=1	C. coli
2014	14-CA00458	Turkey_caecum_content	EUCAMP2	<=0,125	0,5	32	16	64	<=1	C. jejuni
2014	14-CA00459	Turkey_caecum_content	EUCAMP2	0,5	2	64	>16	64	>128	C. coli
2014	14-CA00460	Turkey_caecum_content	EUCAMP2	0,25	0,5	4	<=0,125	<=0,5	<=1	C. jejuni

Table S5: Minimum inhibitory concentrations of 6 antimicrobials for isolats of C. jejuni and C. coli from poultry

Year	Sample No.	Matrix_final	Antimicrobial panel	GEN	STR	NAL	CIP	TET	ERY	Species
2014	14-CA00461	Turkey_caecum_content	EUCAMP2	0,25	0,5	32	16	64	<=1	C. jejuni
2014	14-CA00462	Turkey_caecum_content	EUCAMP2	0,5	2	64	16	4	<=1	C. jejuni
2014	14-CA00463	Turkey_caecum_content	EUCAMP2	0,5	2	>64	>16	>64	>128	C. coli
2014	14-CA00464	Turkey_caecum_content	EUCAMP2	0,5	2	8	0,5	>64	<=1	C. coli
2014	14-CA00465	Turkey_caecum_content	EUCAMP2	0,5	>16	64	16	64	<=1	C. coli
2014	14-CA00466	Turkey_caecum_content	EUCAMP2	0,5	2	>64	>16	<=0,5	4	C. coli
2014	14-CA00467	Turkey_caecum_content	EUCAMP2	0,5	2	64	>16	>64	>128	C. coli
2014	14-CA00468	Turkey_caecum_content	EUCAMP2	0,5	1	64	16	>64	>128	C. coli
2014	14-CA00469	Broiler_caecum_content	EUCAMP2	0,5	2	>64	>16	1	<=1	C. jejuni
2014	14-CA00470	Turkey_caecum_content	EUCAMP2	1	4	>64	>16	>64	2	C. coli
2014	14-CA00471	Turkey_caecum_content	EUCAMP2	1	1	4	0,25	<=0,5	<=1	C. jejuni
2014	14-CA00472	Turkey_caecum_content	EUCAMP2	0,5	2	64	>16	64	>128	C. coli
2014	14-CA00473	Turkey_caecum_content	EUCAMP2	0,25	0,5	32	8	32	<=1	C. jejuni
2014	14-CA00474	Turkey_caecum_content	EUCAMP2	0,5	2	32	8	64	64	C. coli
2014	14-CA00475	Turkey_caecum_content	EUCAMP2	0,25	0,5	8	0,5	>64	<=1	C. jejuni
2014	14-CA00476	Turkey_caecum_content	EUCAMP2	1	>16	>64	>16	64	2	C. coli
2014	14-CA00477	Turkey_caecum_content	EUCAMP2	0,25	0,5	64	>16	>64	<=1	C. jejuni
2014	14-CA00478	Turkey_caecum_content	EUCAMP2	0,5	1	64	16	64	<=1	C. jejuni
2014	14-CA00479	Turkey_caecum_content	EUCAMP2	0,25	1	64	>16	<=0,5	2	C. jejuni
2014	14-CA00480	Turkey_caecum_content	EUCAMP2	0,25	1	16	16	32	<=1	C. jejuni
2014	14-CA00481	Broiler_caecum_content	EUCAMP2	0,5	1	4	0,25	<=0,5	<=1	C. jejuni
2014	14-CA00482	Broiler_caecum_content	EUCAMP2	0,25	0,5	4	<=0,125	<=0,5	<=1	C. jejuni
2014	14-CA00483	Turkey_caecum_content	EUCAMP2	0,5	>16	4	<=0,125	64	>128	C. coli
2014	14-CA00484	Turkey_caecum_content	EUCAMP2	0,25	>16	32	16	64	<=1	C. jejuni
2014	14-CA00485	Turkey_caecum_content	EUCAMP2	0,5	1	64	>16	>64	<=1	C. jejuni
2014	14-CA00486	Turkey_caecum_content	EUCAMP2	0,5	2	64	>16	64	2	C. coli
2014	14-CA00487	Turkey_caecum_content	EUCAMP2	1	4	>64	16	64	<=1	C. coli
2014	14-CA00488	Turkey_caecum_content	EUCAMP2	0,25	<=0,25	64	16	<=0,5	<=1	C. jejuni
2014	14-CA00489	Turkey_caecum_content	EUCAMP2	0,25	2	64	16	64	>128	C. coli
2014	14-CA00490	Turkey_caecum_content	EUCAMP2	0,5	2	64	>16	64	>128	C. coli
2014	14-CA00491	Turkey_caecum_content	EUCAMP2	0,5	1	64	>16	32	2	C. jejuni
2014	14-CA00492	Turkey_caecum_content	EUCAMP2	0,5	1	64	16	<=0,5	<=1	C. jejuni

Table S5: Minimum inhibitory concentrations of 6 antimicrobials for isolats of C. jejuni and C. coli from poultry

Year	Sample No.	Matrix_final	Antimicrobial panel	GEN	STR	NAL	CIP	TET	ERY	Species
2014	14-CA00493	Broiler_caecum_content	EUCAMP2	0,5	1	4	<=0,125	<=0,5	<=1	C. jejuni
2014	14-CA00516	Broiler_caecum_content	EUCAMP2	0,25	0,5	64	16	<=0,5	<=1	C. jejuni
2014	14-CA00517	Broiler_caecum_content	EUCAMP2	1	2	4	<=0,125	64	<=1	C. coli
2014	14-CA00518	Broiler_caecum_content	EUCAMP2	<=0,125	0,5	16	8	32	<=1	C. jejuni
2014	14-CA00519	Broiler_caecum_content	EUCAMP2	0,25	1	4	<=0,125	<=0,5	<=1	C. jejuni
2014	14-CA00520	Broiler_caecum_content	EUCAMP2	0,25	1	16	16	<=0,5	<=1	C. jejuni
2014	14-CA00521	Broiler_caecum_content	EUCAMP2	0,5	0,5	>64	>16	1	<=1	C. jejuni
2014	14-CA00522	Broiler_caecum_content	EUCAMP2	1	2	4	<=0,125	64	<=1	C. coli
2014	14-CA00523	Turkey_caecum_content	EUCAMP2	0,5	2	>64	16	<=0,5	<=1	C. jejuni
2014	14-CA00524	Broiler_caecum_content	EUCAMP2	0,5	2	64	16	<=0,5	<=1	C. jejuni
2014	14-CA00557	Broiler_caecum_content	EUCAMP2	0,5	1	32	16	<=0,5	<=1	C. jejuni
2014	14-CA00567	Broiler_caecum_content	EUCAMP2	0,25	1	4	0,25	<=0,5	<=1	C. jejuni
2014	14-CA00568	Broiler_caecum_content	EUCAMP2	0,25	1	16	4	32	64	C. coli
2014	14-CA00569	Broiler_caecum_content	EUCAMP2	<=0,125	<=0,25	4	0,5	64	<=1	C. jejuni
2014	14-CA00574	Turkey_caecum_content	EUCAMP2	0,5	1	32	>16	>64	>128	C. coli
2014	14-CA00575	Turkey_caecum_content	EUCAMP2	0,5	2	>64	>16	>64	<=1	C. coli
2014	14-CA00577	Turkey_caecum_content	EUCAMP2	0,5	1	64	>16	>64	<=1	C. jejuni
2014	14-CA00580	Turkey_caecum_content	EUCAMP2	0,5	>16	4	0,25	<=0,5	4	C. coli
2014	14-CA00581	Turkey_caecum_content	EUCAMP2	<=0,125	2	>64	>16	>64	2	C. coli
2014	14-CA00584	Turkey_caecum_content	EUCAMP2	0,5	2	64	>16	64	<=1	C. coli
2014	14-CA00585	Turkey_caecum_content	EUCAMP2	0,25	1	4	0,25	64	<=1	C. coli
2014	14-CA00586	Turkey_caecum_content	EUCAMP2	0,5	1	>64	>16	>64	<=1	C. coli
2014	14-CA00587	Turkey_caecum_content	EUCAMP2	0,5	1	4	<=0,125	<=0,5	<=1	C. jejuni
2014	14-CA00588	Turkey_caecum_content	EUCAMP2	0,5	1	>64	>16	>64	<=1	C. coli
2014	14-CA00590	Turkey_caecum_content	EUCAMP2	1	2	>64	16	>64	<=1	C. jejuni
2014	14-CA00592	Turkey_caecum_content	EUCAMP2	0,5	2	64	16	>64	<=1	C. coli
2014	14-CA00593	Turkey_caecum_content	EUCAMP2	1	2	8	0,25	<=0,5	<=1	C. jejuni
2014	14-CA00594	Turkey_caecum_content	EUCAMP2	0,5	2	4	<=0,125	<=0,5	<=1	C. jejuni
2014	14-CA00595	Turkey_caecum_content	EUCAMP2	0,5	2	64	>16	64	>128	C. coli
2014	14-CA00596	Turkey_caecum_content	EUCAMP2	1	2	8	0,25	64	<=1	C. coli
2014	14-CA00598	Turkey_caecum_content	EUCAMP2	0,5	2	>64	>16	>64	2	C. coli
2014	14-CA00607	Turkey_caecum_content	EUCAMP2	0,5	2	>64	>16	>64	2	C. jejuni

Table S5: Minimum inhibitory concentrations of 6 antimicrobials for isolats of C. jejuni and C. coli from poultry

Year	Sample No.	Matrix_final	Antimicrobial panel	GEN	STR	NAL	CIP	TET	ERY	Species
2014	14-CA00631	Broiler_caecum_content	EUCAMP2	0,25	1	64	8	<=0,5	<=1	C. jejuni
2014	14-CA00632	Broiler_caecum_content	EUCAMP2	0,25	2	>64	>16	64	<=1	C. coli
2014	14-CA00635	Broiler_caecum_content	EUCAMP2	0,25	1	2	16	<=0,5	<=1	C. jejuni
2014	14-CA00636	Broiler_caecum_content	EUCAMP2	0,5	1	>64	16	<=0,5	<=1	C. jejuni
2014	14-CA00637	Broiler_caecum_content	EUCAMP2	0,5	2	8	0,25	>64	<=1	C. coli
2014	14-CA00683	Broiler_caecum_content	EUCAMP2	0,5	>16	64	>16	>64	>128	C. coli
2014	14-CA00684	Broiler_caecum_content	EUCAMP2	0,5	2	8	0,25	<=0,5	<=1	C. jejuni
2014	14-CA00685	Broiler_caecum_content	EUCAMP2	<=0,125	<=0,25	32	8	16	<=1	C. jejuni
2014	14-CA00686	Broiler_caecum_content	EUCAMP2	0,5	0,5	64	16	<=0,5	<=1	C. jejuni
2014	14-CA00696	Broiler_caecum_content	EUCAMP2	0,5	1	64	16	<=0,5	<=1	C. jejuni
2014	14-CA00697	Turkey_caecum_content	EUCAMP2	0,5	2	>64	>16	>64	4	C. coli
2014	14-CA00698	Turkey_caecum_content	EUCAMP2	<=0,125	0,5	32	16	32	<=1	C. coli
2014	14-CA00699	Turkey_caecum_content	EUCAMP2	0,5	2	64	>16	64	2	C. coli
2014	14-CA00700	Broiler_caecum_content	EUCAMP2	0,5	2	64	>16	2	4	C. jejuni
2014	14-CA00701	Broiler_caecum_content	EUCAMP2	0,25	1	>64	>16	>64	<=1	C. jejuni
2014	14-CA00702	Broiler_caecum_content	EUCAMP2	0,5	2	>64	>16	64	2	C. jejuni
2014	14-CA00709	Broiler_caecum_content	EUCAMP2	0,5	1	64	16	64	<=1	C. jejuni
2014	14-CA00710	Broiler_caecum_content	EUCAMP2	0,5	2	4	0,25	<=0,5	<=1	C. jejuni
2014	14-CA00711	Broiler_caecum_content	EUCAMP2	0,5	1	>64	16	<=0,5	<=1	C. jejuni
2014	14-CA00712	Broiler_caecum_content	EUCAMP2	0,5	1	4	<=0,125	<=0,5	<=1	C. jejuni
2014	14-CA00713	Turkey_caecum_content	EUCAMP2	0,5	1	>64	>16	>64	2	C. jejuni
2014	14-CA00714	Turkey_caecum_content	EUCAMP2	0,25	1	>64	>16	>64	<=1	C. jejuni
2014	14-CA00715	Turkey_caecum_content	EUCAMP2	0,5	2	8	0,25	>64	<=1	C. jejuni
2014	14-CA00716	Turkey_caecum_content	EUCAMP2	0,5	1	8	0,25	>64	<=1	C. jejuni
2014	14-CA00717	Turkey_caecum_content	EUCAMP2	0,5	1	4	0,25	<=0,5	<=1	C. jejuni
2014	14-CA00718	Turkey_caecum_content	EUCAMP2	0,5	1	>64	>16	>64	<=1	C. jejuni
2014	14-CA00719	Broiler_caecum_content	EUCAMP2	0,25	1	>64	>16	>64	<=1	C. jejuni
2014	14-CA00720	Broiler_caecum_content	EUCAMP2	0,5	1	>64	>16	<=0,5	<=1	C. jejuni
2014	14-CA00721	Turkey_caecum_content	EUCAMP2	0,25	0,5	64	>16	>64	<=1	C. jejuni
2014	14-CA00722	Broiler_caecum_content	EUCAMP2	0,5	1	>64	>16	>64	<=1	C. jejuni
2014	14-CA00723	Turkey_caecum_content	EUCAMP2	0,25	0,5	16	1	<=0,5	<=1	C. jejuni
2014	14-CA00724	Turkey_caecum_content	EUCAMP2	0,5	1	64	16	64	>128	C. jejuni

Table S5: Minimum inhibitory concentrations of 6 antimicrobials for isolats of C. jejuni and C. coli from poultry

Year	Sample No.	Matrix_final	Antimicrobial panel	GEN	STR	NAL	CIP	TET	ERY	Species
2014	14-CA00725	Broiler_caecum_content	EUCAMP2	0,25	0,5	4	<=0,125	32	<=1	C. jejuni
2014	14-CA00726	Broiler_caecum_content	EUCAMP2	<=0,125	0,5	4	0,25	<=0,5	<=1	C. jejuni
2014	14-CA00727	Broiler_caecum_content	EUCAMP2	0,25	1	>64	>16	>64	<=1	C. jejuni
2014	14-CA00728	Broiler_caecum_content	EUCAMP2	1	>16	32	16	64	<=1	C. coli
2014	14-CA00729	Broiler_caecum_content	EUCAMP2	0,5	1	64	16	<=0,5	<=1	C. jejuni
2014	14-CA00730	Turkey_caecum_content	EUCAMP2	1	4	8	0,25	64	<=1	C. coli
2014	14-CA00731	Turkey_caecum_content	EUCAMP2	0,25	0,5	<=1	16	64	<=1	C. jejuni
2014	14-CA00732	Broiler_caecum_content	EUCAMP2	0,25	0,5	<=1	8	2	<=1	C. jejuni
2014	14-CA00733	Broiler_caecum_content	EUCAMP2	1	2	4	<=0,125	<=0,5	<=1	C. jejuni
2014	14-CA00734	Broiler_caecum_content	EUCAMP2	1	2	>64	16	64	<=1	C. jejuni
2014	14-CA00735	Broiler_caecum_content	EUCAMP2	0,5	2	64	>16	64	>128	C. coli
2014	14-CA00736	Turkey_caecum_content	EUCAMP2	0,5	2	8	0,25	64	<=1	C. coli
2014	14-CA00737	Turkey_caecum_content	EUCAMP2	0,5	1	>64	>16	>64	>128	C. coli
2014	14-CA00738	Turkey_caecum_content	EUCAMP2	0,25	1	16	8	<=0,5	<=1	C. coli
2014	14-CA00738	Turkey_caecum_content	EUCAMP2	0,5	2	64	>16	>64	<=1	C. jejuni
2014	14-CA00739	Turkey_caecum_content	EUCAMP2	1	>16	>64	16	4	2	C. jejuni
2014	14-CA00740	Turkey_caecum_content	EUCAMP2	1	>16	>64	16	64	<=1	C. jejuni
2014	14-CA00741	Turkey_caecum_content	EUCAMP2	0,5	1	32	16	16	<=1	C. jejuni
2014	14-CA00742	Turkey_caecum_content	EUCAMP2	>16	>16	>64	>16	>64	>128	C. coli
2014	14-CA00743	Broiler_caecum_content	EUCAMP2	0,5	2	>64	>16	>64	2	C. jejuni
2014	14-CA00744	Broiler_caecum_content	EUCAMP2	1	4	>64	16	>64	<=1	C. coli
2014	14-CA00745	Broiler_caecum_content	EUCAMP2	<=0,125	<=0,25	32	16	>64	<=1	C. jejuni
2014	14-CA00746	Broiler_caecum_content	EUCAMP2	0,5	2	>64	>16	>64	<=1	C. coli
2014	14-CA00747	Broiler_caecum_content	EUCAMP2	0,5	2	4	<=0,125	<=0,5	<=1	C. jejuni
2014	14-CA00748	Broiler_caecum_content	EUCAMP2	0,5	2	64	>16	64	<=1	C. coli
2014	14-CA00749	Turkey_caecum_content	EUCAMP2	1	>16	64	16	>64	>128	C. coli
2014	14-CA00750	Turkey_caecum_content	EUCAMP2	0,5	1	16	8	16	<=1	C. jejuni
2014	14-CA00751	Turkey_caecum_content	EUCAMP2	0,5	1	4	<=0,125	<=0,5	<=1	C. jejuni
2014	14-CA00752	Turkey_caecum_content	EUCAMP2	0,5	2	32	16	>64	>128	C. coli
2014	14-CA00753	Turkey_caecum_content	EUCAMP2	0,5	2	32	16	16	<=1	C. jejuni
2014	14-CA00754	Turkey_caecum_content	EUCAMP2	0,25	1	32	16	>64	<=1	C. coli
2014	14-CA00755	Turkey_caecum_content	EUCAMP2	0,5	2	64	>16	>64	2	C. coli

Table S5: Minimum inhibitory concentrations of 6 antimicrobials for isolats of C. jejuni and C. coli from poultry

Year	Sample No.	Matrix_final	Antimicrobial panel	GEN	STR	NAL	CIP	TET	ERY	Species
2014	14-CA00756	Turkey_caecum_content	EUCAMP2	0,5	1	4	<=0,125	<=0,5	<=1	C. jejuni
2014	14-CA00757	Turkey_caecum_content	EUCAMP2	0,5	2	32	16	32	>128	C. coli
2014	14-CA00758	Turkey_caecum_content	EUCAMP2	0,5	1	4	<=0,125	<=0,5	<=1	C. jejuni
2014	14-CA00759	Turkey_caecum_content	EUCAMP2	0,5	0,5	8	0,25	<=0,5	<=1	C. jejuni
2014	14-CA00760	Broiler_caecum_content	EUCAMP2	1	>16	64	16	>64	>128	C. coli
2014	14-CA00761	Broiler_caecum_content	EUCAMP2	0,25	<=0,25	8	0,25	32	<=1	C. jejuni
2014	14-CA00762	Broiler_caecum_content	EUCAMP2	<=0,125	<=0,25	32	16	64	<=1	C. jejuni
2014	14-CA00763	Broiler_caecum_content	EUCAMP2	0,25	<=0,25	64	16	32	<=1	C. jejuni
2014	14-CA00764	Broiler_caecum_content	EUCAMP2	0,25	0,5	64	16	32	<=1	C. coli
2014	14-CA00765	Broiler_caecum_content	EUCAMP2	0,5	1	64	16	<=0,5	<=1	C. jejuni
2014	14-CA00766	Broiler_caecum_content	EUCAMP2	<=0,125	0,5	16	4	<=0,5	<=1	C. jejuni
2014	14-CA00767	Turkey_caecum_content	EUCAMP2	0,25	1	32	>16	>64	<=1	C. jejuni
2014	14-CA00768	Turkey_caecum_content	EUCAMP2	0,5	2	64	16	16	<=1	C. jejuni
2014	14-CA00769	Turkey_caecum_content	EUCAMP2	0,5	0,5	2	0,5	>64	<=1	C. jejuni
2014	14-CA00770	Turkey_caecum_content	EUCAMP2	0,5	2	64	>16	>64	<=1	C. coli
2014	14-CA00771	Turkey_caecum_content	EUCAMP2	0,5	1	64	16	<=0,5	<=1	C. jejuni
2014	14-CA00772	Turkey_caecum_content	EUCAMP2	0,5	2	64	>16	>64	>128	C. coli
2014	14-CA00773	Turkey_caecum_content	EUCAMP2	0,5	1	4	0,25	<=0,5	<=1	C. jejuni
2014	14-CA00774	Turkey_caecum_content	EUCAMP2	<=0,125	1	2	<=0,125	16	<=1	C. jejuni
2014	14-CA00775	Turkey_caecum_content	EUCAMP2	0,25	1	4	<=0,125	<=0,5	<=1	C. jejuni
2014	14-CA00776	Broiler_caecum_content	EUCAMP2	0,5	1	64	>16	64	>128	C. coli
2014	14-CA00777	Broiler_caecum_content	EUCAMP2	0,5	2	>64	>16	<=0,5	<=1	C. coli
2014	14-CA00778	Broiler_caecum_content	EUCAMP2	0,25	1	32	16	64	<=1	C. coli
2014	14-CA00779	Broiler_caecum_content	EUCAMP2	1	1	8	0,25	<=0,5	<=1	C. jejuni
2014	14-CA00780	Broiler_caecum_content	EUCAMP2	0,25	1	64	>16	>64	<=1	C. jejuni
2014	14-CA00781	Broiler_caecum_content	EUCAMP2	0,5	1	64	>16	64	>128	C. coli
2014	14-CA00782	Broiler_caecum_content	EUCAMP2	<=0,125	0,5	32	>16	>64	<=1	C. jejuni
2014	14-CA00783	Broiler_caecum_content	EUCAMP2	0,5	1	32	>16	64	>128	C. coli
2014	14-CA00784	Broiler_caecum_content	EUCAMP2	0,25	0,5	4	0,25	8	<=1	C. jejuni
2014	14-CA00785	Turkey_caecum_content	EUCAMP2	0,25	0,5	4	0,25	64	<=1	C. jejuni
2014	14-CA00786	Turkey_caecum_content	EUCAMP2	0,25	0,5	4	<=0,125	64	<=1	C. jejuni
2014	14-CA00787	Turkey_caecum_content	EUCAMP2	0,25	1	16	0,5	1	<=1	C. jejuni

Table S5: Minimum inhibitory concentrations of 6 antimicrobials for isolats of C. jejuni and C. coli from poultry

Year	Sample No.	Matrix_final	Antimicrobial panel	GEN	STR	NAL	CIP	TET	ERY	Species
2014	14-CA00788	Turkey_caecum_content	EUCAMP2	<=0,125	0,5	4	<=0,125	<=0,5	<=1	C. jejuni
2014	14-CA00789	Broiler_caecum_content	EUCAMP2	1	2	>64	16	64	<=1	C. jejuni
2014	14-CA00790	Turkey_caecum_content	EUCAMP2	0,25	0,5	32	8	64	128	C. coli
2014	14-CA00791	Turkey_caecum_content	EUCAMP2	0,5	1	>64	>16	>64	<=1	C. coli
2014	14-CA00792	Turkey_caecum_content	EUCAMP2	0,25	2	64	>16	>64	>128	C. coli
2014	14-CA00793	Turkey_caecum_content	EUCAMP2	0,5	2	4	0,25	<=0,5	<=1	C. jejuni
2014	14-CA00794	Turkey_caecum_content	EUCAMP2	0,25	>16	<=1	0,25	64	<=1	C. jejuni
2014	14-CA00795	Turkey_caecum_content	EUCAMP2	0,5	2	64	16	32	<=1	C. coli
2014	14-CA00796	Turkey_caecum_content	EUCAMP2	0,5	2	64	16	64	<=1	C. coli
2014	14-CA00797	Turkey_caecum_content	EUCAMP2	0,5	2	64	8	<=0,5	<=1	C. coli
2014	14-CA00798	Broiler_caecum_content	EUCAMP2	0,5	1	>64	>16	>64	>128	C. coli
2014	14-CA00799	Broiler_caecum_content	EUCAMP2	0,5	2	4	<=0,125	<=0,5	<=1	C. jejuni
2014	14-CA00800	Broiler_caecum_content	EUCAMP2	0,25	0,5	64	>16	32	<=1	C. jejuni
2014	14-CA00801	Broiler_caecum_content	EUCAMP2	0,25	0,5	64	16	32	<=1	C. coli
2014	14-CA00802	Broiler_caecum_content	EUCAMP2	0,5	2	64	>16	64	2	C. coli
2014	14-CA00803	Turkey_caecum_content	EUCAMP2	0,25	1	4	0,25	32	<=1	C. coli
2014	14-CA00804	Turkey_caecum_content	EUCAMP2	1	4	8	<=0,125	>64	<=1	C. coli
2014	14-CA00805	Turkey_caecum_content	EUCAMP2	0,5	2	>64	>16	>64	>128	C. coli
2014	14-CA00806	Broiler_caecum_content	EUCAMP2	0,5	1	4	0,25	>64	<=1	C. jejuni
2014	14-CA00807	Broiler_caecum_content	EUCAMP2	0,5	2	4	0,25	64	<=1	C. jejuni
2014	14-CA00808	Broiler_caecum_content	EUCAMP2	0,25	0,5	4	0,25	64	<=1	C. jejuni
2014	14-CA00809	Broiler_caecum_content	EUCAMP2	0,5	1	4	0,25	>64	<=1	C. jejuni
2014	14-CA00810	Broiler_caecum_content	EUCAMP2	0,25	0,5	4	0,25	64	<=1	C. jejuni
2014	14-CA00811	Broiler_caecum_content	EUCAMP2	0,5	2	4	<=0,125	<=0,5	<=1	C. jejuni
2014	14-CA00812	Broiler_caecum_content	EUCAMP2	0,5	2	>64	>16	>64	<=1	C. coli
2014	14-CA00814	Turkey_caecum_content	EUCAMP2	0,5	>16	32	16	>64	>128	C. coli
2014	14-CA00815	Turkey_caecum_content	EUCAMP2	1	2	4	<=0,125	<=0,5	<=1	C. jejuni
2014	14-CA00816	Turkey_caecum_content	EUCAMP2	0,5	2	<=1	>16	64	<=1	C. jejuni
2014	14-CA00817	Turkey_caecum_content	EUCAMP2	1	4	>64	16	<=0,5	2	C. coli
2014	14-CA00825	Broiler_caecum_content	EUCAMP2	0,5	1	4	<=0,125	<=0,5	2	C. jejuni
2014	14-CA00826	Broiler_caecum_content	EUCAMP2	0,5	1	4	0,25	16	<=1	C. jejuni
2014	14-CA00845	Broiler_caecum_content	EUCAMP2	<=0,125	<=0,25	8	0,25	<=0,5	<=1	C. jejuni

Table S5: Minimum inhibitory concentrations of 6 antimicrobials for isolats of C. jejuni and C. coli from poultry

Year	Sample No.	Matrix_final	Antimicrobial panel	GEN	STR	NAL	CIP	TET	ERY	Species
2014	14-CA00846	Broiler_caecum_content	EUCAMP2	0,5	2	64	>16	>64	2	C. coli
2014	14-CA00882	Broiler_caecum_content	EUCAMP2	1	2	8	0,25	64	<=1	C. coli
2014	14-CA00882	Broiler_caecum_content	EUCAMP2	0,5	1	4	0,25	<=0,5	<=1	C. jejuni
2014	14-CA00883	Broiler_caecum_content	EUCAMP2	<=0,125	<=0,25	32	8	<=0,5	<=1	C. jejuni
2014	14-CA00884	Broiler_caecum_content	EUCAMP2	0,5	1	64	16	<=0,5	<=1	C. jejuni
2014	14-CA00885	Broiler_caecum_content	EUCAMP2	0,5	0,5	8	0,25	<=0,5	<=1	C. jejuni
2014	14-CA00934	Broiler_caecum_content	EUCAMP2	1	2	64	16	64	<=1	C. coli
2014	14-CA00935	Broiler_caecum_content	EUCAMP2	1	4	>64	16	64	<=1	C. coli
2014	14-CA00985	Turkey_caecum_content	EUCAMP2	0,5	1	>64	>16	<=0,5	<=1	C. jejuni
2014	14-CA00986	Turkey_caecum_content	EUCAMP2	0,5	2	64	16	64	<=1	C. jejuni
2014	14-CA00987	Turkey_caecum_content	EUCAMP2	0,5	1	64	>16	16	<=1	C. jejuni
2014	14-CA00988	Turkey_caecum_content	EUCAMP2	0,5	1	64	16	<=0,5	<=1	C. jejuni
2014	14-CA00997	Turkey_caecum_content	EUCAMP2	0,25	0,5	64	>16	>64	<=1	C. jejuni
2014	14-CA00998	Broiler_caecum_content	EUCAMP2	0,25	0,5	32	>16	>64	>128	C. jejuni
2014	14-CA00999	Broiler_caecum_content	EUCAMP2	0,25	0,5	64	>16	>64	>128	C. jejuni
2014	14-CA01000	Broiler_caecum_content	EUCAMP2	0,5	2	2	>16	>64	<=1	C. jejuni
2014	14-CA01001	Broiler_caecum_content	EUCAMP2	0,25	1	32	16	<=0,5	<=1	C. jejuni
2014	14-CA01006	Turkey_caecum_content	EUCAMP2	<=0,125	0,5	32	16	64	>128	C. coli
2014	14-CA01007	Turkey_caecum_content	EUCAMP2	<=0,125	0,5	32	16	64	128	C. coli
2014	14-CA01008	Turkey_caecum_content	EUCAMP2	0,5	2	4	<=0,125	<=0,5	<=1	C. jejuni
2014	14-CA01009	Turkey_caecum_content	EUCAMP2	0,5	2	4	0,25	<=0,5	<=1	C. jejuni
2014	14-CA01010	Turkey_caecum_content	EUCAMP2	1	4	64	16	64	<=1	C. coli
2014	14-CA01011	Turkey_caecum_content	EUCAMP2	1	4	>64	2	<=0,5	16	C. jejuni
2014	14-CA01012	Turkey_caecum_content	EUCAMP2	0,5	2	64	>16	64	>128	C. coli
2014	14-CA01013	Turkey_caecum_content	EUCAMP2	0,5	1	64	16	<=0,5	<=1	C. jejuni
2014	14-CA01014	Turkey_caecum_content	EUCAMP2	0,25	1	64	16	64	<=1	C. jejuni
2014	14-CA01015	Turkey_caecum_content	EUCAMP2	0,5	2	64	>16	>64	>128	C. coli
2014	14-CA01016	Turkey_caecum_content	EUCAMP2	0,5	>16	>64	>16	<=0,5	2	C. coli
2014	14-CA01017	Turkey_caecum_content	EUCAMP2	0,5	>16	64	16	64	<=1	C. coli
2014	14-CA01018	Broiler_caecum_content	EUCAMP2	0,5	1	8	0,5	32	<=1	C. coli
2014	14-CA01019	Broiler_caecum_content	EUCAMP2	0,25	1	16	8	32	128	C. coli
2014	14-CA01020	Broiler_caecum_content	EUCAMP2	<=0,125	<=0,25	2	<=0,125	32	<=1	C. coli

Table S5: Minimum inhibitory concentrations of 6 antimicrobials for isolats of C. jejuni and C. coli from poultry

Year	Sample No.	Matrix_final	Antimicrobial panel	GEN	STR	NAL	CIP	TET	ERY	Species
2014	14-CA01021	Broiler_caecum_content	EUCAMP2	0,5	1	64	16	64	<=1	C. jejuni
2014	14-CA01022	Broiler_caecum_content	EUCAMP2	1	2	64	>16	>64	<=1	C. coli
2014	14-CA01023	Broiler_caecum_content	EUCAMP2	0,25	2	4	0,25	<=0,5	<=1	C. jejuni
2014	14-CA01024	Broiler_caecum_content	EUCAMP2	0,25	1	64	16	>64	<=1	C. coli
2014	14-CA01025	Broiler_caecum_content	EUCAMP2	0,25	2	64	>16	>64	<=1	C. coli
2014	14-CA01026	Turkey_caecum_content	EUCAMP2	0,5	2	>64	>16	<=0,5	2	C. jejuni
2014	14-CA01027	Turkey_caecum_content	EUCAMP2	<=0,125	0,5	16	16	32	<=1	C. coli
2014	14-CA01028	Turkey_caecum_content	EUCAMP2	0,5	>16	4	<=0,125	64	<=1	C. jejuni
2014	14-CA01029	Turkey_caecum_content	EUCAMP2	0,5	2	64	>16	64	<=1	C. jejuni
2014	14-CA01030	Turkey_caecum_content	EUCAMP2	0,5	4	16	16	<=0,5	32	C. coli
2014	14-CA01031	Turkey_caecum_content	EUCAMP2	0,25	0,5	32	>16	>64	<=1	C. jejuni
2014	14-CA01032	Turkey_caecum_content	EUCAMP2	0,5	2	32	>16	64	>128	C. jejuni
2014	14-CA01033	Turkey_caecum_content	EUCAMP2	0,25	1	32	16	64	128	C. jejuni
2014	14-CA01034	Broiler_caecum_content	EUCAMP2	<=0,125	2	16	8	32	4	C. coli
2014	14-CA01035	Broiler_caecum_content	EUCAMP2	0,25	1	32	>16	>64	<=1	C. jejuni
2014	14-CA01036	Broiler_caecum_content	EUCAMP2	0,5	1	64	>16	>64	<=1	C. coli
2014	14-CA01037	Broiler_caecum_content	EUCAMP2	0,5	1	>64	>16	>64	<=1	C. jejuni
2014	14-CA01038	Broiler_caecum_content	EUCAMP2	0,5	1	64	16	>64	2	C. jejuni
2014	14-CA01039	Broiler_caecum_content	EUCAMP2	0,25	0,5	32	>16	>64	>128	C. jejuni
2014	14-CA01040	Broiler_caecum_content	EUCAMP2	0,5	1	>64	>16	>64	<=1	C. jejuni
2014	14-CA01041	Broiler_caecum_content	EUCAMP2	0,5	1	>64	>16	>64	4	C. coli
2014	14-CA01042	Broiler_caecum_content	EUCAMP2	<=0,125	1	64	>16	64	<=1	C. jejuni
2014	14-CA01043	Turkey_caecum_content	EUCAMP2	0,5	2	64	>16	>64	>128	C. coli
2014	14-CA01044	Turkey_caecum_content	EUCAMP2	0,25	1	64	>16	>64	<=1	C. jejuni
2014	14-CA01045	Turkey_caecum_content	EUCAMP2	0,25	1	64	16	<=0,5	<=1	C. jejuni
2014	14-CA01046	Turkey_caecum_content	EUCAMP2	<=0,125	0,5	64	16	64	<=1	C. jejuni
2014	14-CA01047	Turkey_caecum_content	EUCAMP2	2	>16	>64	>16	>64	4	C. coli
2014	14-CA01048	Turkey_caecum_content	EUCAMP2	0,5	2	4	0,5	<=0,5	2	C. jejuni
2014	14-CA01049	Turkey_caecum_content	EUCAMP2	0,25	1	32	16	32	<=1	C. jejuni
2014	14-CA01050	Turkey_caecum_content	EUCAMP2	0,5	1	16	4	32	32	C. coli
2014	14-CA01051	Turkey_caecum_content	EUCAMP2	0,5	1	64	16	64	<=1	C. coli
2014	14-CA01052	Turkey_caecum_content	EUCAMP2	0,5	2	4	0,25	<=0,5	<=1	C. jejuni

Table S5: Minimum inhibitory concentrations of 6 antimicrobials for isolats of C. jejuni and C. coli from poultry

Year	Sample No.	Matrix_final	Antimicrobial panel	GEN	STR	NAL	CIP	TET	ERY	Species
2014	14-CA01053	Broiler_caecum_content	EUCAMP2	0,25	0,5	>64	>16	64	<=1	C. jejuni
2014	14-CA01054	Broiler_caecum_content	EUCAMP2	0,25	2	64	16	64	<=1	C. coli
2014	14-CA01055	Broiler_caecum_content	EUCAMP2	0,5	2	4	0,25	<=0,5	<=1	C. jejuni
2014	14-CA01056	Broiler_caecum_content	EUCAMP2	0,5	2	64	>16	64	>128	C. coli
2014	14-CA01057	Broiler_caecum_content	EUCAMP2	1	2	>64	16	64	<=1	C. coli
2014	14-CA01058	Broiler_caecum_content	EUCAMP2	0,5	2	64	>16	>64	>128	C. coli
2014	14-CA01059	Broiler_caecum_content	EUCAMP2	1	4	64	>16	>64	<=1	C. coli
2014	14-CA01060	Broiler_caecum_content	EUCAMP2	0,5	>16	64	>16	>64	>128	C. coli
2014	14-CA01061	Broiler_caecum_content	EUCAMP2	0,5	>16	32	16	>64	>128	C. coli
2014	14-CA01062	Broiler_caecum_content	EUCAMP2	0,25	0,5	<=1	16	64	<=1	C. jejuni
2014	14-CA01063	Broiler_caecum_content	EUCAMP2	1	>16	4	0,25	64	2	C. coli
2014	14-CA01064	Broiler_caecum_content	EUCAMP2	0,5	2	>64	8	<=0,5	2	C. jejuni
2014	14-CA01065	Turkey_caecum_content	EUCAMP2	0,25	1	4	<=0,125	<=0,5	<=1	C. jejuni
2014	14-CA01066	Turkey_caecum_content	EUCAMP2	0,5	>16	64	>16	>64	>128	C. coli
2014	14-CA01067	Turkey_caecum_content	EUCAMP2	0,5	>16	64	>16	>64	>128	C. coli
2014	14-CA01068	Turkey_caecum_content	EUCAMP2	0,5	2	4	<=0,125	64	<=1	C. jejuni
2014	14-CA01069	Turkey_caecum_content	EUCAMP2	0,5	2	64	16	64	<=1	C. coli
2014	14-CA01070	Turkey_caecum_content	EUCAMP2	0,5	2	2	<=0,125	64	<=1	C. jejuni
2014	14-CA01071	Broiler_caecum_content	EUCAMP2	0,5	1	4	<=0,125	<=0,5	<=1	C. jejuni
2014	14-CA01072	Broiler_caecum_content	EUCAMP2	0,5	1	4	<=0,125	>64	<=1	C. jejuni
2014	14-CA01073	Broiler_caecum_content	EUCAMP2	0,5	1	2	<=0,125	>64	<=1	C. jejuni
2014	14-CA01074	Broiler_caecum_content	EUCAMP2	0,5	2	64	16	>64	<=1	C. jejuni
2014	14-CA01075	Turkey_caecum_content	EUCAMP2	1	4	>64	>16	>64	<=1	C. jejuni
2014	14-CA01076	Turkey_caecum_content	EUCAMP2	1	4	>64	8	<=0,5	<=1	C. coli
2014	14-CA01077	Turkey_caecum_content	EUCAMP2	0,25	0,5	4	0,25	>64	<=1	C. jejuni
2014	14-CA01078	Turkey_caecum_content	EUCAMP2	0,5	2	64	16	>64	<=1	C. jejuni
2014	14-CA01079	Broiler_caecum_content	EUCAMP2	0,5	1	64	>16	>64	<=1	C. jejuni
2014	14-CA01080	Broiler_caecum_content	EUCAMP2	1	2	4	0,25	<=0,5	<=1	C. jejuni
2014	14-CA01081	Broiler_caecum_content	EUCAMP2	0,5	4	4	<=0,125	16	<=1	C. jejuni
2014	14-CA01082	Broiler_caecum_content	EUCAMP2	0,25	1	64	>16	>64	<=1	C. jejuni
2014	14-CA01083	Turkey_caecum_content	EUCAMP2	0,25	1	64	>16	>64	<=1	C. jejuni
2014	14-CA01084	Turkey_caecum_content	EUCAMP2	0,5	2	4	<=0,125	<=0,5	<=1	C. jejuni

Table S5: Minimum inhibitory concentrations of 6 antimicrobials for isolats of C. jejuni and C. coli from poultry

Year	Sample No.	Matrix_final	Antimicrobial panel	GEN	STR	NAL	CIP	TET	ERY	Species
2014	14-CA01085	Turkey_caecum_content	EUCAMP2	0,5	2	4	<=0,125	<=0,5	<=1	C. jejuni
2014	14-CA01086	Turkey_caecum_content	EUCAMP2	0,5	1	64	16	>64	<=1	C. jejuni
2014	14-CA01087	Turkey_caecum_content	EUCAMP2	0,5	2	>64	16	>64	2	C. jejuni
2014	14-CA01088	Turkey_caecum_content	EUCAMP2	0,5	1	4	<=0,125	<=0,5	<=1	C. jejuni
2014	14-CA01089	Turkey_caecum_content	EUCAMP2	0,5	1	64	16	<=0,5	<=1	C. jejuni
2014	14-CA01090	Turkey_caecum_content	EUCAMP2	0,5	1	64	16	<=0,5	<=1	C. jejuni
2014	14-CA01091	Broiler_caecum_content	EUCAMP2	0,5	1	64	16	<=0,5	<=1	C. jejuni
2014	14-CA01092	Broiler_caecum_content	EUCAMP2	0,5	1	>64	16	<=0,5	<=1	C. jejuni
2014	14-CA01093	Broiler_caecum_content	EUCAMP2	0,5	1	16	0,5	>64	<=1	C. jejuni
2014	14-CA01094	Broiler_caecum_content	EUCAMP2	0,5	2	64	16	<=0,5	<=1	C. jejuni
2014	14-CA01095	Broiler_caecum_content	EUCAMP2	0,5	1	16	0,5	>64	<=1	C. jejuni
2014	14-CA01096	Broiler_caecum_content	EUCAMP2	0,25	1	64	8	64	<=1	C. jejuni
2014	14-CA01097	Broiler_caecum_content	EUCAMP2	0,5	>16	64	>16	>64	>128	C. coli
2014	14-CA01098	Broiler_caecum_content	EUCAMP2	0,5	>16	64	>16	>64	>128	C. coli
2014	14-CA01099	Broiler_caecum_content	EUCAMP2	0,5	>16	64	>16	64	>128	C. coli
2014	14-CA01100	Broiler_caecum_content	EUCAMP2	0,5	>16	64	>16	>64	>128	C. coli
2014	14-CA01101	Broiler_caecum_content	EUCAMP2	0,5	>16	64	>16	>64	>128	C. coli
2014	14-CA01108	Broiler_caecum_content	EUCAMP2	0,5	1	8	0,25	<=0,5	<=1	C. jejuni
2014	14-CA01109	Broiler_caecum_content	EUCAMP2	0,5	2	4	<=0,125	<=0,5	<=1	C. coli
2014	14-CA01119	Broiler_caecum_content	EUCAMP2	0,5	1	64	16	>64	<=1	C. jejuni
2014	14-CA01120	Broiler_caecum_content	EUCAMP2	0,5	2	4	0,25	<=0,5	<=1	C. jejuni
2014	14-CA01121	Broiler_caecum_content	EUCAMP2	<=0,125	<=0,25	32	16	<=0,5	<=1	C. jejuni
2014	14-CA01122	Broiler_caecum_content	EUCAMP2	1	2	4	<=0,125	>64	<=1	C. coli
2014	14-CA01123	Broiler_caecum_content	EUCAMP2	0,5	2	8	0,25	<=0,5	<=1	C. jejuni
2014	14-CA01124	Broiler_caecum_content	EUCAMP2	0,25	2	64	>16	>64	<=1	C. coli
2014	14-CA01159	Broiler_caecum_content	EUCAMP2	0,5	1	4	16	<=0,5	<=1	C. jejuni
2014	14-CA01160	Broiler_caecum_content	EUCAMP2	0,5	1	64	16	<=0,5	<=1	C. jejuni
2014	14-CA01161	Broiler_caecum_content	EUCAMP2	1	2	4	<=0,125	>64	<=1	C. coli
2014	14-CA01162	Broiler_caecum_content	EUCAMP2	0,5	1	32	16	64	<=1	C. jejuni
2014	14-CA01163	Broiler_caecum_content	EUCAMP2	0,5	2	64	16	64	<=1	C. jejuni
2014	14-CA01164	Broiler_caecum_content	EUCAMP2	0,5	2	64	16	64	2	C. coli
2014	14-CA01165	Turkey_caecum_content	EUCAMP2	0,5	2	>64	>16	<=0,5	2	C. jejuni

Table S5: Minimum inhibitory concentrations of 6 antimicrobials for isolats of C. jejuni and C. coli from poultry

Year	Sample No.	Matrix_final	Antimicrobial panel	GEN	STR	NAL	CIP	TET	ERY	Species
2014	14-CA01166	Turkey_caecum_content	EUCAMP2	0,5	2	>64	>16	>64	2	C. coli
2014	14-CA01167	Turkey_caecum_content	EUCAMP2	0,5	1	64	16	<=0,5	<=1	C. jejuni
2014	14-CA01207	Broiler_caecum_content	EUCAMP2	0,5	1	>64	>16	>64	<=1	C. coli
2014	14-CA01209	Turkey_caecum_content	EUCAMP2	0,25	2	4	<=0,125	<=0,5	<=1	C. jejuni
2014	14-CA01212	Turkey_caecum_content	EUCAMP2	1	4	>64	>16	>64	2	C. coli
2014	14-CA01213	Broiler_caecum_content	EUCAMP2	0,25	1	32	16	>64	<=1	C. coli
2014	14-CA01214	Broiler_caecum_content	EUCAMP2	0,5	2	64	16	64	<=1	C. coli
2014	14-CA01216	Turkey_caecum_content	EUCAMP2	0,5	>16	>64	>16	64	>128	C. coli
2014	14-CA01220	Turkey_caecum_content	EUCAMP2	0,25	0,5	32	16	32	<=1	C. jejuni
2014	14-CA01221	Turkey_caecum_content	EUCAMP2	0,5	2	4	0,25	<=0,5	<=1	C. jejuni
2014	14-CA01227	Turkey_caecum_content	EUCAMP2	0,5	2	64	16	<=0,5	<=1	C. jejuni
2014	14-CA01232	Broiler_caecum_content	EUCAMP2	0,5	2	4	<=0,125	<=0,5	<=1	C. coli
2014	14-CA01236	Turkey_caecum_content	EUCAMP2	0,5	>16	64	16	64	<=1	C. coli
2014	14-CA01246	Turkey_caecum_content	EUCAMP2	0,5	>16	64	>16	64	<=1	C. coli
2014	14-CA01248	Broiler_caecum_content	EUCAMP2	0,5	1	64	16	<=0,5	<=1	C. coli
2014	14-CA01249	Broiler_caecum_content	EUCAMP2	0,5	2	>64	16	64	<=1	C. coli
2014	14-CA01250	Turkey_caecum_content	EUCAMP2	0,5	2	64	8	<=0,5	<=1	C. coli
2014	14-CA01251	Turkey_caecum_content	EUCAMP2	0,25	0,5	32	16	<=0,5	<=1	C. coli
2014	14-CA01252	Turkey_caecum_content	EUCAMP2	0,5	2	4	<=0,125	<=0,5	<=1	C. jejuni
2014	14-CA01259	Turkey_caecum_content	EUCAMP2	0,25	1	32	8	32	<=1	C. coli
2014	14-CA01278	Turkey_caecum_content	EUCAMP2	0,5	1	>64	16	<=0,5	<=1	C. jejuni
2014	14-CA01279	Broiler_caecum_content	EUCAMP2	0,5	2	64	>16	<=0,5	<=1	C. coli
2014	14-CA01280	Turkey_caecum_content	EUCAMP2	0,25	1	4	<=0,125	<=0,5	<=1	C. jejuni
2014	14-CA01281	Broiler_caecum_content	EUCAMP2	0,25	1	64	16	<=0,5	<=1	C. coli
2014	14-CA01310	Broiler_caecum_content	EUCAMP2	0,5	4	64	16	64	<=1	C. coli
2014	14-CA01311	Turkey_caecum_content	EUCAMP2	0,25	0,5	64	>16	8	<=1	C. jejuni
2014	14-CA01312	Turkey_caecum_content	EUCAMP2	0,5	1	8	0,25	<=0,5	<=1	C. jejuni
2014	14-CA01319	Turkey_caecum_content	EUCAMP2	0,5	2	64	>16	<=0,5	<=1	C. coli
2014	14-CA01320	Turkey_caecum_content	EUCAMP2	<=0,125	<=0,25	4	<=0,125	<=0,5	<=1	C. jejuni
2014	14-CA01321	Turkey_caecum_content	EUCAMP2	0,5	2	8	0,5	64	2	C. coli
2014	14-CA01323	Turkey_caecum_content	EUCAMP2	<=0,125	1	4	<=0,125	<=0,5	<=1	C. jejuni
2014	14-CA01324	Turkey_caecum_content	EUCAMP2	0,5	1	64	>16	64	>128	C. coli

Table S5: Minimum inhibitory concentrations of 6 antimicrobials for isolats of C. jejuni and C. coli from poultry

Year	Sample No.	Matrix_final	Antimicrobial panel	GEN	STR	NAL	CIP	TET	ERY	Species
2014	14-CA01325	Turkey_caecum_content	EUCAMP2	0,5	>16	64	>16	64	4	C. coli
2014	14-CA01326	Turkey_caecum_content	EUCAMP2	0,5	>16	64	>16	64	4	C. coli
2014	14-CA01327	Turkey_caecum_content	EUCAMP2	0,25	2	64	>16	64	<=1	C. coli
2014	14-CA01328	Turkey_caecum_content	EUCAMP2	0,25	0,5	32	16	32	<=1	C. jejuni
2014	14-CA01329	Turkey_caecum_content	EUCAMP2	0,5	1	>64	>16	>64	<=1	C. jejuni
2014	14-CA01330	Broiler_caecum_content	EUCAMP2	0,25	1	64	16	<=0,5	<=1	C. jejuni
2014	14-CA01331	Turkey_caecum_content	EUCAMP2	0,5	1	>64	>16	<=0,5	2	C. coli
2014	14-CA01332	Turkey_caecum_content	EUCAMP2	0,5	1	64	16	64	<=1	C. coli
2014	14-CA01333	Turkey_caecum_content	EUCAMP2	<=0,125	0,5	8	8	32	>128	C. coli
2014	14-CA01334	Turkey_caecum_content	EUCAMP2	0,25	>16	>64	>16	>64	>128	C. coli
2014	14-CA01335	Turkey_caecum_content	EUCAMP2	0,25	1	64	>16	64	2	C. coli
2014	14-CA01336	Turkey_caecum_content	EUCAMP2	0,25	1	2	<=0,125	<=0,5	<=1	C. jejuni
2014	14-CA01337	Turkey_caecum_content	EUCAMP2	0,25	<=0,25	32	16	32	<=1	C. jejuni
2014	14-CA01338	Turkey_caecum_content	EUCAMP2	0,5	2	64	16	64	<=1	C. coli
2014	14-CA01339	Turkey_caecum_content	EUCAMP2	0,5	2	>64	>16	64	<=1	C. coli
2014	14-CA01340	Turkey_caecum_content	EUCAMP2	0,5	2	64	16	64	<=1	C. coli
2014	14-CA01341	Turkey_caecum_content	EUCAMP2	0,5	1	4	<=0,125	<=0,5	<=1	C. jejuni
2014	14-CA01342	Turkey_caecum_content	EUCAMP2	0,25	1	64	>16	64	>128	C. coli
2014	14-CA01343	Broiler_caecum_content	EUCAMP2	0,5	2	64	>16	>64	<=1	C. coli
2014	14-CA01344	Turkey_caecum_content	EUCAMP2	0,25	1	64	>16	>64	<=1	C. coli
2014	14-CA01345	Turkey_caecum_content	EUCAMP2	0,5	2	32	8	32	<=1	C. coli
2014	14-CA01346	Turkey_caecum_content	EUCAMP2	0,5	2	64	>16	>64	<=1	C. coli
2014	14-CA01347	Turkey_caecum_content	EUCAMP2	0,25	1	64	>16	64	>128	C. coli
2014	14-CA01348	Turkey_caecum_content	EUCAMP2	0,5	2	64	16	32	<=1	C. coli
2014	14-CA01349	Turkey_caecum_content	EUCAMP2	0,5	1	64	16	>64	>128	C. coli
2014	14-CA01350	Broiler_caecum_content	EUCAMP2	0,5	1	64	16	>64	<=1	C. jejuni
2014	14-CA01351	Broiler_caecum_content	EUCAMP2	0,5	1	8	0,25	<=0,5	<=1	C. jejuni
2014	14-CA01352	Broiler_caecum_content	EUCAMP2	0,25	1	64	16	<=0,5	<=1	C. jejuni
2014	14-CA01353	Broiler_caecum_content	EUCAMP2	0,5	2	64	16	<=0,5	<=1	C. jejuni
2014	14-CA01354	Broiler_caecum_content	EUCAMP2	<=0,125	<=0,25	64	16	>64	<=1	C. jejuni
2014	14-CA01355	Turkey_caecum_content	EUCAMP2	1	2	64	16	64	<=1	C. coli
2014	14-CA01375	Turkey_caecum_content	EUCAMP2	0,5	1	4	<=0,125	<=0,5	<=1	C. jejuni

Table S5: Minimum inhibitory concentrations of 6 antimicrobials for isolats of C. jejuni and C. coli from poultry

Year	Sample No.	Matrix_final	Antimicrobial panel	GEN	STR	NAL	CIP	TET	ERY	Species
2014	14-CA01383	Broiler_caecum_content	EUCAMP2	0,5	1	64	16	>64	<=1	C. jejuni
2014	14-CA01394	Broiler_caecum_content	EUCAMP2	0,25	<=0,25	4	<=0,125	<=0,5	<=1	C. jejuni
2014	14-CA01395	Broiler_caecum_content	EUCAMP2	<=0,125	<=0,25	4	<=0,125	<=0,5	<=1	C. jejuni
2014	14-CA01411	Broiler_caecum_content	EUCAMP2	0,5	2	2	0,25	>64	<=1	C. jejuni
2014	14-CA01423	Turkey_caecum_content	EUCAMP2	0,5	2	>64	16	4	<=1	C. jejuni
2014	14-CA01424	Turkey_caecum_content	EUCAMP2	0,25	>16	>64	>16	<=0,5	<=1	C. jejuni
2014	14-CA01425	Turkey_caecum_content	EUCAMP2	0,5	2	64	>16	64	>128	C. coli
2014	14-CA01426	Turkey_caecum_content	EUCAMP2	1	2	>64	>16	>64	<=1	C. coli
2014	14-CA01427	Broiler_caecum_content	EUCAMP2	0,25	2	2	<=0,125	<=0,5	<=1	C. jejuni
2014	14-CA01428	Broiler_caecum_content	EUCAMP2	<=0,125	<=0,25	32	8	32	<=1	C. jejuni
2014	14-CA01429	Broiler_caecum_content	EUCAMP2	0,5	2	32	>16	>64	<=1	C. jejuni
2014	14-CA01430	Turkey_caecum_content	EUCAMP2	1	2	4	0,25	<=0,5	<=1	C. jejuni
2014	14-CA01431	Turkey_caecum_content	EUCAMP2	0,5	2	8	0,5	<=0,5	<=1	C. coli
2014	14-CA01432	Broiler_caecum_content	EUCAMP2	0,5	2	>64	>16	>64	<=1	C. coli
2014	14-CA01433	Broiler_caecum_content	EUCAMP2	0,5	1	64	16	32	<=1	C. jejuni
2014	14-CA01434	Broiler_caecum_content	EUCAMP2	0,25	1	2	<=0,125	<=0,5	<=1	C. jejuni
2014	14-CA01435	Broiler_caecum_content	EUCAMP2	0,25	1	32	16	32	<=1	C. jejuni
2014	14-CA01436	Turkey_caecum_content	EUCAMP2	1	4	>64	>16	<=0,5	<=1	C. coli
2014	14-CA01437	Turkey_caecum_content	EUCAMP2	0,5	2	>64	>16	>64	<=1	C. coli
2014	14-CA01438	Turkey_caecum_content	EUCAMP2	0,5	2	>64	>16	>64	<=1	C. coli
2014	14-CA01439	Turkey_caecum_content	EUCAMP2	0,5	2	64	>16	64	>128	C. coli
2014	14-CA01440	Turkey_caecum_content	EUCAMP2	1	>16	4	0,25	16	>128	C. coli
2014	14-CA01441	Turkey_caecum_content	EUCAMP2	0,5	1	4	<=0,125	<=0,5	<=1	C. jejuni
2014	14-CA01442	Turkey_caecum_content	EUCAMP2	1	1	4	<=0,125	<=0,5	<=1	C. jejuni
2014	14-CA01443	Turkey_caecum_content	EUCAMP2	0,5	2	64	>16	>64	<=1	C. jejuni
2014	14-CA01444	Turkey_caecum_content	EUCAMP2	0,5	1	>64	>16	<=0,5	<=1	C. jejuni
2014	14-CA01445	Broiler_caecum_content	EUCAMP2	1	2	64	>16	>64	<=1	C. coli
2014	14-CA01446	Broiler_caecum_content	EUCAMP2	<=0,125	0,5	32	>16	>64	<=1	C. jejuni
2014	14-CA01447	Broiler_caecum_content	EUCAMP2	0,25	1	2	16	<=0,5	<=1	C. jejuni
2014	14-CA01448	Broiler_caecum_content	EUCAMP2	0,5	1	4	<=0,125	<=0,5	<=1	C. jejuni
2014	14-CA01449	Broiler_caecum_content	EUCAMP2	0,25	1	32	16	32	<=1	C. jejuni
2014	14-CA01450	Broiler_caecum_content	EUCAMP2	0,25	1	2	<=0,125	<=0,5	<=1	C. jejuni

Table S5: Minimum inhibitory concentrations of 6 antimicrobials for isolats of C. jejuni and C. coli from poultry

Year	Sample No.	Matrix_final	Antimicrobial panel	GEN	STR	NAL	CIP	TET	ERY	Species
2014	14-CA01451	Broiler_caecum_content	EUCAMP2	0,5	0,5	64	>16	>64	>128	C. coli
2014	14-CA01452	Broiler_caecum_content	EUCAMP2	0,5	1	4	<=0,125	64	<=1	C. jejuni
2014	14-CA01453	Broiler_caecum_content	EUCAMP2	0,25	0,5	64	>16	>64	<=1	C. jejuni
2014	14-CA01454	Turkey_caecum_content	EUCAMP2	0,5	2	>64	>16	64	>128	C. coli
2014	14-CA01455	Turkey_caecum_content	EUCAMP2	<=0,125	<=0,25	32	16	64	<=1	C. jejuni
2014	14-CA01456	Turkey_caecum_content	EUCAMP2	0,25	>16	32	16	32	2	C. coli
2014	14-CA01457	Turkey_caecum_content	EUCAMP2	0,5	2	64	>16	>64	2	C. coli
2014	14-CA01458	Broiler_caecum_content	EUCAMP2	0,25	1	64	>16	>64	<=1	C. jejuni
2014	14-CA01459	Broiler_caecum_content	EUCAMP2	0,5	0,5	64	16	<=0,5	<=1	C. jejuni
2014	14-CA01460	Broiler_caecum_content	EUCAMP2	0,5	2	>64	>16	>64	2	C. coli
2014	14-CA01461	Broiler_caecum_content	EUCAMP2	0,5	2	64	8	<=0,5	<=1	C. jejuni
2014	14-CA01462	Broiler_caecum_content	EUCAMP2	1	2	>64	16	<=0,5	<=1	C. jejuni
2014	14-CA01463	Broiler_caecum_content	EUCAMP2	0,5	2	2	>16	8	<=1	C. jejuni
2014	14-CA01464	Broiler_caecum_content	EUCAMP2	0,25	1	4	<=0,125	<=0,5	<=1	C. jejuni
2014	14-CA01465	Turkey_caecum_content	EUCAMP2	0,5	1	64	16	64	<=1	C. coli
2014	14-CA01466	Turkey_caecum_content	EUCAMP2	0,25	1	64	>16	>64	<=1	C. coli
2014	14-CA01467	Turkey_caecum_content	EUCAMP2	0,5	2	64	16	64	<=1	C. coli
2014	14-CA01468	Turkey_caecum_content	EUCAMP2	0,5	1	32	>16	8	<=1	C. jejuni
2014	14-CA01469	Turkey_caecum_content	EUCAMP2	0,5	2	64	>16	64	>128	C. coli
2014	14-CA01470	Turkey_caecum_content	EUCAMP2	0,5	1	32	16	64	<=1	C. jejuni
2014	14-CA01471	Turkey_caecum_content	EUCAMP2	0,5	1	>64	>16	64	<=1	C. coli
2014	14-CA01472	Turkey_caecum_content	EUCAMP2	0,5	2	64	>16	64	>128	C. coli
2014	14-CA01473	Broiler_caecum_content	EUCAMP2	0,25	1	4	<=0,125	<=0,5	<=1	C. jejuni
2014	14-CA01474	Broiler_caecum_content	EUCAMP2	0,25	1	4	<=0,125	<=0,5	<=1	C. jejuni
2014	14-CA01475	Broiler_caecum_content	EUCAMP2	0,25	1	2	16	4	<=1	C. jejuni
2014	14-CA01476	Broiler_caecum_content	EUCAMP2	<=0,125	<=0,25	32	16	32	<=1	C. jejuni
2014	14-CA01477	Broiler_caecum_content	EUCAMP2	0,5	2	4	<=0,125	<=0,5	<=1	C. coli
2014	14-CA01478	Broiler_caecum_content	EUCAMP2	<=0,125	<=0,25	32	>16	>64	>128	C. jejuni
2014	14-CA01479	Turkey_caecum_content	EUCAMP2	0,25	0,5	2	<=0,125	64	<=1	C. jejuni
2014	14-CA01480	Turkey_caecum_content	EUCAMP2	0,25	1	32	16	64	>128	C. coli
2014	14-CA01481	Turkey_caecum_content	EUCAMP2	1	4	64	16	64	<=1	C. coli
2014	14-CA01482	Turkey_caecum_content	EUCAMP2	0,5	2	>64	>16	>64	<=1	C. coli

Table S5: Minimum inhibitory concentrations of 6 antimicrobials for isolats of C. jejuni and C. coli from poultry

Year	Sample No.	Matrix_final	Antimicrobial panel	GEN	STR	NAL	CIP	TET	ERY	Species
2014	14-CA01483	Turkey_caecum_content	EUCAMP2	0,5	1	64	16	64	<=1	C. coli
2014	14-CA01484	Turkey_caecum_content	EUCAMP2	0,5	2	>64	>16	64	<=1	C. coli
2014	14-CA01485	Turkey_caecum_content	EUCAMP2	0,5	2	4	<=0,125	<=0,5	<=1	C. jejuni
2014	14-CA01486	Turkey_caecum_content	EUCAMP2	0,5	>16	64	>16	64	>128	C. coli
2014	14-CA01487	Turkey_caecum_content	EUCAMP2	<=0,125	1	16	8	32	>128	C. coli
2014	14-CA01488	Turkey_caecum_content	EUCAMP2	0,5	1	64	>16	>64	>128	C. coli
2014	14-CA01489	Turkey_caecum_content	EUCAMP2	0,25	0,5	4	<=0,125	<=0,5	<=1	C. jejuni
2014	14-CA01490	Turkey_caecum_content	EUCAMP2	0,5	1	64	>16	64	>128	C. coli
2014	14-CA01491	Turkey_caecum_content	EUCAMP2	0,25	2	64	>16	64	>128	C. coli
2014	14-CA01492	Broiler_caecum_content	EUCAMP2	0,5	1	4	<=0,125	<=0,5	<=1	C. jejuni
2014	14-CA01493	Broiler_caecum_content	EUCAMP2	0,25	0,5	64	16	64	<=1	C. jejuni
2014	14-CA01494	Broiler_caecum_content	EUCAMP2	0,5	>16	>64	>16	>64	>128	C. coli
2014	14-CA01495	Broiler_caecum_content	EUCAMP2	1	2	64	>16	>64	<=1	C. coli
2014	14-CA01496	Broiler_caecum_content	EUCAMP2	1	2	64	16	>64	<=1	C. jejuni
2014	14-CA01497	Broiler_caecum_content	EUCAMP2	0,5	2	8	0,25	<=0,5	<=1	C. jejuni
2014	14-CA01498	Broiler_caecum_content	EUCAMP2	0,5	0,5	>64	>16	<=0,5	<=1	C. jejuni
2014	14-CA01499	Broiler_caecum_content	EUCAMP2	0,5	0,5	64	16	>64	>128	C. coli
2014	14-CA01500	Broiler_caecum_content	EUCAMP2	0,25	0,5	64	>16	>64	4	C. jejuni
2014	14-CA01501	Turkey_caecum_content	EUCAMP2	0,5	>16	64	>16	64	>128	C. coli
2014	14-CA01502	Turkey_caecum_content	EUCAMP2	0,25	0,5	64	16	32	<=1	C. jejuni
2014	14-CA01503	Turkey_caecum_content	EUCAMP2	0,5	1	>64	>16	<=0,5	2	C. jejuni
2014	14-CA01504	Turkey_caecum_content	EUCAMP2	1	2	32	8	>64	<=1	C. coli
2014	14-CA01505	Turkey_caecum_content	EUCAMP2	0,5	2	64	>16	>64	2	C. coli
2014	14-CA01506	Turkey_caecum_content	EUCAMP2	1	4	>64	>16	>64	4	C. coli
2014	14-CA01507	Broiler_caecum_content	EUCAMP2	0,5	>16	64	>16	64	>128	C. coli
2014	14-CA01508	Turkey_caecum_content	EUCAMP2	0,25	>16	16	16	32	<=1	C. coli
2014	14-CA01509	Turkey_caecum_content	EUCAMP2	0,5	2	8	0,25	<=0,5	<=1	C. jejuni
2014	14-CA01510	Turkey_caecum_content	EUCAMP2	0,5	>16	64	>16	64	>128	C. coli
2014	14-CA01511	Turkey_caecum_content	EUCAMP2	0,25	0,5	16	8	32	<=1	C. coli
2014	14-CA01512	Turkey_caecum_content	EUCAMP2	0,5	2	64	>16	64	>128	C. coli
2014	14-CA01513	Broiler_caecum_content	EUCAMP2	0,5	1	<=1	16	8	<=1	C. jejuni
2014	14-CA01514	Broiler_caecum_content	EUCAMP2	0,5	1	64	>16	32	>128	C. coli

Table S5: Minimum inhibitory concentrations of 6 antimicrobials for isolats of C. jejuni and C. coli from poultry

Year	Sample No.	Matrix_final	Antimicrobial panel	GEN	STR	NAL	CIP	TET	ERY	Species
2014	14-CA01515	Broiler_caecum_content	EUCAMP2	0,5	2	4	0,25	<=0,5	<=1	C. jejuni
2014	14-CA01516	Broiler_caecum_content	EUCAMP2	1	2	>64	>16	>64	2	C. coli
2014	14-CA01517	Broiler_caecum_content	EUCAMP2	0,5	2	>64	>16	>64	2	C. coli
2014	14-CA01518	Broiler_caecum_content	EUCAMP2	0,5	2	64	16	64	<=1	C. coli
2014	14-CA01519	Turkey_caecum_content	EUCAMP2	0,25	1	8	0,5	32	<=1	C. jejuni
2014	14-CA01520	Turkey_caecum_content	EUCAMP2	0,5	1	64	>16	64	<=1	C. coli
2014	14-CA01521	Turkey_caecum_content	EUCAMP2	1	4	>64	16	>64	<=1	C. coli
2014	14-CA01522	Turkey_caecum_content	EUCAMP2	0,5	2	8	0,25	64	<=1	C. coli
2014	14-CA01523	Turkey_caecum_content	EUCAMP2	0,5	2	64	>16	64	>128	C. coli
2014	14-CA01524	Turkey_caecum_content	EUCAMP2	0,25	>16	64	16	64	>128	C. coli
2014	14-CA01525	Turkey_caecum_content	EUCAMP2	0,5	1	>64	16	>64	<=1	C. jejuni
2014	14-CA01526	Turkey_caecum_content	EUCAMP2	0,5	1	64	16	64	<=1	C. jejuni
2014	14-CA01527	Turkey_caecum_content	EUCAMP2	0,5	>16	64	16	64	>128	C. coli
2014	14-CA01528	Turkey_caecum_content	EUCAMP2	0,5	2	64	16	64	<=1	C. coli
2014	14-CA01529	Turkey_caecum_content	EUCAMP2	0,5	2	32	8	64	128	C. coli
2014	14-CA01530	Turkey_caecum_content	EUCAMP2	0,5	1	64	8	<=0,5	<=1	C. coli
2014	14-CA01531	Turkey_caecum_content	EUCAMP2	0,5	1	64	>16	64	>128	C. coli
2014	14-CA01532	Broiler_caecum_content	EUCAMP2	0,5	1	4	<=0,125	<=0,5	<=1	C. jejuni
2014	14-CA01533	Broiler_caecum_content	EUCAMP2	0,5	2	64	8	<=0,5	<=1	C. jejuni
2014	14-CA01534	Turkey_caecum_content	EUCAMP2	0,5	1	64	>16	64	<=1	C. coli
2014	14-CA01535	Broiler_caecum_content	EUCAMP2	1	4	64	16	64	<=1	C. coli
2014	14-CA01536	Broiler_caecum_content	EUCAMP2	0,5	2	64	>16	64	>128	C. coli
2014	14-CA01541	Turkey_caecum_content	EUCAMP2	0,5	2	64	16	<=0,5	<=1	C. jejuni
2014	14-CA01542	Broiler_caecum_content	EUCAMP2	0,25	0,5	64	>16	>64	<=1	C. coli
2014	14-CA01555	Turkey_caecum_content	EUCAMP2	0,5	2	>64	>16	64	<=1	C. coli
2014	14-CA01556	Turkey_caecum_content	EUCAMP2	<=0,125	0,5	64	>16	>64	<=1	C. jejuni
2014	14-CA01557	Turkey_caecum_content	EUCAMP2	0,5	1	16	8	16	<=1	C. coli
2014	14-CA01559	Broiler_caecum_content	EUCAMP2	0,5	1	32	1	<=0,5	<=1	C. jejuni
2014	14-CA01560	Broiler_caecum_content	EUCAMP2	0,5	1	64	16	<=0,5	<=1	C. jejuni
2014	14-CA01561	Broiler_caecum_content	EUCAMP2	0,25	0,5	64	16	64	<=1	C. jejuni
2014	14-CA01562	Broiler_caecum_content	EUCAMP2	0,5	2	64	16	<=0,5	<=1	C. coli
2014	14-CA01563	Broiler_caecum_content	EUCAMP2	0,25	0,5	>64	>16	>64	<=1	C. jejuni

Table S5: Minimum inhibitory concentrations of 6 antimicrobials for isolats of C. jejuni and C. coli from poultry

Year	Sample No.	Matrix_final	Antimicrobial panel	GEN	STR	NAL	CIP	TET	ERY	Species
2014	14-CA01564	Broiler_caecum_content	EUCAMP2	0,5	1	64	16	32	<=1	C. jejuni
2014	14-CA01565	Broiler_caecum_content	EUCAMP2	0,25	1	32	>16	64	<=1	C. jejuni
2014	14-CA01566	Broiler_caecum_content	EUCAMP2	0,25	0,5	64	>16	>64	<=1	C. jejuni
2014	14-CA01567	Broiler_caecum_content	EUCAMP2	0,5	1	64	16	<=0,5	<=1	C. coli
2014	14-CA01594	Broiler_caecum_content	EUCAMP2	1	2	4	<=0,125	<=0,5	<=1	C. coli
2014	14-CA01610	Broiler_caecum_content	EUCAMP2	0,25	1	32	16	32	<=1	C. jejuni
2014	14-CA01622	Broiler_caecum_content	EUCAMP2	1	2	64	16	<=0,5	<=1	C. jejuni
2014	14-CA01623	Turkey_caecum_content	EUCAMP2	0,5	1	64	16	<=0,5	<=1	C. jejuni
2014	14-CA01624	Turkey_caecum_content	EUCAMP2	0,5	2	64	>16	64	>128	C. coli
2014	14-CA01625	Turkey_caecum_content	EUCAMP2	1	2	64	16	>64	<=1	C. coli
2014	14-CA01626	Turkey_caecum_content	EUCAMP2	0,5	1	>64	>16	>64	<=1	C. coli
2014	14-CA01628	Turkey_caecum_content	EUCAMP2	1	2	>64	>16	<=0,5	4	C. coli
2014	14-CA01629	Turkey_caecum_content	EUCAMP2	0,5	1	64	8	<=0,5	<=1	C. jejuni
2014	14-CA01630	Turkey_caecum_content	EUCAMP2	1	2	64	16	64	<=1	C. coli
2014	14-CA01631	Broiler_caecum_content	EUCAMP2	0,5	1	4	<=0,125	64	<=1	C. jejuni
2014	14-CA01632	Broiler_caecum_content	EUCAMP2	1	>16	4	0,25	<=0,5	<=1	C. coli
2014	14-CA01633	Broiler_caecum_content	EUCAMP2	0,25	1	64	>16	>64	<=1	C. jejuni
2014	14-CA01634	Broiler_caecum_content	EUCAMP2	0,5	2	>64	>16	>64	<=1	C. coli
2014	14-CA01635	Broiler_caecum_content	EUCAMP2	0,5	2	4	<=0,125	<=0,5	<=1	C. jejuni
2014	14-CA01636	Broiler_caecum_content	EUCAMP2	1	2	4	0,25	<=0,5	<=1	C. jejuni
2014	14-CA01637	Broiler_caecum_content	EUCAMP2	<=0,125	<=0,25	16	4	16	<=1	C. jejuni
2014	14-CA01638	Broiler_caecum_content	EUCAMP2	1	2	>64	16	<=0,5	<=1	C. coli
2014	14-CA01639	Broiler_caecum_content	EUCAMP2	0,5	1	64	16	64	<=1	C. jejuni
2014	14-CA01640	Broiler_caecum_content	EUCAMP2	<=0,125	<=0,25	8	8	16	<=1	C. jejuni
2014	14-CA01641	Turkey_caecum_content	EUCAMP2	1	2	>64	16	<=0,5	<=1	C. coli
2014	14-CA01642	Turkey_caecum_content	EUCAMP2	1	2	>64	16	<=0,5	<=1	C. jejuni
2014	14-CA01643	Turkey_caecum_content	EUCAMP2	0,5	>16	>64	>16	>64	2	C. coli
2014	14-CA01644	Broiler_caecum_content	EUCAMP2	1	2	4	<=0,125	<=0,5	<=1	C. jejuni
2014	14-CA01645	Broiler_caecum_content	EUCAMP2	0,5	1	4	<=0,125	<=0,5	<=1	C. jejuni
2014	14-CA01646	Broiler_caecum_content	EUCAMP2	0,5	1	>64	>16	>64	<=1	C. jejuni
2014	14-CA01647	Broiler_caecum_content	EUCAMP2	0,5	1	64	4	<=0,5	<=1	C. coli
2014	14-CA01648	Turkey_caecum_content	EUCAMP2	0,5	>16	64	>16	64	>128	C. coli

Table S5: Minimum inhibitory concentrations of 6 antimicrobials for isolats of C. jejuni and C. coli from poultry

Year	Sample No.	Matrix_final	Antimicrobial panel	GEN	STR	NAL	CIP	TET	ERY	Species
2014	14-CA01649	Turkey_caecum_content	EUCAMP2	0,5	2	<=1	<=0,125	16	<=1	C. jejuni
2014	14-CA01650	Turkey_caecum_content	EUCAMP2	0,5	2	>64	>16	>64	2	C. coli
2014	14-CA01651	Turkey_caecum_content	EUCAMP2	0,5	2	64	16	64	<=1	C. coli
2014	14-CA01652	Turkey_caecum_content	EUCAMP2	0,5	2	64	8	<=0,5	<=1	C. coli
2014	14-CA01653	Turkey_caecum_content	EUCAMP2	0,5	1	64	16	64	>128	C. coli
2014	14-CA01654	Turkey_caecum_content	EUCAMP2	0,5	4	32	8	64	<=1	C. coli
2014	14-CA01655	Turkey_caecum_content	EUCAMP2	1	2	64	>16	>64	>128	C. coli
2014	14-CA01656	Turkey_caecum_content	EUCAMP2	1	4	64	16	64	<=1	C. coli
2014	14-CA01657	Turkey_caecum_content	EUCAMP2	0,5	1	32	8	<=0,5	<=1	C. coli
2014	14-CA01658	Turkey_caecum_content	EUCAMP2	0,5	1	32	8	<=0,5	<=1	C. coli
2014	14-CA01659	Turkey_caecum_content	EUCAMP2	1	4	64	16	64	<=1	C. coli
2014	14-CA01660	Turkey_caecum_content	EUCAMP2	0,5	1	64	16	64	<=1	C. coli
2014	14-CA01661	Turkey_caecum_content	EUCAMP2	0,5	1	>64	16	64	<=1	C. coli
2014	14-CA01662	Turkey_caecum_content	EUCAMP2	0,5	0,5	4	<=0,125	<=0,5	<=1	C. jejuni
2014	14-CA01663	Turkey_caecum_content	EUCAMP2	0,5	1	64	16	>64	>128	C. coli
2014	14-CA01664	Turkey_caecum_content	EUCAMP2	1	2	>64	>16	>64	2	C. coli
2014	14-CA01665	Turkey_caecum_content	EUCAMP2	0,5	1	64	>16	32	<=1	C. jejuni
2014	14-CA01666	Turkey_caecum_content	EUCAMP2	1	2	>64	>16	>64	2	C. coli
2014	14-CA01667	Turkey_caecum_content	EUCAMP2	0,5	2	64	>16	>64	>128	C. coli
2014	14-CA01668	Turkey_caecum_content	EUCAMP2	0,25	1	64	>16	>64	<=1	C. coli
2014	14-CA01669	Broiler_caecum_content	EUCAMP2	1	2	64	8	<=0,5	<=1	C. coli
2014	14-CA01670	Broiler_caecum_content	EUCAMP2	0,5	1	4	<=0,125	<=0,5	<=1	C. jejuni
2014	14-CA01671	Broiler_caecum_content	EUCAMP2	0,5	1	64	16	32	<=1	C. coli
2014	14-CA01672	Broiler_caecum_content	EUCAMP2	0,5	2	64	16	64	<=1	C. coli
2014	14-CA01673	Broiler_caecum_content	EUCAMP2	0,5	1	64	16	32	<=1	C. coli
2014	14-CA01674	Broiler_caecum_content	EUCAMP2	0,25	1	64	16	64	<=1	C. coli
2014	14-CA01675	Turkey_caecum_content	EUCAMP2	0,25	0,5	64	>16	>64	<=1	C. coli
2014	14-CA01677	Turkey_caecum_content	EUCAMP2	0,25	1	32	16	64	>128	C. coli
2014	14-CA01678	Turkey_caecum_content	EUCAMP2	0,5	1	32	16	64	>128	C. coli
2014	14-CA01679	Turkey_caecum_content	EUCAMP2	0,5	1	64	>16	>64	>128	C. coli
2014	14-CA01680	Broiler_caecum_content	EUCAMP2	0,5	2	64	16	>64	2	C. coli
2014	14-CA01681	Broiler_caecum_content	EUCAMP2	0,5	1	64	16	>64	<=1	C. coli

Table S5: Minimum inhibitory concentrations of 6 antimicrobials for isolats of C. jejuni and C. coli from poultry

Year	Sample No.	Matrix_final	Antimicrobial panel	GEN	STR	NAL	CIP	TET	ERY	Species
2014	14-CA01682	Broiler_caecum_content	EUCAMP2	1	4	>64	16	<=0,5	<=1	C. jejuni
2014	14-CA01683	Turkey_caecum_content	EUCAMP2	0,25	1	64	16	64	<=1	C. coli
2014	14-CA01684	Turkey_caecum_content	EUCAMP2	0,5	1	64	16	64	<=1	C. coli
2014	14-CA01685	Turkey_caecum_content	EUCAMP2	0,5	2	64	8	32	<=1	C. coli
2014	14-CA01686	Turkey_caecum_content	EUCAMP2	0,5	1	64	16	>64	<=1	C. coli
2014	14-CA01687	Broiler_caecum_content	EUCAMP2	0,5	2	<=1	16	64	<=1	C. jejuni
2014	14-CA01688	Broiler_caecum_content	EUCAMP2	0,5	2	<=1	16	>64	<=1	C. jejuni
2014	14-CA01689	Turkey_caecum_content	EUCAMP2	0,5	1	4	0,25	>64	<=1	C. jejuni
2014	14-CA01690	Broiler_caecum_content	EUCAMP2	0,5	1	>64	16	<=0,5	<=1	C. jejuni
2014	14-CA01691	Broiler_caecum_content	EUCAMP2	0,5	2	4	<=0,125	64	<=1	C. coli
2014	14-CA01695	Turkey_caecum_content	EUCAMP2	0,5	2	2	>16	>64	<=1	C. jejuni
2014	14-CA01696	Turkey_caecum_content	EUCAMP2	0,5	4	>64	16	>64	<=1	C. coli
2014	14-CA01697	Turkey_caecum_content	EUCAMP2	0,5	1	>64	16	64	<=1	C. coli
2014	14-CA01698	Turkey_caecum_content	EUCAMP2	0,5	1	64	16	<=0,5	<=1	C. coli
2014	14-CA01699	Turkey_caecum_content	EUCAMP2	0,5	2	64	16	>64	2	C. coli
2014	14-CA01700	Turkey_caecum_content	EUCAMP2	0,5	2	64	16	>64	2	C. coli
2014	14-CA01701	Turkey_caecum_content	EUCAMP2	0,5	2	64	8	32	<=1	C. coli
2014	14-CA01720	Broiler_caecum_content	EUCAMP2	1	4	4	<=0,125	64	<=1	C. coli
2014	14-CA01727	Broiler_caecum_content	EUCAMP2	<=0,125	<=0,25	2	<=0,125	<=0,5	<=1	C. jejuni
2014	14-CA01733	Turkey_caecum_content	EUCAMP2	0,25	1	64	16	<=0,5	<=1	C. coli
2014	14-CA01734	Turkey_caecum_content	EUCAMP2	0,25	1	64	16	64	<=1	C. coli
2014	14-CA01735	Turkey_caecum_content	EUCAMP2	0,25	1	<=1	16	<=0,5	<=1	C. jejuni
2014	14-CA01736	Turkey_caecum_content	EUCAMP2	0,25	1	32	>16	64	>128	C. coli
2014	14-CA01737	Turkey_caecum_content	EUCAMP2	0,5	1	64	16	64	>128	C. coli
2014	14-CA01738	Turkey_caecum_content	EUCAMP2	0,5	1	64	16	64	<=1	C. jejuni
2014	14-CA01739	Turkey_caecum_content	EUCAMP2	0,25	1	>64	>16	>64	<=1	C. coli
2014	14-CA01776	Broiler_caecum_content	EUCAMP2	0,5	2	64	>16	>64	<=1	C. coli
2014	14-CA01777	Turkey_caecum_content	EUCAMP2	1	2	>64	16	<=0,5	<=1	C. jejuni
2014	14-CA01778	Turkey_caecum_content	EUCAMP2	0,5	4	64	8	64	<=1	C. coli
2014	14-CA01779	Turkey_caecum_content	EUCAMP2	0,5	2	4	<=0,125	<=0,5	<=1	C. jejuni
2014	14-CA01780	Turkey_caecum_content	EUCAMP2	0,5	2	32	>16	>64	<=1	C. coli
2014	14-CA01781	Turkey_caecum_content	EUCAMP2	0,5	1	32	8	32	<=1	C. coli

Table S5: Minimum inhibitory concentrations of 6 antimicrobials for isolats of C. jejuni and C. coli from poultry

Year	Sample No.	Matrix_final	Antimicrobial panel	GEN	STR	NAL	CIP	TET	ERY	Species
2014	14-CA01782	Turkey_caecum_content	EUCAMP2	0,5	2	64	8	64	<=1	C. coli
2014	14-CA01783	Turkey_caecum_content	EUCAMP2	0,5	1	4	<=0,125	64	<=1	C. jejuni
2014	14-CA01794	Turkey_caecum_content	EUCAMP2	0,25	1	64	>16	>64	<=1	C. jejuni
2014	14-CA01795	Turkey_caecum_content	EUCAMP2	0,5	2	32	16	<=0,5	<=1	C. jejuni
2014	14-CA01796	Turkey_caecum_content	EUCAMP2	0,5	2	>64	>16	>64	2	C. coli
2014	14-CA01797	Turkey_caecum_content	EUCAMP2	0,25	0,5	4	0,25	64	<=1	C. jejuni
2014	14-CA01804	Broiler_caecum_content	EUCAMP2	0,5	2	2	16	<=0,5	<=1	C. jejuni
2014	14-CA01815	Turkey_caecum_content	EUCAMP2	0,5	1	4	0,25	<=0,5	<=1	C. jejuni
2014	14-CA01816	Broiler_caecum_content	EUCAMP2	0,5	0,5	32	>16	>64	<=1	C. coli
2014	14-CA01828	Turkey_caecum_content	EUCAMP2	0,5	1	64	>16	>64	>128	C. coli
2014	14-CA01829	Turkey_caecum_content	EUCAMP2	0,5	2	64	16	<=0,5	<=1	C. jejuni
2014	14-CA01830	Turkey_caecum_content	EUCAMP2	0,5	1	64	16	<=0,5	<=1	C. coli
2014	14-CA01831	Turkey_caecum_content	EUCAMP2	0,25	2	4	<=0,125	<=0,5	<=1	C. jejuni
2014	14-CA01832	Turkey_caecum_content	EUCAMP2	0,5	1	8	0,25	<=0,5	2	C. coli
2014	14-CA01833	Turkey_caecum_content	EUCAMP2	0,5	1	64	8	>64	>128	C. coli
2014	14-CA01834	Turkey_caecum_content	EUCAMP2	0,5	1	64	>16	64	>128	C. coli
2014	14-CA01835	Turkey_caecum_content	EUCAMP2	0,5	0,5	64	16	64	<=1	C. jejuni
2014	14-CA01836	Turkey_caecum_content	EUCAMP2	1	>16	>64	>16	>64	4	C. coli
2014	14-CA01837	Turkey_caecum_content	EUCAMP2	0,5	>16	32	>16	64	>128	C. coli
2014	14-CA01838	Turkey_caecum_content	EUCAMP2	0,5	2	32	>16	>64	<=1	C. jejuni
2014	14-CA01839	Turkey_caecum_content	EUCAMP2	0,25	1	32	>16	64	>128	C. coli
2014	14-CA01840	Turkey_caecum_content	EUCAMP2	0,25	2	32	>16	>64	>128	C. coli
2014	14-CA01841	Turkey_caecum_content	EUCAMP2	<=0,125	<=0,25	32	16	64	<=1	C. jejuni
2014	14-CA01842	Turkey_caecum_content	EUCAMP2	0,5	1	64	16	64	<=1	C. coli
2014	14-CA01843	Turkey_caecum_content	EUCAMP2	0,5	2	64	>16	64	>128	C. coli
2014	14-CA01844	Turkey_caecum_content	EUCAMP2	0,5	2	64	>16	>64	>128	C. coli
2014	14-CA01845	Turkey_caecum_content	EUCAMP2	0,25	1	32	>16	>64	>128	C. coli
2014	14-CA01846	Turkey_caecum_content	EUCAMP2	0,25	>16	64	16	64	>128	C. coli
2014	14-CA01847	Turkey_caecum_content	EUCAMP2	1	2	2	16	>64	<=1	C. jejuni
2014	14-CA01848	Turkey_caecum_content	EUCAMP2	0,5	1	64	>16	>64	<=1	C. coli
2014	14-CA01849	Turkey_caecum_content	EUCAMP2	0,5	2	64	>16	>64	>128	C. coli
2014	14-CA01850	Turkey_caecum_content	EUCAMP2	0,5	1	8	0,5	>64	<=1	C. coli

Table S5: Minimum inhibitory concentrations of 6 antimicrobials for isolats of C. jejuni and C. coli from poultry

Year	Sample No.	Matrix_final	Antimicrobial panel	GEN	STR	NAL	CIP	TET	ERY	Species
2014	14-CA01850	Turkey_caecum_content	EUCAMP2	1	2	4	<=0,125	<=0,5	<=1	C. jejuni
2014	14-CA01851	Turkey_caecum_content	EUCAMP2	0,25	1	64	>16	>64	<=1	C. jejuni
2014	14-CA01852	Turkey_caecum_content	EUCAMP2	1	2	64	16	>64	2	C. coli
2014	14-CA01853	Turkey_caecum_content	EUCAMP2	0,25	1	64	16	32	<=1	C. coli
2014	14-CA01854	Turkey_caecum_content	EUCAMP2	0,5	2	64	16	>64	<=1	C. coli
2014	14-CA01855	Turkey_caecum_content	EUCAMP2	0,5	2	64	8	>64	<=1	C. coli
2014	14-CA01856	Turkey_caecum_content	EUCAMP2	0,5	1	32	8	>64	>128	C. coli
2014	14-CA01857	Turkey_caecum_content	EUCAMP2	0,5	2	4	<=0,125	<=0,5	<=1	C. coli
2014	14-CA01858	Turkey_caecum_content	EUCAMP2	0,5	2	4	<=0,125	<=0,5	<=1	C. coli
2014	14-CA01859	Turkey_caecum_content	EUCAMP2	0,5	1	64	>16	32	<=1	C. jejuni
2014	14-CA01860	Turkey_caecum_content	EUCAMP2	0,5	1	64	>16	>64	<=1	C. jejuni
2014	14-CA01861	Turkey_caecum_content	EUCAMP2	0,25	1	32	16	32	<=1	C. coli
2014	14-CA01862	Turkey_caecum_content	EUCAMP2	<=0,125	0,5	8	4	16	<=1	C. jejuni
2014	14-CA01863	Turkey_caecum_content	EUCAMP2	1	1	64	16	<=0,5	<=1	C. jejuni
2014	14-CA01864	Turkey_caecum_content	EUCAMP2	0,25	0,5	64	16	16	<=1	C. jejuni
2014	14-CA01865	Turkey_caecum_content	EUCAMP2	0,5	1	64	16	64	<=1	C. jejuni
2014	14-CA01876	Broiler_caecum_content	EUCAMP2	0,5	1	64	16	64	<=1	C. jejuni
2014	14-CA01881	Turkey_caecum_content	EUCAMP2	0,5	2	64	16	<=0,5	<=1	C. jejuni
2014	14-CA01882	Turkey_caecum_content	EUCAMP2	1	2	4	0,25	<=0,5	<=1	C. jejuni
2014	14-CA01883	Turkey_caecum_content	EUCAMP2	0,25	0,5	32	16	64	<=1	C. jejuni
2014	15-CA00010	Turkey_caecum_content	EUCAMP2	0,5	2	64	>16	>64	2	C. coli
2014	15-CA00011	Broiler_caecum_content	EUCAMP2	0,25	1	32	>16	<=0,5	<=1	C. jejuni
2014	15-CA00012	Broiler_caecum_content	EUCAMP2	<=0,125	0,5	64	>16	32	<=1	C. jejuni
2014	15-CA00013	Broiler_caecum_content	EUCAMP2	0,5	1	64	16	32	>128	C. coli
2014	15-CA00014	Broiler_caecum_content	EUCAMP2	0,5	1	64	8	64	<=1	C. jejuni
2014	15-CA00041	Broiler_caecum_content	EUCAMP2	1	2	4	<=0,125	<=0,5	<=1	C. jejuni
2014	15-CA00042	Broiler_caecum_content	EUCAMP2	0,5	2	64	16	16	<=1	C. jejuni
2014	15-CA00043	Broiler_caecum_content	EUCAMP2	0,5	1	4	<=0,125	<=0,5	<=1	C. jejuni
2014	15-CA00044	Broiler_caecum_content	EUCAMP2	1	2	64	16	>64	<=1	C. coli
2014	15-CA00045	Turkey_caecum_content	EUCAMP2	0,5	0,5	8	0,25	<=0,5	<=1	C. jejuni
2014	15-CA00046	Turkey_caecum_content	EUCAMP2	0,25	0,5	64	16	64	<=1	C. coli
2014	15-CA00047	Turkey_caecum_content	EUCAMP2	0,25	2	64	8	64	<=1	C. coli

Table S5: Minimum inhibitory concentrations of 6 antimicrobials for isolats of C. jejuni and C. coli from poultry

Year	Sample No.	Matrix_final	Antimicrobial panel	GEN	STR	NAL	CIP	TET	ERY	Species
2014	15-CA00048	Turkey_caecum_content	EUCAMP2	0,25	0,5	64	>16	64	<=1	C. jejuni
2014	15-CA00049	Turkey_caecum_content	EUCAMP2	0,5	2	64	8	64	<=1	C. coli
2016	16-CA00122	Broiler_caecum_content	EUCAMP2	0,25	1	4	<=0,125	<=0,5	<=1	C. jejuni
2016	16-CA00132	Turkey_caecum_content	EUCAMP2	1	>16	>64	>16	>64	>128	C. coli
2016	16-CA00143	Turkey_caecum_content	EUCAMP2	0,5	2	64	>16	8	<=1	C. jejuni
2016	16-CA00228	Turkey_caecum_content	EUCAMP2	0,25	2	32	16	64	>128	C. coli
2016	16-CA00229	Turkey_caecum_content	EUCAMP2	1	2	64	8	64	<=1	C. coli
2016	16-CA00230	Broiler_caecum_content	EUCAMP2	0,25	1	64	>16	<=0,5	<=1	C. jejuni
2016	16-CA00231	Broiler_caecum_content	EUCAMP2	0,5	2	4	0,25	<=0,5	<=1	C. jejuni
2016	16-CA00241	Turkey_caecum_content	EUCAMP2	0,25	0,5	16	8	16	<=1	C. jejuni
2016	16-CA00242	Turkey_caecum_content	EUCAMP2	0,25	2	32	>16	64	2	C. coli
2016	16-CA00243	Broiler_caecum_content	EUCAMP2	0,5	2	32	16	32	<=1	C. jejuni
2016	16-CA00250	Broiler_caecum_content	EUCAMP2	0,5	2	64	16	<=0,5	<=1	C. jejuni
2016	16-CA00251	Broiler_caecum_content	EUCAMP2	0,5	2	64	16	<=0,5	<=1	C. jejuni
2016	16-CA00268	Turkey_caecum_content	EUCAMP2	0,25	1	32	16	<=0,5	<=1	C. coli
2016	16-CA00276	Turkey_caecum_content	EUCAMP2	0,5	1	32	16	64	<=1	C. jejuni
2016	16-CA00277	Turkey_caecum_content	EUCAMP2	0,5	2	32	>16	<=0,5	<=1	C. jejuni
2016	16-CA00278	Turkey_caecum_content	EUCAMP2	0,25	1	64	>16	>64	2	C. jejuni
2016	16-CA00279	Turkey_caecum_content	EUCAMP2	0,5	2	64	16	64	<=1	C. coli
2016	16-CA00280	Broiler_caecum_content	EUCAMP2	0,5	2	2	<=0,125	<=0,5	<=1	C. jejuni
2016	16-CA00282	Turkey_caecum_content	EUCAMP2	0,5	2	4	0,25	<=0,5	<=1	C. jejuni
2016	16-CA00283	Turkey_caecum_content	EUCAMP2	0,5	2	64	>16	>64	2	C. coli
2016	16-CA00284	Broiler_caecum_content	EUCAMP2	0,5	2	64	16	<=0,5	<=1	C. jejuni
2016	16-CA00286	Turkey_caecum_content	EUCAMP2	0,25	>16	32	>16	>64	>128	C. coli
2016	16-CA00287	Turkey_caecum_content	EUCAMP2	0,5	1	64	16	>64	<=1	C. jejuni
2016	16-CA00288	Turkey_caecum_content	EUCAMP2	0,5	1	64	>16	>64	<=1	C. jejuni
2016	16-CA00289	Turkey_caecum_content	EUCAMP2	0,5	1	64	>16	>64	<=1	C. jejuni
2016	16-CA00290	Turkey_caecum_content	EUCAMP2	0,5	2	32	8	32	<=1	C. coli
2016	16-CA00291	Turkey_caecum_content	EUCAMP2	0,25	2	32	16	>64	2	C. coli
2016	16-CA00292	Turkey_caecum_content	EUCAMP2	0,25	2	32	>16	64	<=1	C. coli
2016	16-CA00293	Turkey_caecum_content	EUCAMP2	0,25	>16	64	>16	>64	>128	C. coli
2016	16-CA00294	Turkey_caecum_content	EUCAMP2	0,5	1	64	16	<=0,5	<=1	C. jejuni

Table S5: Minimum inhibitory concentrations of 6 antimicrobials for isolats of C. jejuni and C. coli from poultry

Year	Sample No.	Matrix_final	Antimicrobial panel	GEN	STR	NAL	CIP	TET	ERY	Species
2016	16-CA00331	Broiler_caecum_content	EUCAMP2	0,5	1	64	16	<=0,5	<=1	C. jejuni
2016	16-CA00332	Broiler_caecum_content	EUCAMP2	0,5	1	32	8	<=0,5	<=1	C. jejuni
2016	16-CA00339	Broiler_caecum_content	EUCAMP2	0,5	2	>64	16	16	2	C. jejuni
2016	16-CA00340	Broiler_caecum_content	EUCAMP2	1	8	32	16	<=0,5	<=1	C. jejuni
2016	16-CA00341	Broiler_caecum_content	EUCAMP2	0,5	2	64	16	64	<=1	C. jejuni
2016	16-CA00344	Turkey_caecum_content	EUCAMP2	0,5	1	2	16	<=0,5	<=1	C. jejuni
2016	16-CA00345	Broiler_caecum_content	EUCAMP2	0,5	1	32	16	32	<=1	C. jejuni
2016	16-CA00346	Broiler_caecum_content	EUCAMP2	0,5	1	32	16	32	<=1	C. jejuni
2016	16-CA00368	Turkey_caecum_content	EUCAMP2	0,25	1	32	>16	<=0,5	<=1	C. coli
2016	16-CA00370	Broiler_caecum_content	EUCAMP2	0,25	1	32	16	<=0,5	<=1	C. jejuni
2016	16-CA00371	Broiler_caecum_content	EUCAMP2	1	2	>64	16	<=0,5	<=1	C. jejuni
2016	16-CA00372	Turkey_caecum_content	EUCAMP2	0,5	2	64	16	<=0,5	<=1	C. jejuni
2016	16-CA00373	Broiler_caecum_content	EUCAMP2	0,25	1	64	>16	<=0,5	<=1	C. jejuni
2016	16-CA00377	Broiler_caecum_content	EUCAMP2	0,25	1	4	0,25	<=0,5	2	C. jejuni
2016	16-CA00390	Broiler_caecum_content	EUCAMP2	0,25	<=0,25	4	0,25	<=0,5	<=1	C. jejuni
2016	16-CA00403	Turkey_caecum_content	EUCAMP2	0,5	1	32	8	<=0,5	<=1	C. coli
2016	16-CA00410	Turkey_caecum_content	EUCAMP2	0,5	1	64	>16	>64	<=1	C. jejuni
2016	16-CA00411	Turkey_caecum_content	EUCAMP2	0,5	2	64	>16	64	2	C. coli
2016	16-CA00412	Turkey_caecum_content	EUCAMP2	0,25	0,5	4	<=0,125	<=0,5	<=1	C. jejuni
2016	16-CA00413	Turkey_caecum_content	EUCAMP2	1	2	64	16	64	2	C. coli
2016	16-CA00414	Turkey_caecum_content	EUCAMP2	0,25	2	32	16	64	<=1	C. coli
2016	16-CA00415	Turkey_caecum_content	EUCAMP2	1	8	2	>16	>64	<=1	C. jejuni
2016	16-CA00416	Turkey_caecum_content	EUCAMP2	0,25	1	32	>16	>64	<=1	C. jejuni
2016	16-CA00417	Turkey_caecum_content	EUCAMP2	0,5	2	64	16	>64	<=1	C. coli
2016	16-CA00418	Turkey_caecum_content	EUCAMP2	0,5	1	>64	16	<=0,5	<=1	C. jejuni
2016	16-CA00419	Turkey_caecum_content	EUCAMP2	1	>16	64	16	<=0,5	<=1	C. jejuni
2016	16-CA00420	Turkey_caecum_content	EUCAMP2	0,5	2	4	0,25	<=0,5	<=1	C. jejuni
2016	16-CA00421	Turkey_caecum_content	EUCAMP2	0,5	2	64	16	64	<=1	C. coli
2016	16-CA00422	Turkey_caecum_content	EUCAMP2	0,5	1	>64	>16	>64	<=1	C. coli
2016	16-CA00423	Turkey_caecum_content	EUCAMP2	0,5	1	>64	16	<=0,5	<=1	C. jejuni
2016	16-CA00424	Turkey_caecum_content	EUCAMP2	0,5	2	>64	>16	64	<=1	C. coli
2016	16-CA00425	Turkey_caecum_content	EUCAMP2	0,5	2	64	16	<=0,5	<=1	C. jejuni

Table S5: Minimum inhibitory concentrations of 6 antimicrobials for isolats of C. jejuni and C. coli from poultry

Year	Sample No.	Matrix_final	Antimicrobial panel	GEN	STR	NAL	CIP	TET	ERY	Species
2016	16-CA00426	Turkey_caecum_content	EUCAMP2	0,25	1	32	16	64	<=1	C. coli
2016	16-CA00427	Turkey_caecum_content	EUCAMP2	1	2	64	16	64	<=1	C. coli
2016	16-CA00428	Turkey_caecum_content	EUCAMP2	0,25	1	64	16	32	<=1	C. coli
2016	16-CA00429	Broiler_caecum_content	EUCAMP2	0,5	2	>64	>16	>64	2	C. jejuni
2016	16-CA00430	Broiler_caecum_content	EUCAMP2	1	2	4	0,25	<=0,5	<=1	C. jejuni
2016	16-CA00431	Turkey_caecum_content	EUCAMP2	0,5	0,5	4	0,25	<=0,5	<=1	C. jejuni
2016	16-CA00432	Turkey_caecum_content	EUCAMP2	0,5	2	64	>16	>64	2	C. coli
2016	16-CA00433	Turkey_caecum_content	EUCAMP2	0,5	2	64	16	64	2	C. coli
2016	16-CA00434	Turkey_caecum_content	EUCAMP2	0,5	2	64	16	<=0,5	<=1	C. jejuni
2016	16-CA00435	Turkey_caecum_content	EUCAMP2	0,5	1	32	8	<=0,5	<=1	C. jejuni
2016	16-CA00436	Turkey_caecum_content	EUCAMP2	0,5	2	32	8	64	>128	C. coli
2016	16-CA00437	Turkey_caecum_content	EUCAMP2	0,5	1	32	4	<=0,5	<=1	C. coli
2016	16-CA00438	Turkey_caecum_content	EUCAMP2	0,5	1	>64	16	<=0,5	2	C. jejuni
2016	16-CA00439	Broiler_caecum_content	EUCAMP2	0,5	1	64	>16	>64	<=1	C. jejuni
2016	16-CA00440	Turkey_caecum_content	EUCAMP2	0,25	1	16	8	32	>128	C. coli
2016	16-CA00440	Turkey_caecum_content	EUCAMP2	0,5	>16	64	16	64	<=1	C. jejuni
2016	16-CA00441	Turkey_caecum_content	EUCAMP2	1	2	4	0,25	<=0,5	<=1	C. jejuni
2016	16-CA00442	Turkey_caecum_content	EUCAMP2	0,5	0,5	4	<=0,125	<=0,5	<=1	C. jejuni
2016	16-CA00443	Turkey_caecum_content	EUCAMP2	0,5	1	32	16	<=0,5	<=1	C. coli
2016	16-CA00444	Turkey_caecum_content	EUCAMP2	0,25	1	32	16	32	<=1	C. coli
2016	16-CA00445	Turkey_caecum_content	EUCAMP2	0,5	2	32	8	16	<=1	C. coli
2016	16-CA00446	Turkey_caecum_content	EUCAMP2	0,5	1	32	8	<=0,5	<=1	C. jejuni
2016	16-CA00447	Turkey_caecum_content	EUCAMP2	0,5	1	32	16	32	<=1	C. jejuni
2016	16-CA00448	Turkey_caecum_content	EUCAMP2	1	2	64	16	<=0,5	<=1	C. jejuni
2016	16-CA00452	Broiler_caecum_content	EUCAMP2	0,5	1	8	0,25	<=0,5	2	C. jejuni
2016	16-CA00453	Broiler_caecum_content	EUCAMP2	0,5	2	64	16	<=0,5	<=1	C. jejuni
2016	16-CA00461	Broiler_caecum_content	EUCAMP2	0,25	1	8	0,5	<=0,5	<=1	C. jejuni
2016	16-CA00469	Turkey_caecum_content	EUCAMP2	0,25	1	64	>16	>64	<=1	C. jejuni
2016	16-CA00476	Broiler_caecum_content	EUCAMP2	0,5	1	64	16	64	<=1	C. jejuni
2016	16-CA00477	Broiler_caecum_content	EUCAMP2	0,25	>16	32	>16	32	<=1	C. coli
2016	16-CA00482	Broiler_caecum_content	EUCAMP2	0,5	2	64	16	32	<=1	C. coli
2016	16-CA00496	Broiler_caecum_content	EUCAMP2	0,5	1	2	0,25	<=0,5	<=1	C. jejuni

Table S5: Minimum inhibitory concentrations of 6 antimicrobials for isolats of C. jejuni and C. coli from poultry

Year	Sample No.	Matrix_final	Antimicrobial panel	GEN	STR	NAL	CIP	TET	ERY	Species
2016	16-CA00502	Turkey_caecum_content	EUCAMP2	<=0,125	>16	2	<=0,125	4	<=1	C. coli
2016	16-CA00529	Turkey_caecum_content	EUCAMP2	0,5	1	32	16	<=0,5	<=1	C. jejuni
2016	16-CA00530	Turkey_caecum_content	EUCAMP2	0,5	1	64	>16	<=0,5	2	C. coli
2016	16-CA00531	Turkey_caecum_content	EUCAMP2	0,5	2	64	>16	32	4	C. coli
2016	16-CA00532	Turkey_caecum_content	EUCAMP2	0,5	1	64	16	32	<=1	C. coli
2016	16-CA00533	Turkey_caecum_content	EUCAMP2	0,5	1	32	>16	64	<=1	C. coli
2016	16-CA00534	Turkey_caecum_content	EUCAMP2	0,5	1	4	0,25	<=0,5	<=1	C. jejuni
2016	16-CA00535	Turkey_caecum_content	EUCAMP2	0,25	1	32	16	16	<=1	C. coli
2016	16-CA00536	Turkey_caecum_content	EUCAMP2	0,5	2	64	16	64	<=1	C. coli
2016	16-CA00537	Turkey_caecum_content	EUCAMP2	0,5	1	32	16	64	>128	C. coli
2016	16-CA00538	Turkey_caecum_content	EUCAMP2	0,5	1	32	16	32	<=1	C. jejuni
2016	16-CA00539	Turkey_caecum_content	EUCAMP2	0,5	1	64	>16	<=0,5	<=1	C. jejuni
2016	16-CA00540	Turkey_caecum_content	EUCAMP2	0,25	1	32	>16	64	>128	C. coli
2016	16-CA00541	Turkey_caecum_content	EUCAMP2	0,5	2	64	16	<=0,5	<=1	C. jejuni
2016	16-CA00542	Turkey_caecum_content	EUCAMP2	0,5	1	64	>16	>64	<=1	C. jejuni
2016	16-CA00543	Turkey_caecum_content	EUCAMP2	0,5	1	32	16	64	2	C. coli
2016	16-CA00544	Turkey_caecum_content	EUCAMP2	0,25	2	16	>16	64	4	C. coli
2016	16-CA00545	Turkey_caecum_content	EUCAMP2	0,25	2	8	16	32	2	C. coli
2016	16-CA00546	Turkey_caecum_content	EUCAMP2	0,5	>16	64	16	64	>128	C. coli
2016	16-CA00547	Turkey_caecum_content	EUCAMP2	0,25	1	64	>16	<=0,5	<=1	C. jejuni
2016	16-CA00548	Turkey_caecum_content	EUCAMP2	0,25	1	64	16	32	<=1	C. coli
2016	16-CA00549	Turkey_caecum_content	EUCAMP2	0,25	1	64	16	32	<=1	C. coli
2016	16-CA00550	Turkey_caecum_content	EUCAMP2	0,25	1	64	16	32	<=1	C. coli
2016	16-CA00551	Turkey_caecum_content	EUCAMP2	0,5	1	64	>16	>64	<=1	C. jejuni
2016	16-CA00552	Turkey_caecum_content	EUCAMP2	<=0,125	1	32	4	8	<=1	C. coli
2016	16-CA00553	Turkey_caecum_content	EUCAMP2	0,5	1	32	16	32	<=1	C. coli
2016	16-CA00554	Turkey_caecum_content	EUCAMP2	0,5	1	>64	>16	<=0,5	2	C. jejuni
2016	16-CA00555	Turkey_caecum_content	EUCAMP2	0,5	2	>64	>16	<=0,5	<=1	C. jejuni
2016	16-CA00557	Turkey_caecum_content	EUCAMP2	0,5	1	64	>16	<=0,5	<=1	C. jejuni
2016	16-CA00558	Broiler_caecum_content	EUCAMP2	0,5	>16	4	<=0,125	8	<=1	C. coli
2016	16-CA00559	Broiler_caecum_content	EUCAMP2	0,25	1	32	>16	64	<=1	C. jejuni
2016	16-CA00560	Turkey_caecum_content	EUCAMP2	0,25	1	16	4	16	>128	C. coli

Table S5: Minimum inhibitory concentrations of 6 antimicrobials for isolats of C. jejuni and C. coli from poultry

Year	Sample No.	Matrix_final	Antimicrobial panel	GEN	STR	NAL	CIP	TET	ERY	Species
2016	16-CA00561	Turkey_caecum_content	EUCAMP2	0,5	2	32	16	64	2	C. coli
2016	16-CA00581	Turkey_caecum_content	EUCAMP2	<=0,125	1	4	0,25	<=0,5	<=1	C. coli
2016	16-CA00588	Turkey_caecum_content	EUCAMP2	0,25	2	32	8	64	<=1	C. coli
2016	16-CA00590	Broiler_caecum_content	EUCAMP2	1	4	32	16	<=0,5	<=1	C. jejuni
2016	16-CA00598	Turkey_caecum_content	EUCAMP2	0,25	0,5	<=1	8	<=0,5	<=1	C. jejuni
2016	16-CA00609	Turkey_caecum_content	EUCAMP2	0,5	1	64	16	64	<=1	C. jejuni
2016	16-CA00610	Turkey_caecum_content	EUCAMP2	0,5	1	64	16	>64	<=1	C. jejuni
2016	16-CA00619	Broiler_caecum_content	EUCAMP2	0,5	2	64	>16	>64	2	C. jejuni
2016	16-CA00620	Broiler_caecum_content	EUCAMP2	0,5	1	4	<=0,125	<=0,5	<=1	C. jejuni
2016	16-CA00621	Broiler_caecum_content	EUCAMP2	0,5	1	4	0,25	<=0,5	<=1	C. jejuni
2016	16-CA00639	Turkey_caecum_content	EUCAMP2	0,5	1	32	16	>64	>128	C. coli
2016	16-CA00640	Turkey_caecum_content	EUCAMP2	0,25	1	32	>16	16	2	C. coli
2016	16-CA00641	Turkey_caecum_content	EUCAMP2	0,5	1	32	16	64	2	C. coli
2016	16-CA00641	Turkey_caecum_content	EUCAMP2	0,5	1	>64	>16	<=0,5	<=1	C. jejuni
2016	16-CA00642	Turkey_caecum_content	EUCAMP2	0,5	1	32	16	64	4	C. coli
2016	16-CA00643	Turkey_caecum_content	EUCAMP2	0,5	2	4	0,25	<=0,5	<=1	C. jejuni
2016	16-CA00644	Broiler_caecum_content	EUCAMP2	0,25	1	16	16	32	<=1	C. jejuni
2016	16-CA00645	Turkey_caecum_content	EUCAMP2	0,25	1	64	>16	<=0,5	<=1	C. jejuni
2016	16-CA00646	Turkey_caecum_content	EUCAMP2	0,5	1	64	16	<=0,5	<=1	C. coli
2016	16-CA00647	Turkey_caecum_content	EUCAMP2	0,5	1	64	16	<=0,5	<=1	C. coli
2016	16-CA00648	Turkey_caecum_content	EUCAMP2	0,25	0,5	64	>16	<=0,5	<=1	C. jejuni
2016	16-CA00649	Turkey_caecum_content	EUCAMP2	0,5	2	64	8	<=0,5	<=1	C. coli
2016	16-CA00650	Turkey_caecum_content	EUCAMP2	0,5	2	64	>16	64	4	C. coli
2016	16-CA00651	Turkey_caecum_content	EUCAMP2	0,5	2	32	0,5	<=0,5	<=1	C. jejuni
2016	16-CA00652	Turkey_caecum_content	EUCAMP2	0,5	2	32	8	<=0,5	<=1	C. coli
2016	16-CA00653	Turkey_caecum_content	EUCAMP2	0,25	1	32	16	32	<=1	C. coli
2016	16-CA00654	Turkey_caecum_content	EUCAMP2	0,25	1	64	16	32	<=1	C. coli
2016	16-CA00655	Broiler_caecum_content	EUCAMP2	0,5	1	32	>16	64	4	C. coli
2016	16-CA00656	Broiler_caecum_content	EUCAMP2	0,5	2	4	0,25	<=0,5	<=1	C. jejuni
2016	16-CA00662	Turkey_caecum_content	EUCAMP2	0,5	>16	4	0,25	<=0,5	2	C. coli
2016	16-CA00662	Turkey_caecum_content	EUCAMP2	0,5	1	64	>16	<=0,5	2	C. jejuni
2016	16-CA00663	Turkey_caecum_content	EUCAMP2	0,5	2	2	>16	<=0,5	<=1	C. jejuni

Table S5: Minimum inhibitory concentrations of 6 antimicrobials for isolats of C. jejuni and C. coli from poultry

Year	Sample No.	Matrix_final	Antimicrobial panel	GEN	STR	NAL	CIP	TET	ERY	Species
2016	16-CA00664	Turkey_caecum_content	EUCAMP2	0,5	2	64	>16	<=0,5	2	C. coli
2016	16-CA00665	Turkey_caecum_content	EUCAMP2	0,5	1	32	16	<=0,5	<=1	C. coli
2016	16-CA00700	Turkey_caecum_content	EUCAMP2	<=0,125	16	32	8	<=0,5	<=1	C. jejuni
2016	16-CA00701	Broiler_caecum_content	EUCAMP2	0,25	1	4	0,5	<=0,5	<=1	C. jejuni
2016	16-CA00703	Turkey_caecum_content	EUCAMP2	<=0,125	0,5	4	<=0,125	<=0,5	<=1	C. jejuni
2016	16-CA00704	Turkey_caecum_content	EUCAMP2	0,25	1	32	16	64	<=1	C. jejuni
2016	16-CA00713	Broiler_caecum_content	EUCAMP2	0,5	1	64	16	<=0,5	<=1	C. jejuni
2016	16-CA00714	Broiler_caecum_content	EUCAMP2	<=0,125	<=0,25	32	16	32	<=1	C. jejuni
2016	16-CA00732	Broiler_caecum_content	EUCAMP2	0,25	1	32	16	64	2	C. coli
2016	16-CA00743	Broiler_caecum_content	EUCAMP2	0,25	1	32	16	64	<=1	C. jejuni
2016	16-CA00744	Broiler_caecum_content	EUCAMP2	0,25	1	32	>16	64	<=1	C. coli
2016	16-CA00774	Broiler_caecum_content	EUCAMP2	1	2	4	0,25	<=0,5	<=1	C. jejuni
2016	16-CA00775	Broiler_caecum_content	EUCAMP2	0,5	1	64	16	>64	<=1	C. jejuni
2016	16-CA00777	Broiler_caecum_content	EUCAMP2	0,5	1	64	16	<=0,5	<=1	C. jejuni
2016	16-CA00780	Broiler_caecum_content	EUCAMP2	1	2	4	<=0,125	<=0,5	<=1	C. coli
2016	16-CA00781	Broiler_caecum_content	EUCAMP2	0,25	0,5	64	>16	<=0,5	<=1	C. jejuni
2016	16-CA00783	Broiler_caecum_content	EUCAMP2	0,5	1	32	>16	32	<=1	C. jejuni
2016	16-CA00794	Broiler_caecum_content	EUCAMP2	0,5	2	64	>16	>64	<=1	C. jejuni
2016	16-CA00795	Broiler_caecum_content	EUCAMP2	0,5	2	64	>16	>64	2	C. coli
2016	16-CA00797	Turkey_caecum_content	EUCAMP2	<=0,125	0,5	32	>16	64	2	C. jejuni
2016	16-CA00798	Turkey_caecum_content	EUCAMP2	0,25	0,5	32	16	64	<=1	C. coli
2016	16-CA00799	Turkey_caecum_content	EUCAMP2	0,5	2	64	16	<=0,5	<=1	C. jejuni
2016	16-CA00800	Turkey_caecum_content	EUCAMP2	0,25	1	64	>16	<=0,5	<=1	C. jejuni
2016	16-CA00801	Turkey_caecum_content	EUCAMP2	0,5	2	64	16	<=0,5	<=1	C. jejuni
2016	16-CA00802	Turkey_caecum_content	EUCAMP2	0,25	1	32	16	16	<=1	C. jejuni
2016	16-CA00803	Turkey_caecum_content	EUCAMP2	0,25	0,5	32	>16	>64	<=1	C. jejuni
2016	16-CA00804	Turkey_caecum_content	EUCAMP2	<=0,125	<=0,25	32	8	32	<=1	C. jejuni
2016	16-CA00805	Turkey_caecum_content	EUCAMP2	1	2	64	16	<=0,5	<=1	C. jejuni
2016	16-CA00806	Turkey_caecum_content	EUCAMP2	0,5	2	4	<=0,125	<=0,5	<=1	C. jejuni
2016	16-CA00807	Turkey_caecum_content	EUCAMP2	0,25	0,5	32	16	64	<=1	C. coli
2016	16-CA00808	Broiler_caecum_content	EUCAMP2	0,25	1	64	>16	>64	<=1	C. jejuni
2016	16-CA00809	Broiler_caecum_content	EUCAMP2	0,5	2	64	16	<=0,5	<=1	C. jejuni

Table S5: Minimum inhibitory concentrations of 6 antimicrobials for isolats of C. jejuni and C. coli from poultry

Year	Sample No.	Matrix_final	Antimicrobial panel	GEN	STR	NAL	CIP	TET	ERY	Species
2016	16-CA00810	Broiler_caecum_content	EUCAMP2	<=0,125	<=0,25	16	16	16	<=1	C. jejuni
2016	16-CA00811	Broiler_caecum_content	EUCAMP2	0,5	1	64	>16	>64	<=1	C. jejuni
2016	16-CA00812	Broiler_caecum_content	EUCAMP2	0,25	1	32	8	8	<=1	C. coli
2016	16-CA00813	Turkey_caecum_content	EUCAMP2	0,5	1	64	>16	2	2	C. coli
2016	16-CA00814	Turkey_caecum_content	EUCAMP2	0,25	0,5	32	16	64	<=1	C. jejuni
2016	16-CA00815	Turkey_caecum_content	EUCAMP2	0,5	2	4	0,25	<=0,5	<=1	C. jejuni
2016	16-CA00816	Turkey_caecum_content	EUCAMP2	0,5	2	64	>16	2	2	C. coli
2016	16-CA00817	Turkey_caecum_content	EUCAMP2	0,5	1	32	>16	>64	<=1	C. jejuni
2016	16-CA00818	Turkey_caecum_content	EUCAMP2	0,25	2	32	16	32	<=1	C. coli
2016	16-CA00819	Turkey_caecum_content	EUCAMP2	0,5	1	4	0,25	<=0,5	<=1	C. jejuni
2016	16-CA00820	Turkey_caecum_content	EUCAMP2	0,5	1	64	>16	<=0,5	<=1	C. jejuni
2016	16-CA00821	Turkey_caecum_content	EUCAMP2	0,25	1	32	8	16	<=1	C. coli
2016	16-CA00822	Turkey_caecum_content	EUCAMP2	<=0,125	0,5	16	>16	16	<=1	C. jejuni
2016	16-CA00823	Turkey_caecum_content	EUCAMP2	0,5	1	4	0,25	<=0,5	<=1	C. jejuni
2016	16-CA00824	Turkey_caecum_content	EUCAMP2	<=0,125	0,5	32	>16	<=0,5	<=1	C. jejuni
2016	16-CA00825	Turkey_caecum_content	EUCAMP2	<=0,125	0,5	32	>16	16	<=1	C. jejuni
2016	16-CA00826	Turkey_caecum_content	EUCAMP2	0,5	1	4	0,25	4	<=1	C. jejuni
2016	16-CA00827	Turkey_caecum_content	EUCAMP2	0,25	1	32	>16	>64	<=1	C. jejuni
2016	16-CA00828	Broiler_caecum_content	EUCAMP2	0,25	0,5	32	>16	64	<=1	C. jejuni
2016	16-CA00829	Broiler_caecum_content	EUCAMP2	0,5	1	64	>16	>64	2	C. jejuni
2016	16-CA00830	Turkey_caecum_content	EUCAMP2	0,25	2	>64	>16	>64	4	C. jejuni
2016	16-CA00831	Turkey_caecum_content	EUCAMP2	0,25	0,5	32	16	64	2	C. coli
2016	16-CA00832	Turkey_caecum_content	EUCAMP2	0,5	1	4	0,25	<=0,5	<=1	C. jejuni
2016	16-CA00833	Turkey_caecum_content	EUCAMP2	0,5	1	32	>16	32	4	C. coli
2016	16-CA00834	Turkey_caecum_content	EUCAMP2	0,5	2	64	>16	32	4	C. coli
2016	16-CA00835	Turkey_caecum_content	EUCAMP2	0,25	0,5	32	8	16	<=1	C. jejuni
2016	16-CA00836	Turkey_caecum_content	EUCAMP2	<=0,125	0,5	32	16	64	<=1	C. jejuni
2016	16-CA00837	Turkey_caecum_content	EUCAMP2	0,25	1	32	>16	8	<=1	C. jejuni
2016	16-CA00838	Turkey_caecum_content	EUCAMP2	0,25	0,5	32	>16	>64	<=1	C. jejuni
2016	16-CA00839	Turkey_caecum_content	EUCAMP2	0,5	1	32	>16	8	<=1	C. jejuni
2016	16-CA00840	Turkey_caecum_content	EUCAMP2	0,5	2	32	>16	32	4	C. coli
2016	16-CA00841	Broiler_caecum_content	EUCAMP2	0,25	0,5	32	16	16	<=1	C. jejuni

Table S5: Minimum inhibitory concentrations of 6 antimicrobials for isolats of C. jejuni and C. coli from poultry

Year	Sample No.	Matrix_final	Antimicrobial panel	GEN	STR	NAL	CIP	TET	ERY	Species
2016	16-CA00842	Broiler_caecum_content	EUCAMP2	0,25	0,5	32	16	32	<=1	C. jejuni
2016	16-CA00843	Broiler_caecum_content	EUCAMP2	0,5	1	64	16	64	<=1	C. jejuni
2016	16-CA00844	Turkey_caecum_content	EUCAMP2	0,5	2	8	0,5	<=0,5	2	C. jejuni
2016	16-CA00845	Turkey_caecum_content	EUCAMP2	<=0,125	1	32	16	32	<=1	C. jejuni
2016	16-CA00846	Turkey_caecum_content	EUCAMP2	0,25	0,5	4	<=0,125	16	<=1	C. jejuni
2016	16-CA00847	Turkey_caecum_content	EUCAMP2	0,5	1	4	0,25	<=0,5	<=1	C. jejuni
2016	16-CA00848	Turkey_caecum_content	EUCAMP2	0,5	2	32	16	<=0,5	<=1	C. jejuni
2016	16-CA00849	Turkey_caecum_content	EUCAMP2	0,5	2	64	>16	>64	2	C. jejuni
2016	16-CA00850	Turkey_caecum_content	EUCAMP2	1	8	32	>16	64	2	C. coli
2016	16-CA00851	Turkey_caecum_content	EUCAMP2	0,25	0,5	2	<=0,125	<=0,5	<=1	C. jejuni
2016	16-CA00852	Turkey_caecum_content	EUCAMP2	0,25	1	64	>16	>64	4	C. coli
2016	16-CA00853	Turkey_caecum_content	EUCAMP2	0,5	2	64	16	<=0,5	<=1	C. jejuni
2016	16-CA00854	Turkey_caecum_content	EUCAMP2	0,5	1	32	16	<=0,5	<=1	C. coli
2016	16-CA00855	Broiler_caecum_content	EUCAMP2	<=0,125	1	2	<=0,125	<=0,5	<=1	C. jejuni
2016	16-CA00856	Broiler_caecum_content	EUCAMP2	0,5	2	4	0,25	<=0,5	<=1	C. jejuni
2016	16-CA00857	Broiler_caecum_content	EUCAMP2	1	2	4	0,5	>64	<=1	C. jejuni
2016	16-CA00858	Turkey_caecum_content	EUCAMP2	<=0,125	<=0,25	4	2	16	64	C. coli
2016	16-CA00859	Turkey_caecum_content	EUCAMP2	1	2	8	0,25	<=0,5	<=1	C. jejuni
2016	16-CA00860	Turkey_caecum_content	EUCAMP2	0,5	1	64	>16	>64	<=1	C. jejuni
2016	16-CA00861	Turkey_caecum_content	EUCAMP2	0,5	1	4	<=0,125	<=0,5	<=1	C. jejuni
2016	16-CA00862	Turkey_caecum_content	EUCAMP2	0,25	2	64	16	64	<=1	C. coli
2016	16-CA00863	Turkey_caecum_content	EUCAMP2	0,25	1	>64	>16	1	<=1	C. jejuni
2016	16-CA00864	Turkey_caecum_content	EUCAMP2	0,5	>16	16	8	16	128	C. coli
2016	16-CA00865	Turkey_caecum_content	EUCAMP2	0,5	0,5	4	0,25	<=0,5	<=1	C. jejuni
2016	16-CA00866	Turkey_caecum_content	EUCAMP2	0,25	1	16	8	<=0,5	<=1	C. coli
2016	16-CA00867	Turkey_caecum_content	EUCAMP2	0,5	1	32	16	1	<=1	C. coli
2016	16-CA00868	Turkey_caecum_content	EUCAMP2	0,5	1	32	16	16	<=1	C. coli
2016	16-CA00869	Turkey_caecum_content	EUCAMP2	0,5	1	32	>16	>64	<=1	C. jejuni
2016	16-CA00870	Turkey_caecum_content	EUCAMP2	0,5	1	4	1	32	2	C. jejuni
2016	16-CA00871	Turkey_caecum_content	EUCAMP2	0,5	1	32	>16	>64	<=1	C. jejuni
2016	16-CA00872	Turkey_caecum_content	EUCAMP2	0,25	0,5	32	>16	<=0,5	<=1	C. jejuni
2016	16-CA00896	Broiler_caecum_content	EUCAMP2	0,5	1	64	8	<=0,5	<=1	C. jejuni

Table S5: Minimum inhibitory concentrations of 6 antimicrobials for isolats of C. jejuni and C. coli from poultry

Year	Sample No.	Matrix_final	Antimicrobial panel	GEN	STR	NAL	CIP	TET	ERY	Species
2016	16-CA00897	Broiler_caecum_content	EUCAMP2	0,25	0,5	32	16	64	<=1	C. jejuni
2016	16-CA00901	Broiler_caecum_content	EUCAMP2	0,5	2	64	16	<=0,5	<=1	C. jejuni
2016	16-CA00923	Broiler_caecum_content	EUCAMP2	0,5	1	32	16	64	<=1	C. jejuni
2016	16-CA00952	Broiler_caecum_content	EUCAMP2	0,5	2	32	>16	32	2	C. coli
2016	16-CA00953	Broiler_caecum_content	EUCAMP2	1	4	64	16	4	<=1	C. coli
2016	16-CA00954	Broiler_caecum_content	EUCAMP2	0,5	2	4	0,25	<=0,5	<=1	C. jejuni
2016	16-CA00965	Broiler_caecum_content	EUCAMP2	0,5	2	64	>16	>64	<=1	C. jejuni
2016	16-CA00967	Broiler_caecum_content	EUCAMP2	0,25	1	64	16	>64	<=1	C. jejuni
2016	16-CA00975	Broiler_caecum_content	EUCAMP2	0,5	1	32	16	>64	<=1	C. jejuni
2016	16-CA00976	Broiler_caecum_content	EUCAMP2	0,25	1	32	16	32	<=1	C. jejuni
2016	16-CA00986	Broiler_caecum_content	EUCAMP2	0,5	1	4	<=0,125	<=0,5	<=1	C. jejuni
2016	16-CA00987	Broiler_caecum_content	EUCAMP2	0,5	2	64	16	<=0,5	<=1	C. jejuni
2016	16-CA00991	Turkey_caecum_content	EUCAMP2	0,25	1	32	16	32	<=1	C. jejuni
2016	16-CA01008	Broiler_caecum_content	EUCAMP2	1	1	>64	>16	<=0,5	<=1	C. jejuni
2016	16-CA01010	Broiler_caecum_content	EUCAMP2	0,5	1	4	<=0,125	1	<=1	C. jejuni
2016	16-CA01011	Turkey_caecum_content	EUCAMP2	0,25	1	32	16	64	<=1	C. jejuni
2016	16-CA01012	Broiler_caecum_content	EUCAMP2	8	16	32	8	32	<=1	C. jejuni
2016	16-CA01013	Broiler_caecum_content	EUCAMP2	0,5	2	32	16	64	<=1	C. jejuni
2016	16-CA01014	Broiler_caecum_content	EUCAMP2	1	2	2	<=0,125	<=0,5	<=1	C. jejuni
2016	16-CA01021	Turkey_caecum_content	EUCAMP2	0,5	1	4	0,25	<=0,5	<=1	C. jejuni
2016	16-CA01022	Turkey_caecum_content	EUCAMP2	0,25	1	32	16	64	<=1	C. jejuni
2016	16-CA01023	Turkey_caecum_content	EUCAMP2	1	2	4	0,25	<=0,5	<=1	C. jejuni
2016	16-CA01024	Turkey_caecum_content	EUCAMP2	0,5	1	4	0,25	<=0,5	<=1	C. coli
2016	16-CA01025	Turkey_caecum_content	EUCAMP2	0,5	>16	32	16	64	>128	C. coli
2016	16-CA01026	Broiler_caecum_content	EUCAMP2	1	2	4	0,5	<=0,5	<=1	C. jejuni
2016	16-CA01027	Broiler_caecum_content	EUCAMP2	0,5	1	64	8	<=0,5	<=1	C. jejuni
2016	16-CA01028	Turkey_caecum_content	EUCAMP2	0,5	1	8	0,5	>64	<=1	C. jejuni
2016	16-CA01029	Turkey_caecum_content	EUCAMP2	0,5	1	64	>16	>64	<=1	C. jejuni
2016	16-CA01030	Turkey_caecum_content	EUCAMP2	0,5	2	64	>16	<=0,5	<=1	C. coli
2016	16-CA01031	Turkey_caecum_content	EUCAMP2	0,5	1	32	16	<=0,5	<=1	C. jejuni
2016	16-CA01032	Turkey_caecum_content	EUCAMP2	0,25	1	64	>16	>64	4	C. coli
2016	16-CA01033	Turkey_caecum_content	EUCAMP2	0,25	0,5	32	>16	64	<=1	C. jejuni

Table S5: Minimum inhibitory concentrations of 6 antimicrobials for isolats of C. jejuni and C. coli from poultry

Year	Sample No.	Matrix_final	Antimicrobial panel	GEN	STR	NAL	CIP	TET	ERY	Species
2016	16-CA01034	Turkey_caecum_content	EUCAMP2	0,5	1	4	0,25	<=0,5	<=1	C. jejuni
2016	16-CA01035	Turkey_caecum_content	EUCAMP2	1	1	64	16	>64	<=1	C. jejuni
2016	16-CA01036	Turkey_caecum_content	EUCAMP2	0,5	1	64	16	32	2	C. jejuni
2016	16-CA01037	Turkey_caecum_content	EUCAMP2	0,5	1	32	>16	>64	<=1	C. jejuni
2016	16-CA01038	Turkey_caecum_content	EUCAMP2	0,5	1	>64	>16	<=0,5	2	C. jejuni
2016	16-CA01039	Turkey_caecum_content	EUCAMP2	1	2	>64	16	>64	<=1	C. jejuni
2016	16-CA01040	Turkey_caecum_content	EUCAMP2	1	2	4	0,25	<=0,5	<=1	C. jejuni
2016	16-CA01041	Turkey_caecum_content	EUCAMP2	0,5	1	4	0,5	>64	<=1	C. jejuni
2016	16-CA01042	Turkey_caecum_content	EUCAMP2	0,5	1	>64	>16	>64	2	C. jejuni
2016	16-CA01043	Broiler_caecum_content	EUCAMP2	0,25	0,5	64	16	<=0,5	<=1	C. jejuni
2016	16-CA01044	Broiler_caecum_content	EUCAMP2	0,5	1	4	0,25	<=0,5	<=1	C. jejuni
2016	16-CA01045	Broiler_caecum_content	EUCAMP2	0,5	1	4	0,25	<=0,5	<=1	C. jejuni
2016	16-CA01046	Broiler_caecum_content	EUCAMP2	<=0,125	<=0,25	32	16	<=0,5	<=1	C. jejuni
2016	16-CA01060	Broiler_caecum_content	EUCAMP2	0,25	1	64	>16	64	2	C. coli
2016	16-CA01061	Broiler_caecum_content	EUCAMP2	0,5	1	4	0,25	<=0,5	<=1	C. jejuni
2016	16-CA01083	Broiler_caecum_content	EUCAMP2	1	2	4	0,25	<=0,5	<=1	C. jejuni
2016	16-CA01107	Broiler_caecum_content	EUCAMP2	0,5	1	64	16	64	<=1	C. jejuni
2016	16-CA01124	Broiler_caecum_content	EUCAMP2	0,5	1	64	16	64	<=1	C. jejuni
2016	16-CA01125	Broiler_caecum_content	EUCAMP2	0,25	0,5	32	16	16	<=1	C. jejuni
2016	16-CA01126	Broiler_caecum_content	EUCAMP2	0,25	2	32	16	32	<=1	C. coli
2016	16-CA01127	Broiler_caecum_content	EUCAMP2	0,5	1	32	16	32	<=1	C. coli
2016	16-CA01128	Turkey_caecum_content	EUCAMP2	0,25	1	32	16	16	<=1	C. coli
2016	16-CA01129	Turkey_caecum_content	EUCAMP2	0,5	1	<=1	16	<=0,5	<=1	C. jejuni
2016	16-CA01130	Turkey_caecum_content	EUCAMP2	0,25	0,5	32	16	32	<=1	C. jejuni
2016	16-CA01131	Turkey_caecum_content	EUCAMP2	0,25	1	32	16	32	2	C. coli
2016	16-CA01132	Turkey_caecum_content	EUCAMP2	0,5	2	64	16	64	<=1	C. jejuni
2016	16-CA01133	Turkey_caecum_content	EUCAMP2	0,25	4	32	4	8	<=1	C. coli
2016	16-CA01134	Turkey_caecum_content	EUCAMP2	0,25	1	32	>16	<=0,5	<=1	C. jejuni
2016	16-CA01135	Broiler_caecum_content	EUCAMP2	0,5	2	4	0,25	<=0,5	<=1	C. jejuni
2016	16-CA01136	Broiler_caecum_content	EUCAMP2	0,5	2	64	>16	>64	<=1	C. jejuni
2016	16-CA01137	Broiler_caecum_content	EUCAMP2	0,5	1	64	>16	64	<=1	C. jejuni
2016	16-CA01138	Broiler_caecum_content	EUCAMP2	0,25	1	16	8	16	<=1	C. coli

Table S5: Minimum inhibitory concentrations of 6 antimicrobials for isolats of C. jejuni and C. coli from poultry

Year	Sample No.	Matrix_final	Antimicrobial panel	GEN	STR	NAL	CIP	TET	ERY	Species
2016	16-CA01139	Turkey_caecum_content	EUCAMP2	0,5	1	64	>16	64	<=1	C. jejuni
2016	16-CA01140	Turkey_caecum_content	EUCAMP2	0,25	2	32	16	16	<=1	C. coli
2016	16-CA01141	Turkey_caecum_content	EUCAMP2	0,25	0,5	32	16	64	<=1	C. jejuni
2016	16-CA01142	Turkey_caecum_content	EUCAMP2	0,25	1	32	16	16	<=1	C. coli
2016	16-CA01143	Turkey_caecum_content	EUCAMP2	0,5	2	64	>16	>64	<=1	C. jejuni
2016	16-CA01144	Turkey_caecum_content	EUCAMP2	0,25	1	32	>16	>64	<=1	C. jejuni
2016	16-CA01145	Turkey_caecum_content	EUCAMP2	0,5	1	64	>16	>64	2	C. jejuni
2016	16-CA01146	Turkey_caecum_content	EUCAMP2	0,5	1	64	>16	>64	2	C. jejuni
2016	16-CA01147	Broiler_caecum_content	EUCAMP2	0,25	1	2	<=0,125	<=0,5	<=1	C. jejuni
2016	16-CA01148	Broiler_caecum_content	EUCAMP2	<=0,125	0,5	16	8	32	<=1	C. jejuni
2016	16-CA01149	Broiler_caecum_content	EUCAMP2	0,25	1	4	<=0,125	<=0,5	<=1	C. jejuni
2016	16-CA01150	Turkey_caecum_content	EUCAMP2	0,5	1	64	>16	>64	2	C. jejuni
2016	16-CA01151	Turkey_caecum_content	EUCAMP2	0,5	1	32	>16	>64	2	C. jejuni
2016	16-CA01152	Turkey_caecum_content	EUCAMP2	<=0,125	<=0,25	32	16	32	<=1	C. jejuni
2016	16-CA01153	Turkey_caecum_content	EUCAMP2	0,5	2	64	>16	>64	<=1	C. jejuni
2016	16-CA01154	Turkey_caecum_content	EUCAMP2	0,5	8	32	16	64	>128	C. coli
2016	16-CA01155	Turkey_caecum_content	EUCAMP2	0,25	0,5	64	16	64	<=1	C. jejuni
2016	16-CA01156	Turkey_caecum_content	EUCAMP2	0,5	2	64	>16	>64	<=1	C. coli
2016	16-CA01157	Turkey_caecum_content	EUCAMP2	0,5	2	64	16	64	<=1	C. coli
2016	16-CA01158	Turkey_caecum_content	EUCAMP2	0,5	1	32	8	<=0,5	<=1	C. jejuni
2016	16-CA01159	Turkey_caecum_content	EUCAMP2	0,25	1	32	16	64	2	C. coli
2016	16-CA01160	Turkey_caecum_content	EUCAMP2	0,5	1	32	>16	16	2	C. coli
2016	16-CA01161	Broiler_caecum_content	EUCAMP2	0,25	1	32	16	32	<=1	C. coli
2016	16-CA01162	Broiler_caecum_content	EUCAMP2	1	2	64	16	64	<=1	C. coli
2016	16-CA01163	Turkey_caecum_content	EUCAMP2	0,5	2	32	16	64	>128	C. coli
2016	16-CA01164	Turkey_caecum_content	EUCAMP2	0,5	1	4	0,25	64	<=1	C. jejuni
2016	16-CA01165	Turkey_caecum_content	EUCAMP2	0,5	1	64	>16	>64	<=1	C. jejuni
2016	16-CA01166	Turkey_caecum_content	EUCAMP2	<=0,125	<=0,25	32	16	8	<=1	C. jejuni
2016	16-CA01167	Turkey_caecum_content	EUCAMP2	<=0,125	2	32	16	64	2	C. coli
2016	16-CA01168	Turkey_caecum_content	EUCAMP2	1	2	64	16	<=0,5	<=1	C. jejuni
2016	16-CA01169	Turkey_caecum_content	EUCAMP2	0,5	1	32	16	32	<=1	C. jejuni
2016	16-CA01170	Turkey_caecum_content	EUCAMP2	0,25	1	4	0,25	<=0,5	<=1	C. jejuni

Table S5: Minimum inhibitory concentrations of 6 antimicrobials for isolats of C. jejuni and C. coli from poultry

Year	Sample No.	Matrix_final	Antimicrobial panel	GEN	STR	NAL	CIP	TET	ERY	Species
2016	16-CA01171	Turkey_caecum_content	EUCAMP2	0,5	1	32	16	32	<=1	C. coli
2016	16-CA01172	Turkey_caecum_content	EUCAMP2	1	8	8	0,5	<=0,5	4	C. coli
2016	16-CA01173	Broiler_caecum_content	EUCAMP2	0,5	1	32	16	<=0,5	<=1	C. jejuni
2016	16-CA01174	Broiler_caecum_content	EUCAMP2	0,5	1	64	>16	>64	2	C. jejuni
2016	16-CA01175	Broiler_caecum_content	EUCAMP2	0,5	2	32	8	16	<=1	C. coli
2016	16-CA01176	Broiler_caecum_content	EUCAMP2	0,5	2	2	<=0,125	8	<=1	C. coli
2016	16-CA01177	Broiler_caecum_content	EUCAMP2	0,5	1	64	>16	64	<=1	C. jejuni
2016	16-CA01178	Broiler_caecum_content	EUCAMP2	0,25	1	64	16	>64	<=1	C. jejuni
2016	16-CA01179	Broiler_caecum_content	EUCAMP2	0,25	0,5	32	8	<=0,5	<=1	C. jejuni
2016	16-CA01180	Broiler_caecum_content	EUCAMP2	0,25	1	32	16	32	2	C. coli
2016	16-CA01181	Turkey_caecum_content	EUCAMP2	0,5	2	32	16	16	<=1	C. coli
2016	16-CA01182	Turkey_caecum_content	EUCAMP2	0,5	1	32	16	16	>128	C. coli
2016	16-CA01183	Turkey_caecum_content	EUCAMP2	0,25	1	64	>16	>64	2	C. jejuni
2016	16-CA01184	Turkey_caecum_content	EUCAMP2	0,25	0,5	8	4	4	<=1	C. jejuni
2016	16-CA01185	Turkey_caecum_content	EUCAMP2	0,25	1	32	16	32	2	C. coli
2016	16-CA01186	Turkey_caecum_content	EUCAMP2	0,25	1	4	0,25	<=0,5	<=1	C. coli
2016	16-CA01187	Turkey_caecum_content	EUCAMP2	0,25	1	32	16	16	<=1	C. coli
2016	16-CA01188	Turkey_caecum_content	EUCAMP2	0,5	>16	64	8	32	<=1	C. coli
2016	16-CA01189	Turkey_caecum_content	EUCAMP2	0,25	0,5	32	16	32	<=1	C. jejuni
2016	16-CA01190	Turkey_caecum_content	EUCAMP2	0,5	1	32	16	64	<=1	C. jejuni
2016	16-CA01191	Turkey_caecum_content	EUCAMP2	0,25	1	32	4	8	<=1	C. coli
2016	16-CA01192	Turkey_caecum_content	EUCAMP2	0,5	2	4	0,25	<=0,5	<=1	C. jejuni
2016	16-CA01207	Broiler_caecum_content	EUCAMP2	<=0,125	>16	2	>16	64	<=1	C. jejuni
2016	16-CA01208	Broiler_caecum_content	EUCAMP2	0,5	2	64	16	64	<=1	C. coli
2016	16-CA01208	Broiler_caecum_content	EUCAMP2	0,25	0,5	32	16	32	<=1	C. jejuni
2016	16-CA01209	Broiler_caecum_content	EUCAMP2	0,5	2	64	>16	>64	<=1	C. jejuni
2016	16-CA01210	Broiler_caecum_content	EUCAMP2	0,5	1	2	<=0,125	<=0,5	<=1	C. jejuni
2016	16-CA01211	Broiler_caecum_content	EUCAMP2	0,5	1	32	16	32	<=1	C. jejuni
2016	16-CA01216	Broiler_caecum_content	EUCAMP2	0,5	2	4	<=0,125	<=0,5	<=1	C. jejuni
2016	16-CA01220	Turkey_caecum_content	EUCAMP2	0,5	1	32	>16	64	2	C. coli
2016	16-CA01221	Turkey_caecum_content	EUCAMP2	0,25	1	64	>16	<=0,5	<=1	C. jejuni
2016	16-CA01222	Turkey_caecum_content	EUCAMP2	0,5	2	32	16	<=0,5	<=1	C. jejuni

Table S5: Minimum inhibitory concentrations of 6 antimicrobials for isolats of C. jejuni and C. coli from poultry

Year	Sample No.	Matrix_final	Antimicrobial panel	GEN	STR	NAL	CIP	TET	ERY	Species
2016	16-CA01223	Turkey_caecum_content	EUCAMP2	1	8	64	>16	64	2	C. coli
2016	16-CA01240	Turkey_caecum_content	EUCAMP2	0,5	1	32	>16	>64	<=1	C. jejuni
2016	16-CA01241	Turkey_caecum_content	EUCAMP2	0,25	>16	32	16	<=0,5	<=1	C. jejuni
2016	16-CA01268	Broiler_caecum_content	EUCAMP2	0,5	>16	16	8	4	<=1	C. coli
2016	16-CA01269	Broiler_caecum_content	EUCAMP2	0,25	1	64	8	<=0,5	<=1	C. jejuni
2016	16-CA01270	Broiler_caecum_content	EUCAMP2	0,5	1	4	<=0,125	<=0,5	<=1	C. jejuni
2016	16-CA01276	Broiler_caecum_content	EUCAMP2	0,5	1	64	8	<=0,5	<=1	C. jejuni
2016	16-CA01277	Broiler_caecum_content	EUCAMP2	0,5	2	64	16	32	<=1	C. jejuni
2016	16-CA01279	Broiler_caecum_content	EUCAMP2	0,5	1	64	16	<=0,5	<=1	C. jejuni
2016	16-CA01280	Broiler_caecum_content	EUCAMP2	0,5	2	64	>16	>64	2	C. jejuni
2016	16-CA01281	Broiler_caecum_content	EUCAMP2	1	8	>64	16	<=0,5	<=1	C. jejuni
2016	16-CA01282	Turkey_caecum_content	EUCAMP2	0,5	1	32	4	<=0,5	<=1	C. coli
2016	16-CA01292	Broiler_caecum_content	EUCAMP2	0,5	>16	64	8	<=0,5	<=1	C. jejuni
2016	16-CA01293	Broiler_caecum_content	EUCAMP2	0,5	1	4	<=0,125	<=0,5	<=1	C. jejuni
2016	16-CA01294	Turkey_caecum_content	EUCAMP2	0,5	2	16	8	64	<=1	C. coli
2016	16-CA01295	Broiler_caecum_content	EUCAMP2	0,5	>16	64	16	>64	<=1	C. jejuni
2016	16-CA01297	Turkey_caecum_content	EUCAMP2	0,5	1	32	16	32	<=1	C. jejuni
2016	16-CA01298	Turkey_caecum_content	EUCAMP2	0,5	>16	32	8	8	<=1	C. coli
2016	16-CA01299	Turkey_caecum_content	EUCAMP2	0,5	1	64	>16	<=0,5	<=1	C. jejuni
2016	16-CA01300	Turkey_caecum_content	EUCAMP2	1	2	4	0,25	<=0,5	<=1	C. jejuni
2016	16-CA01301	Turkey_caecum_content	EUCAMP2	0,25	1	32	8	16	2	C. coli
2016	16-CA01302	Turkey_caecum_content	EUCAMP2	0,5	1	32	16	64	<=1	C. jejuni
2016	16-CA01303	Turkey_caecum_content	EUCAMP2	0,5	1	32	16	32	<=1	C. jejuni
2016	16-CA01304	Turkey_caecum_content	EUCAMP2	0,5	1	32	16	32	<=1	C. jejuni
2016	16-CA01305	Turkey_caecum_content	EUCAMP2	0,25	0,5	32	16	32	<=1	C. jejuni
2016	16-CA01306	Broiler_caecum_content	EUCAMP2	0,5	1	64	>16	>64	2	C. jejuni
2016	16-CA01307	Broiler_caecum_content	EUCAMP2	0,25	1	64	>16	>64	<=1	C. jejuni
2016	16-CA01308	Broiler_caecum_content	EUCAMP2	0,25	1	16	16	8	>128	C. coli
2016	16-CA01309	Turkey_caecum_content	EUCAMP2	0,5	1	32	8	<=0,5	<=1	C. coli
2016	16-CA01310	Turkey_caecum_content	EUCAMP2	1	2	4	0,25	<=0,5	<=1	C. jejuni
2016	16-CA01311	Turkey_caecum_content	EUCAMP2	0,25	1	32	16	32	<=1	C. coli
2016	16-CA01312	Turkey_caecum_content	EUCAMP2	0,5	1	4	<=0,125	<=0,5	<=1	C. jejuni

Table S5: Minimum inhibitory concentrations of 6 antimicrobials for isolats of C. jejuni and C. coli from poultry

Year	Sample No.	Matrix_final	Antimicrobial panel	GEN	STR	NAL	CIP	TET	ERY	Species
2016	16-CA01313	Turkey_caecum_content	EUCAMP2	0,5	2	4	0,25	<=0,5	<=1	C. jejuni
2016	16-CA01314	Broiler_caecum_content	EUCAMP2	0,5	1	32	16	32	2	C. jejuni
2016	16-CA01315	Broiler_caecum_content	EUCAMP2	0,5	2	32	16	64	2	C. coli
2016	16-CA01316	Broiler_caecum_content	EUCAMP2	0,5	1	32	16	32	<=1	C. jejuni
2016	16-CA01317	Turkey_caecum_content	EUCAMP2	0,5	>16	16	4	8	<=1	C. coli
2016	16-CA01318	Turkey_caecum_content	EUCAMP2	0,5	1	16	8	<=0,5	>128	C. coli
2016	16-CA01319	Turkey_caecum_content	EUCAMP2	0,25	>16	32	16	64	>128	C. coli
2016	16-CA01320	Turkey_caecum_content	EUCAMP2	0,5	2	16	8	<=0,5	<=1	C. coli
2016	16-CA01321	Turkey_caecum_content	EUCAMP2	0,25	1	64	>16	>64	<=1	C. jejuni
2016	16-CA01322	Turkey_caecum_content	EUCAMP2	0,5	1	64	16	>64	<=1	C. jejuni
2016	16-CA01323	Turkey_caecum_content	EUCAMP2	0,5	2	32	16	64	<=1	C. coli
2016	16-CA01324	Turkey_caecum_content	EUCAMP2	0,5	2	64	16	<=0,5	<=1	C. jejuni
2016	16-CA01325	Broiler_caecum_content	EUCAMP2	0,5	1	32	16	64	<=1	C. jejuni
2016	16-CA01326	Broiler_caecum_content	EUCAMP2	0,5	1	64	16	64	<=1	C. jejuni
2016	16-CA01327	Broiler_caecum_content	EUCAMP2	<=0,125	0,5	32	>16	64	<=1	C. jejuni
2016	16-CA01328	Broiler_caecum_content	EUCAMP2	0,5	1	64	>16	64	2	C. jejuni
2016	16-CA01329	Turkey_caecum_content	EUCAMP2	0,25	1	32	>16	>64	<=1	C. jejuni
2016	16-CA01330	Turkey_caecum_content	EUCAMP2	0,5	1	>64	>16	>64	2	C. jejuni
2016	16-CA01331	Turkey_caecum_content	EUCAMP2	0,5	1	32	16	>64	>128	C. coli
2016	16-CA01332	Turkey_caecum_content	EUCAMP2	0,5	1	64	16	<=0,5	<=1	C. jejuni
2016	16-CA01333	Turkey_caecum_content	EUCAMP2	0,5	1	64	>16	<=0,5	<=1	C. jejuni
2016	16-CA01334	Turkey_caecum_content	EUCAMP2	0,5	1	64	16	<=0,5	2	C. coli
2016	16-CA01335	Turkey_caecum_content	EUCAMP2	<=0,125	0,5	64	>16	>64	<=1	C. jejuni
2016	16-CA01336	Broiler_caecum_content	EUCAMP2	0,25	1	64	>16	>64	<=1	C. jejuni
2016	16-CA01337	Broiler_caecum_content	EUCAMP2	0,5	2	>64	>16	>64	2	C. jejuni
2016	16-CA01338	Broiler_caecum_content	EUCAMP2	0,5	1	64	>16	>64	<=1	C. jejuni
2016	16-CA01339	Broiler_caecum_content	EUCAMP2	0,5	1	64	>16	>64	2	C. jejuni
2016	16-CA01340	Broiler_caecum_content	EUCAMP2	0,5	1	32	16	32	<=1	C. jejuni
2016	16-CA01341	Broiler_caecum_content	EUCAMP2	0,5	1	64	>16	>64	2	C. jejuni
2016	16-CA01342	Turkey_caecum_content	EUCAMP2	0,5	1	32	8	<=0,5	<=1	C. coli
2016	16-CA01343	Turkey_caecum_content	EUCAMP2	0,5	1	32	16	32	<=1	C. jejuni
2016	16-CA01344	Turkey_caecum_content	EUCAMP2	0,5	1	64	>16	>64	<=1	C. jejuni

Table S5: Minimum inhibitory concentrations of 6 antimicrobials for isolats of C. jejuni and C. coli from poultry

Year	Sample No.	Matrix_final	Antimicrobial panel	GEN	STR	NAL	CIP	TET	ERY	Species
2016	16-CA01345	Turkey_caecum_content	EUCAMP2	0,25	1	4	0,25	<=0,5	<=1	C. jejuni
2016	16-CA01346	Turkey_caecum_content	EUCAMP2	0,5	2	32	16	32	<=1	C. coli
2016	16-CA01347	Turkey_caecum_content	EUCAMP2	0,5	1	64	>16	>64	<=1	C. jejuni
2016	16-CA01348	Turkey_caecum_content	EUCAMP2	0,5	2	64	>16	>64	4	C. coli
2016	16-CA01349	Turkey_caecum_content	EUCAMP2	0,5	2	>64	16	4	<=1	C. coli
2016	16-CA01350	Broiler_caecum_content	EUCAMP2	0,5	1	64	>16	>64	<=1	C. jejuni
2016	16-CA01351	Broiler_caecum_content	EUCAMP2	0,5	1	32	16	32	<=1	C. jejuni
2016	16-CA01352	Broiler_caecum_content	EUCAMP2	0,25	0,5	32	16	16	<=1	C. jejuni
2016	16-CA01353	Broiler_caecum_content	EUCAMP2	0,5	2	4	0,25	<=0,5	<=1	C. jejuni
2016	16-CA01468	Broiler_caecum_content	EUCAMP2	0,5	2	64	16	64	<=1	C. coli
2016	16-CA01469	Broiler_caecum_content	EUCAMP2	0,5	2	64	16	64	<=1	C. coli
2016	16-CA01484	Broiler_caecum_content	EUCAMP2	0,5	1	32	16	64	<=1	C. jejuni
2016	16-CA01517	Broiler_caecum_content	EUCAMP2	0,25	1	32	16	64	<=1	C. jejuni
2016	16-CA01518	Broiler_caecum_content	EUCAMP2	0,25	1	64	16	64	<=1	C. jejuni
2016	16-CA01528	Turkey_caecum_content	EUCAMP2	0,25	1	32	16	<=0,5	<=1	C. jejuni
2016	16-CA01529	Turkey_caecum_content	EUCAMP2	0,25	1	64	8	<=0,5	<=1	C. coli
2016	16-CA01530	Turkey_caecum_content	EUCAMP2	0,25	1	64	>16	<=0,5	2	C. jejuni
2016	16-CA01531	Turkey_caecum_content	EUCAMP2	0,5	1	4	0,25	<=0,5	<=1	C. jejuni
2016	16-CA01532	Turkey_caecum_content	EUCAMP2	0,5	1	32	16	64	2	C. coli
2016	16-CA01533	Turkey_caecum_content	EUCAMP2	0,5	1	64	16	>64	2	C. coli
2016	16-CA01534	Turkey_caecum_content	EUCAMP2	0,5	1	32	8	32	<=1	C. coli
2016	16-CA01535	Turkey_caecum_content	EUCAMP2	0,5	2	32	8	<=0,5	<=1	C. coli
2016	16-CA01536	Turkey_caecum_content	EUCAMP2	0,25	1	32	8	32	<=1	C. coli
2016	16-CA01537	Broiler_caecum_content	EUCAMP2	0,5	1	32	8	<=0,5	<=1	C. coli
2016	16-CA01538	Turkey_caecum_content	EUCAMP2	0,25	0,5	2	<=0,125	16	<=1	C. coli
2016	16-CA01539	Turkey_caecum_content	EUCAMP2	0,5	1	64	16	64	<=1	C. jejuni
2016	16-CA01540	Turkey_caecum_content	EUCAMP2	0,25	0,5	32	16	16	<=1	C. coli
2016	16-CA01541	Turkey_caecum_content	EUCAMP2	0,5	2	64	16	32	<=1	C. coli
2016	16-CA01542	Turkey_caecum_content	EUCAMP2	0,5	0,5	32	16	32	<=1	C. jejuni
2016	16-CA01543	Turkey_caecum_content	EUCAMP2	0,5	1	64	>16	<=0,5	2	C. coli
2016	16-CA01544	Turkey_caecum_content	EUCAMP2	0,5	1	64	>16	32	4	C. coli
2016	16-CA01545	Broiler_caecum_content	EUCAMP2	0,5	1	64	>16	>64	<=1	C. jejuni

Table S5: Minimum inhibitory concentrations of 6 antimicrobials for isolats of C. jejuni and C. coli from poultry

Year	Sample No.	Matrix_final	Antimicrobial panel	GEN	STR	NAL	CIP	TET	ERY	Species
2016	16-CA01546	Broiler_caecum_content	EUCAMP2	0,25	1	32	16	16	<=1	C. jejuni
2016	16-CA01547	Broiler_caecum_content	EUCAMP2	0,5	1	8	0,25	<=0,5	<=1	C. jejuni
2016	16-CA01548	Turkey_caecum_content	EUCAMP2	0,25	1	4	0,25	2	<=1	C. coli
2016	16-CA01549	Turkey_caecum_content	EUCAMP2	0,25	1	32	16	64	<=1	C. coli
2016	16-CA01550	Turkey_caecum_content	EUCAMP2	0,5	1	64	16	32	2	C. jejuni
2016	16-CA01551	Broiler_caecum_content	EUCAMP2	1	16	64	16	32	<=1	C. coli
2016	16-CA01552	Broiler_caecum_content	EUCAMP2	0,5	>16	64	>16	>64	>128	C. coli
2016	16-CA01553	Broiler_caecum_content	EUCAMP2	1	2	64	16	64	<=1	C. coli
2016	16-CA01553	Broiler_caecum_content	EUCAMP2	0,5	1	64	16	64	<=1	C. jejuni
2016	16-CA01554	Turkey_caecum_content	EUCAMP2	0,5	2	32	16	64	2	C. coli
2016	16-CA01555	Turkey_caecum_content	EUCAMP2	0,5	2	64	16	<=0,5	4	C. coli
2016	16-CA01556	Turkey_caecum_content	EUCAMP2	0,25	1	64	>16	>64	<=1	C. jejuni
2016	16-CA01557	Turkey_caecum_content	EUCAMP2	0,5	1	4	<=0,125	<=0,5	<=1	C. jejuni
2016	16-CA01558	Turkey_caecum_content	EUCAMP2	0,5	1	64	>16	64	2	C. coli
2016	16-CA01558	Turkey_caecum_content	EUCAMP2	0,25	1	64	>16	>64	<=1	C. jejuni
2016	16-CA01559	Turkey_caecum_content	EUCAMP2	<=0,125	<=0,25	2	<=0,125	<=0,5	<=1	C. jejuni
2016	16-CA01560	Turkey_caecum_content	EUCAMP2	<=0,125	0,5	32	8	32	<=1	C. coli
2016	16-CA01561	Broiler_caecum_content	EUCAMP2	0,5	1	32	16	32	<=1	C. jejuni
2016	16-CA01562	Broiler_caecum_content	EUCAMP2	0,5	1	2	16	<=0,5	<=1	C. jejuni
2016	16-CA01576	Broiler_caecum_content	EUCAMP2	0,25	1	32	8	<=0,5	<=1	C. coli
2016	16-CA01577	Broiler_caecum_content	EUCAMP2	0,25	0,5	64	8	<=0,5	<=1	C. coli
2016	16-CA01578	Broiler_caecum_content	EUCAMP2	0,5	1	4	<=0,125	<=0,5	<=1	C. jejuni
2016	16-CA01579	Broiler_caecum_content	EUCAMP2	0,25	0,5	4	<=0,125	<=0,5	<=1	C. jejuni
2016	16-CA01588	Broiler_caecum_content	EUCAMP2	0,5	1	32	8	16	2	C. coli
2016	16-CA01597	Turkey_caecum_content	EUCAMP2	0,5	1	4	0,25	<=0,5	<=1	C. jejuni
2016	16-CA01598	Broiler_caecum_content	EUCAMP2	<=0,125	1	32	16	64	<=1	C. jejuni
2016	16-CA01599	Broiler_caecum_content	EUCAMP2	0,25	0,5	32	>16	>64	<=1	C. jejuni
2016	16-CA01609	Turkey_caecum_content	EUCAMP2	<=0,125	0,5	32	8	<=0,5	<=1	C. coli
2016	16-CA01647	Broiler_caecum_content	EUCAMP2	0,25	1	64	>16	>64	<=1	C. jejuni
2016	16-CA01663	Turkey_caecum_content	EUCAMP2	1	2	64	16	<=0,5	<=1	C. jejuni
2016	16-CA01664	Turkey_caecum_content	EUCAMP2	1	>16	64	16	64	2	C. coli
2016	16-CA01665	Turkey_caecum_content	EUCAMP2	0,5	2	64	16	64	2	C. coli

Table S5: Minimum inhibitory concentrations of 6 antimicrobials for isolats of C. jejuni and C. coli from poultry

Year	Sample No.	Matrix_final	Antimicrobial panel	GEN	STR	NAL	CIP	TET	ERY	Species
2016	16-CA01666	Turkey_caecum_content	EUCAMP2	0,5	2	64	16	<=0,5	<=1	C. jejuni
2016	16-CA01678	Broiler_caecum_content	EUCAMP2	0,25	1	<=1	>16	<=0,5	<=1	C. jejuni
2016	16-CA01679	Turkey_caecum_content	EUCAMP2	0,5	2	64	16	32	<=1	C. jejuni
2016	16-CA01680	Turkey_caecum_content	EUCAMP2	0,5	1	32	16	32	<=1	C. jejuni
2016	16-CA01681	Turkey_caecum_content	EUCAMP2	0,5	1	64	>16	>64	2	C. jejuni
2016	16-CA01682	Turkey_caecum_content	EUCAMP2	0,5	>16	32	>16	64	>128	C. coli
2016	16-CA01683	Turkey_caecum_content	EUCAMP2	0,5	2	4	0,25	<=0,5	<=1	C. jejuni
2016	16-CA01684	Turkey_caecum_content	EUCAMP2	1	>16	>64	>16	>64	4	C. coli
2016	16-CA01685	Turkey_caecum_content	EUCAMP2	0,5	>16	32	16	64	>128	C. coli
2016	16-CA01686	Turkey_caecum_content	EUCAMP2	0,25	1	>64	>16	<=0,5	<=1	C. jejuni
2016	16-CA01687	Broiler_caecum_content	EUCAMP2	0,5	1	4	<=0,125	<=0,5	<=1	C. jejuni
2016	16-CA01688	Broiler_caecum_content	EUCAMP2	0,5	1	64	8	<=0,5	<=1	C. jejuni
2016	16-CA01689	Broiler_caecum_content	EUCAMP2	0,25	0,5	32	>16	>64	<=1	C. jejuni
2016	16-CA01690	Turkey_caecum_content	EUCAMP2	0,5	1	32	16	64	2	C. coli
2016	16-CA01691	Turkey_caecum_content	EUCAMP2	0,5	>16	32	16	64	2	C. coli
2016	16-CA01692	Turkey_caecum_content	EUCAMP2	0,5	1	4	<=0,125	<=0,5	<=1	C. jejuni
2016	16-CA01693	Turkey_caecum_content	EUCAMP2	0,5	2	64	8	32	<=1	C. coli
2016	16-CA01695	Turkey_caecum_content	EUCAMP2	0,5	2	64	8	64	<=1	C. coli
2016	16-CA01696	Turkey_caecum_content	EUCAMP2	1	>16	64	>16	>64	>128	C. coli
2016	16-CA01697	Turkey_caecum_content	EUCAMP2	0,5	1	64	16	>64	2	C. coli
2016	16-CA01698	Turkey_caecum_content	EUCAMP2	0,25	1	<=1	16	64	<=1	C. jejuni
2016	16-CA01699	Turkey_caecum_content	EUCAMP2	0,5	1	8	0,5	>64	<=1	C. jejuni
2016	16-CA01700	Turkey_caecum_content	EUCAMP2	0,5	1	64	16	32	<=1	C. jejuni
2016	16-CA01701	Broiler_caecum_content	EUCAMP2	0,5	1	64	>16	>64	2	C. jejuni
2016	16-CA01702	Turkey_caecum_content	EUCAMP2	0,5	1	64	>16	>64	4	C. coli
2016	16-CA01703	Turkey_caecum_content	EUCAMP2	0,5	1	32	16	32	<=1	C. coli
2016	16-CA01704	Turkey_caecum_content	EUCAMP2	0,5	1	64	>16	>64	4	C. coli
2016	16-CA01705	Broiler_caecum_content	EUCAMP2	0,5	>16	32	8	32	<=1	C. coli
2016	16-CA01706	Broiler_caecum_content	EUCAMP2	0,5	>16	32	8	32	<=1	C. coli
2016	16-CA01707	Broiler_caecum_content	EUCAMP2	0,25	1	4	0,25	<=0,5	<=1	C. jejuni
2016	16-CA01708	Broiler_caecum_content	EUCAMP2	0,5	2	4	0,25	<=0,5	<=1	C. jejuni
2016	16-CA01709	Turkey_caecum_content	EUCAMP2	0,5	1	64	16	32	<=1	C. jejuni

Table S5: Minimum inhibitory concentrations of 6 antimicrobials for isolats of C. jejuni and C. coli from poultry

Year	Sample No.	Matrix_final	Antimicrobial panel	GEN	STR	NAL	CIP	TET	ERY	Species
2016	16-CA01710	Turkey_caecum_content	EUCAMP2	0,25	0,5	4	<=0,125	8	<=1	C. jejuni
2016	16-CA01711	Turkey_caecum_content	EUCAMP2	0,5	1	64	>16	>64	<=1	C. jejuni
2016	16-CA01712	Turkey_caecum_content	EUCAMP2	1	2	64	8	64	<=1	C. coli
2016	16-CA01713	Turkey_caecum_content	EUCAMP2	0,5	>16	32	16	>64	>128	C. coli
2016	16-CA01714	Turkey_caecum_content	EUCAMP2	0,25	1	32	8	64	<=1	C. coli
2016	16-CA01715	Turkey_caecum_content	EUCAMP2	0,5	2	>64	8	<=0,5	<=1	C. jejuni
2016	16-CA01716	Turkey_caecum_content	EUCAMP2	0,5	2	64	16	>64	2	C. coli
2016	16-CA01732	Turkey_caecum_content	EUCAMP2	0,5	2	64	16	64	2	C. jejuni
2016	16-CA01733	Turkey_caecum_content	EUCAMP2	<=0,125	>16	32	16	<=0,5	<=1	C. jejuni
2016	16-CA01735	Turkey_caecum_content	EUCAMP2	0,5	>16	32	16	32	<=1	C. jejuni
2016	16-CA01751	Broiler_caecum_content	EUCAMP2	1	2	4	<=0,125	<=0,5	<=1	C. jejuni
2016	16-CA01752	Broiler_caecum_content	EUCAMP2	0,5	1	4	<=0,125	<=0,5	<=1	C. jejuni
2016	16-CA01753	Broiler_caecum_content	EUCAMP2	0,5	1	64	16	32	4	C. coli
2016	16-CA01780	Turkey_caecum_content	EUCAMP2	0,5	1	32	>16	<=0,5	<=1	C. coli
2016	16-CA01784	Broiler_caecum_content	EUCAMP2	0,5	2	64	16	<=0,5	2	C. coli
2016	16-CA01784	Broiler_caecum_content	EUCAMP2	0,5	1	64	16	64	<=1	C. jejuni
2016	16-CA01785	Broiler_caecum_content	EUCAMP2	1	1	64	16	>64	<=1	C. jejuni
2016	16-CA01792	Broiler_caecum_content	EUCAMP2	0,5	0,5	8	0,25	<=0,5	<=1	C. jejuni
2016	16-CA01793	Broiler_caecum_content	EUCAMP2	<=0,125	<=0,25	4	<=0,125	<=0,5	<=1	C. jejuni
2016	16-CA01794	Broiler_caecum_content	EUCAMP2	0,5	1	8	0,25	<=0,5	<=1	C. jejuni
2016	16-CA01795	Broiler_caecum_content	EUCAMP2	0,5	1	8	0,25	<=0,5	<=1	C. jejuni
2016	16-CA01797	Broiler_caecum_content	EUCAMP2	0,5	1	64	16	<=0,5	<=1	C. jejuni
2016	16-CA01798	Broiler_caecum_content	EUCAMP2	0,5	1	4	<=0,125	<=0,5	<=1	C. jejuni
2016	16-CA01799	Broiler_caecum_content	EUCAMP2	0,5	1	64	8	<=0,5	<=1	C. jejuni
2016	17-CA00003	Turkey_caecum_content	EUCAMP2	0,5	1	64	>16	>64	4	C. coli
2016	17-CA00004	Turkey_caecum_content	EUCAMP2	0,5	2	>64	>16	<=0,5	2	C. jejuni
2016	17-CA00006	Broiler_caecum_content	EUCAMP2	0,5	1	32	16	32	<=1	C. jejuni
2016	17-CA00007	Broiler_caecum_content	EUCAMP2	0,5	2	32	4	4	<=1	C. coli
2016	17-CA00029	Broiler_caecum_content	EUCAMP2	0,5	1	64	16	>64	<=1	C. jejuni
2016	17-CA00092	Broiler_caecum_content	EUCAMP2	0,5	4	32	16	<=0,5	<=1	C. jejuni
2016	17-CA00093	Broiler_caecum_content	EUCAMP2	0,5	1	64	16	>64	<=1	C. jejuni
2016	17-CA00095	Broiler_caecum_content	EUCAMP2	0,5	4	>64	16	>64	<=1	C. jejuni
